# Transcriptional Hallmarks of Noonan Syndrome and Noonan-Like Syndrome with Loose Anagen Hair

**DOI:** 10.1002/humu.22026

**Published:** 2012-01-17

**Authors:** Giovanni Battista Ferrero, Gabriele Picco, Giuseppina Baldassarre, Elisabetta Flex, Claudio Isella, Daniela Cantarella, Davide Corá, Nicoletta Chiesa, Nicoletta Crescenzio, Fabio Timeus, Giuseppe Merla, Laura Mazzanti, Giuseppe Zampino, Cesare Rossi, Margherita Silengo, Marco Tartaglia, Enzo Medico

**Affiliations:** 1 Department of Pediatrics, University of Torino Medical SchoolTorino, Italy; 2 Department of Oncological Sciences, University of Torino Medical SchoolTorino, Italy; 3 Laboratory of Oncogenomics, Institute for Cancer Research and TreatmentCandiolo (Torino), Italy; 4 Dipartimento di Ematologia, Oncologia e Medicina Molecolare, Istituto Superiore di SanitàRoma, Italy; 5 Laboratory of Systems Biology, Institute for Cancer Research and Treatment (IRCC)10060 Candiolo (Torino), Italy; 6 Medical Genetics Unit, IRCCS ‘Casa Sollievo della Sofferenza’S. Giovanni Rotondo, Italy; 7 Dipartimento di Pediatria, Università degli Studi di BolognaBologna, Italy; 8 Istituto di Clinica Pediatrica, Università Cattolica del Sacro CuoreRoma, Italy; 9 UO Genetica Medica, Policlinico S.Orsola-MalpighiBologna, Italy

**Keywords:** Noonan syndrome, RASopathies, *PTPN11*, *SOS1*, *SHOC2*

## Abstract

Noonan syndrome (NS) is among the most common nonchromosomal disorders affecting development and growth. NS is genetically heterogeneous, being caused by germline mutations affecting various genes implicated in the RAS signaling network. This network transduces extracellular signals into intracellular biochemical and transcriptional responses controlling cell proliferation, differentiation, metabolism, and senescence. To explore the transcriptional consequences of NS-causing mutations, we performed global mRNA expression profiling on peripheral blood mononuclear cells obtained from 23 NS patients carrying heterozygous mutations in *PTPN11* or *SOS1*. Gene expression profiling was also resolved in five subjects with Noonan-like syndrome with loose anagen hair (NS/LAH), a condition clinically related to NS and caused by an invariant mutation in *SHOC2*. Robust transcriptional signatures were found to specifically discriminate each of the three mutation groups from 21 age- and sex-matched controls. Despite the only partial overlap in terms of gene composition, the three signatures showed a notable concordance in terms of biological processes and regulatory circuits affected. These data establish expression profiling of peripheral blood mononuclear cells as a powerful tool to appreciate differential perturbations driven by germline mutations of transducers involved in RAS signaling and to dissect molecular mechanisms underlying NS and other RASopathies. Hum Mutat 33:703–709, 2012. © 2012 Wiley Periodicals, Inc.

## Introduction

Dysregulation of RAS signaling has recently been recognized to underlie a group of clinically related disorders affecting development and growth [Schubbert et al., 2007; Tartaglia and Gelb, 2010; Tidyman and Rauen, 2009]. Most of these conditions, which are collectively named as RASopathies, share facial dysmorphism, a wide spectrum of heart disease, reduced postnatal growth, variable cognitive defects, and susceptibility to certain malignancies. In these Mendelian traits, heterozygous germline mutations affect various genes coding for members of the small subfamily of RAS GTPases, signal relay proteins that function as modulators of RAS function, RAS effectors, and downstream signal transducers. Despite the majority of mutations appear to enhance signal traffic through the RAS-mitogen-activated protein kinase (MAPK) axis, each syndrome maintains indeed distinctive phenotypic features. In some of these disorders, a further level of complexity is due to genetic heterogeneity, which explains, in part, the observed clinical variability. Noonan syndrome (NS, OMIM 163950), which is the most common among these diseases, occurring approximately in 1:1000–1:2500 live births, represents a paradigmatic condition [Allanson, 2007; Tartaglia et al., 2010; Van der Burgt, 2007]. NS is genetically heterogeneous, with activating mutations in *PTPN11*, *SOS1*, *KRAS*, *NRAS*, *RAF1*, and *BRAF* occurring in approximately 75% of affected individuals [Tartaglia et al., 2011]. NS is a clinically variable disorder, and recent studies have established clinically relevant genotype–phenotype correlations, such as a high prevalence of pulmonic stenosis among subjects with a mutated *PTPN11* allele, occurrence of hypertrophic cardiomyopathy in individuals heterozygous for a mutation in *RAF1*, or generally normal growth and cognition in subjects carrying a mutated *SOS1* gene [Tartaglia et al., 2010]. In contrast to what observed in NS, other RASopathies exhibit a relatively homogeneous phenotype that generally reflects an underlying genetic homogeneity. This is the case of Noonan-like syndrome with loose anagen hair (NL/LAH), a rare condition with clinical features partially overlapping those occurring in NS [Mazzanti et al., 2003], and caused by the invariant c.4A>G missense change (p.Ser2Gly) in *SHOC2* [Cordeddu et al., 2009], a scaffold protein with regulatory function that positively modulate RAS signaling [Matsunaga-Udagawa et al., 2010; Rodriguez-Viciana et al., 2006].

To provide first insights on the pathogenetic mechanisms underlying NS and related RASopathies, a number of studies have been directed to investigate the consequences of panels of disease-causing mutations on protein structure and function, and their perturbing effects on intracellular signaling [Tartaglia et al., 2010]. No attempt has been directed, however, to investigate the consequences of the aberrant activation of the RAS signaling network driven by the different disease-causing molecular lesions on the control of gene expression. Here, we explored the global gene expression profile of peripheral blood mononuclear cells (PBMCs) collected from two cohorts of subjects with mutations in the two most common NS disease genes (*PTPN11* and *SOS1*), and a third group representative of the genetically homogeneous NS/LAH (*SHOC2*) in order to identify transcriptional signatures specifically associated with aberrant *PTPN11*/SHP2, *SOS1,* and SHOC2 function, as well as to evaluate the extent and branching of intracellular signaling dysregulation associated with these specific pathological conditions.

## Methods

### Patients Selection

The study was approved by the Local Ethics Committee of the Regina Margherita Childrens' Hospital, Torino, Italy. Informed consent was obtained from parents or guardians of all participants. Patients were enrolled in the study between March 2006 and May 2008. Controls are children with staturo-ponderal and neuromotor development within normal limits. The diagnosis of NS was established according to Van der Burgt clinical criteria [van der Burgt et al., 1994] and confirmed by molecular analysis on genomic DNA isolated from 200 μl of peripheral blood by the QIAamp DNA Blood Mini Kit (Qiagen, Hilden, Germany). The 15 coding exons and exon/intron junctions of *PTPN11* were amplified by PCR with FastStart Taq DNA Polymerase (Roche Diagnostics Corporation, Indianapolis, IN) under standard conditions with the primers listed in Tartaglia et al. [2002], *SOS1* analysis was carried out by amplification and sequencing of the 23 exons as previously described [Tartaglia et al., 2007] and *SHOC2* gene was studied as reported in Cordeddu et al. [2009]. The study cohort included 23 subjects with a diagnosis of NS associated with a germline mutation in *PTPN11* (*N* = 17) or *SOS1* (*N* = 6), and five individuals with NS/LAH due to the invariant c.4A>G missense change in *SHOC2*. Mutation data (location of affected residues and type of amino acid substitution) are resumed in Supp. Table S1. Additional 21 samples were obtained from age- and sex-matched controls. Informed consent was obtained from all subjects included in the study.

### RNA Extraction and Processing for Microarray

RNA was extracted from PBMCs, isolated form fresh blood samples within 2 hr from collection, using the TRIzol Plus RNA purification system (Invitrogen Corp., Carlsbad, CA) according to the manufacturer's protocol. The quantification and quality analysis of RNA was performed on a Bioanalyzer 2100 (Agilent Technologies, Palo Alto, CA). Synthesis of cDNA and biotinylated cRNA was performed using the Illumina TotalPrep RNA Amplification Kit (Ambion, Foster City, CA; Cat. n. IL1791), according to the manufacturer's protocol. Quality assessment and quantification of cRNAs were performed with Agilent RNA kits on Bioanalyzer 2100. Hybridization of cRNAs (750 ng) was carried out on HumanRef8_V2 BeadChips (Illumina Inc., San Diego, CA). Array washing was performed using Illumina High stringency wash buffer for 10 min at 55°C, followed by staining using streptavidin-Cy3 dyes (Amersham Biosciences, Buckinghamshire, UK), according to standard Illumina protocols.

### Data Analysis

Cubic spline-normalized probe intensity data, together with detection *P*-values, were obtained using the BeadStudio 3.1 software (Illumina). Subsequent data processing, carried out with Excel (Microsoft Corp., Redmond, WA) included: (1) scaling, Log_2_ transformation, and detection filtering; (2) removal of genes correlated with age, sex, or differential leukocyte count; (3) Log_2_Ratio transformation and selection of genes differentially expressed between controls and mutated groups; (4) Monte carlo simulation for false discovery rate estimation; (5) full leave-one-out classification analysis. All procedures are described in detail in Supp. Methods. Log_2_Ratio expression data were clustered and visualized using the GEDAS software [Fu and Medico, 2007].

## Results

### PBMC Gene Expression Profiling of NS and NS/LAH Patients

*PTPN11*, *SOS1*, and *SHOC2* gene expression in human PBMCs was preliminarily verified by in silico analysis on a published PBMC gene expression dataset [Burczynski et al., 2006]. The analysis indicated that these and other disease genes known to be implicated in RASopathies are expressed, at varying levels, in human PBMCs (Supp. Fig. S1). For gene expression profiling (GEP), we selected 23 NS patients including 17 subjects carrying a mutation in *PTPN11* and six with a *SOS1* lesion, five NS/LAH subjects with the invariant c.4A>G *SHOC2* mutation, and 21 age- and sex-matched controls (Supp. Table S1). Total RNA extracted from PBMCs was processed for GEP on Illumina Beadarrays. We verified expression of *PTPN11*, *SOS1*, and *SHOC2* mRNA in our samples by checking microarray probe signal intensities for *PTPN11* and *SOS1*, and quantitative real-time PCR signals for *SHOC2* that was not represented on the arrays (Supp. Fig. S2). Out of the 20,589 probes analyzed on the array, 5,605 passed filtering for reliable signal detection and for not being correlated with age, sex, or differential leukocyte count (Supp. Fig. S3 and Supp. Methods). Unsupervised hierarchical clustering of all samples based on these probes revealed four major transcriptional subgroups, two of which were enriched, respectively, in NS/LAH and NS samples (Supp. Fig. S4). For supervised statistical detection of genes differentially expressed between NS and NS/LAH samples and controls, a multiple test including fold change (absolute Log_2_ ratio > 0.5), *t*-test (*P* < 0.01), and signal-to-noise ratio (SNR > 0.5; see also Supp. Methods) was applied to the following comparisons: (1) NS+NS/LAH samples versus controls; (2) *PTPN11* mutation-positive samples versus controls; (3) *SOS1* mutation-positive samples versus controls; and (4) *SHOC2* mutation-positive samples versus controls. Four signatures were thus identified, composed of 125, 225, 73, and 1407 probes, respectively (Supp. Table S2). A Monte Carlo simulation considering 2,000 random sample permutations was performed that allowed estimating the fraction of false positive hits as acceptably low (0.001–5.5%; Supp.Table S3).

### NS and NS/LAH Gene-Specific Transcriptional Signatures in Human PBMCs

**Figure 1:**
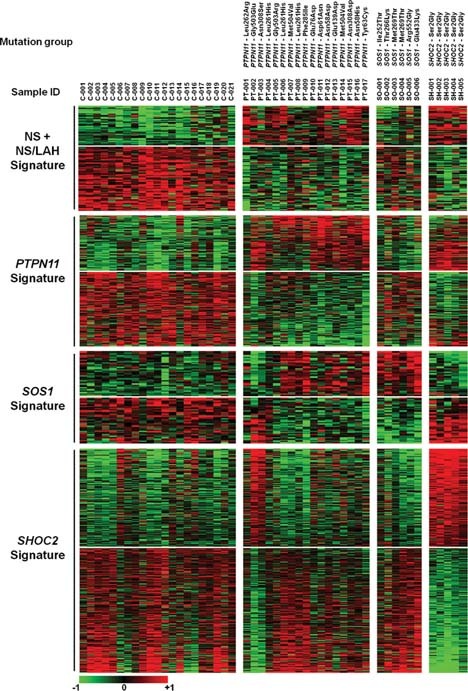
PBMC transcriptional signatures discriminating NS and NS/LAH patients from unaffected individuals. Heatmap representing Log2 ratio expression for gene probes (rows) across samples (columns). Higher than average (red) and lower than average (green) expression levels are indicated according to the color bar reported below the diagram. Samples are subdivided in four groups, from left to right: controls (C-001–C-021), NS with a mutated *PTPN11* allele (PT-001–PT-017), NS with a *SOS1* mutation (SO-001–SO-006), and NS/LAH with the c.4A>G change (SH-001–SH-005). Four major transcriptional signatures composed of genes that significantly discriminate control samples from (1) *PTPN11*, *SOS1*, and *SHOC2* mutation-positive samples (NS+NS/LAH signature, 125 gene probes), (2) *PTPN11* mutation-positive samples (*PTPN11* signature, 225 probes), (3) *SOS1* mutation-positive samples (*SOS1* signature, 73 probes), and (4) *SHOC2* mutation-positive samples (*SHOC2* signature, 1,407 probes) are shown.

Expression of genes belonging to the four signatures is shown in Figure 1. Of note, the signatures obtained separately for the *PTPN11*, *SOS1*, and *SHOC2* mutations were found to discriminate more efficiently the individual mutation groups from controls, compared to the signature characterizing the entire “RASopathy” cohort of PBMCs with mutated *PTPN11*, *SOS1*, and *SHOC2* alleles, indicating occurrence of significant heterogeneity among subgroups. Indeed, the three disease gene-specific signatures displayed detectable, but only partial overlaps (Supp. Table S2E). The signature characterizing NS/LAH was the largest, including 1,394 genes. Within this group, the expression profiles were highly homogeneous, possibly because of the invariant occurrence of the *SHOC2* c.4A>G mutation underlying this disorder, and appeared to be oppositely modulated within both the *PTPN11* and *SOS1* mutation-associated NS groups. A robust signature, characterized by 223 differently expressed genes was also attained for the *PTPN11* mutation group. This signature was shared, in part, with the *SHOC2* mutation group, while it noticeably diverged in the *SOS1* mutation group. Differently from what observed in the *PTPN11* and *SHOC2* mutation cohorts, the *SOS1* mutation group shared a signature with restricted size, which, however, appeared to efficiently discriminate this group from controls. The *SOS1* mutation-associated signature appeared oppositely modulated in the *SHOC2* mutation group, while it was relatively conserved among samples of the *PTPN11* mutation group.

Overall, these findings document that the *PTPN11*, *SOS1*, and *SHOC2* mutations induce detectable gene expression changes in PBMCs, and suggest that the specific perturbation in gene expression modulation occurring within each subgroup cannot be simply ascribed to a differential perturbing effect of mutations in individual disease genes on the extent of signal flow through a common signal transduction pathway (i.e., the RAS-MAPK cascade).

**Figure 2:**
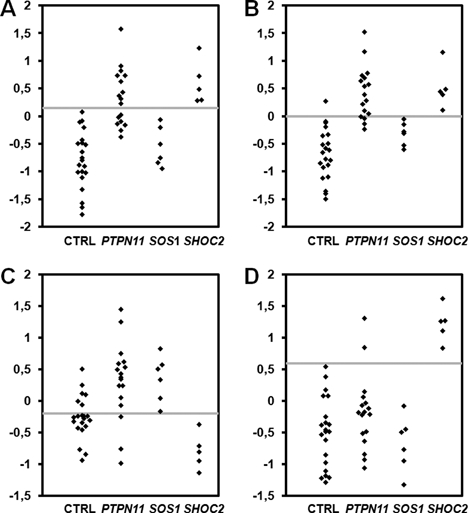
Transcriptional signatures classify PBMCs from subjects with RASopathy and unaffected individuals. The four plots show the results of a full leave-one-out classification analysis. Briefly, each PBMC sample was left out of the dataset and received four classification scores (*y*-axis, A–D) based on four signatures, fully constructed on the remaining samples: (A) NS+NS/LAH signature; (B) *PTPN11* mutation-associated signature; (C) *SOS1* mutation-associated signature; (D) *SHOC2* mutation-associated signature. Samples are subdivided in four groups, as indicated on the *x*-axis, based on the genotype. Grey horizontal lines indicate optimal putative classification thresholds. Gene-specific signatures show high discriminating ability for the respective groups of samples.

To verify whether the disease gene-specific signatures could reliably distinguish samples with mutations in the respective disease genes from control samples in a diagnostic setting, we performed full leave-one-out cross-validation analysis. Briefly, each sample (either mutated or not) was individually removed from the dataset, and the remaining samples were used to select again significant genes and redefine the four signatures. The left-out sample was then classified by calculating a weighted average score for each signature (NS+NS/LAH, *PTPN11*, *SOS1*, and *SHOC2*; see Supp. Methods). Finally, the four classification scores for each sample were displayed in dot plots (Fig. 2) and used for *t*-test-based statistics. Overall, the RASopathy-associated score was significantly different between control and NS+NS/LAH samples (*t*-test *P*-value < 0.001). It correctly classified all the *SHOC2* samples as well as the majority of *PTPN11* samples (*P* < 0.001), but failed to discern *SOS1* mutation-positive samples from controls (*P* = 0.29). The *PTPN11* mutation-associated score was documented to discriminate efficiently samples with a mutated *PTPN11* allele from healthy controls (*P* < 0.001) and those with *SOS1* mutations (*P* < 0.005), but not from samples with mutated *SHOC2* (*P* = 0.706). The *SOS1* mutation score maintained a significant discrimination efficacy against control samples (*P* < 0.001), but displayed low specificity. Finally, the *SHOC2* mutation score, despite being derived from just four samples in the “leave-one out” approach, displayed very good sensitivity and specificity against both controls (*P* < 0.001) and other mutated samples (*SHOC2* vs *PTPN11*: *P* < 0.001; *SHOC2* vs *SOS1 P* < 0.001). Overall, these results represent proof of concept that PBMC-derived transcriptional signatures are sufficiently robust to be considered as distinctive for each of the different conditions and to eventually be used for diagnostic purposes.

### In silico Data Mining Reveals Biological Significance of NS and NS/LAH Mutation-Specific Signatures

To functionally characterize genes transcriptionally associated to NS and NS/LAH causative mutations, we tested the *PTPN11*, *SOS1*, and *SHOC2* mutation-specific signatures for enrichment in functional annotation keywords using DAVID [Huang da W et al., 2009; see Supp. Methods). This analysis was conducted first using all the genes of each signature, then using subgroups of only up- or downregulated genes. As shown in Supp. Table S4, the *PTPN11* signature was found to be enriched in genes encoding proteins with SH2 domains (*P* < 0.001) and tyrosine-specific protein kinases (*P* < 0.01). The *SOS1* signature did not show significant enrichments, whereas the downregulated genes of the *SHOC2* signature were strongly enriched for genes having regulatory role in transcription (*P* < 10^−7^). These results prompted additional data mining focused on genes implicated in signal transduction and transcriptional control. The three signatures were therefore assessed for significant enrichment in kinase targets via the web-based “Kinase Enrichment Analysis” tool [Lachmann et al., 2009] (Table 1). Interestingly, this analysis documented that the *PTPN11* signature displayed highly significant enrichment in targets of tyrosine kinases, particularly SRC family kinases (FYN, LYN, LCK, SRC) and SRC family interacting kinases (CSK, SYK, ZAP70). Despite its small size, the *SOS1* signature displayed significant enrichment in substrates of LCK, while the *SHOC2* signature was enriched in targets of MAPK and SRC family members and their interacting kinases. These data show that a significant percentage of genes transcriptionally modulated by NS and NS/LAH disease-causing alleles are themselves known targets of tyrosine kinases involved in signal transduction.

**Table 1:** Enrichment of the *PTPN11, SOS1*, and *SHOC2* Signatures for Substrates of Kinases

	Kinase	Number of substrates in signature	Enrichment *P*-value
*PTPN11* signature	INSR	9	4.09E-03
	PDGFRB	5	5.81E-03
	ERBB3	6	1.57E-03
	ERBB4	4	8.43E-03
	SRC	12	2.53E-03
	LCK	13	3.72E-06
	FYN	12	2.10E-04
	LYN	10	2.28E-04
	SYK	7	1.15E-03
	CSK	5	2.31E-03
	ZAP70	5	3.49E-03
	ITK	4	3.31E-03
	BTK	5	6.64E-03
	AXL	3	9.35E-03
*SOS1* signature	LCK	5	1.84E-03
	PRKAA2	2	2.81E-03
*SHOC2* signature	PDGFRB	12	6.23E-03
	SYK	17	1.19E-03
	CSK	10	9.09E-03
	FYN	26	9.19E-03
	ZAP70	11	6.67E-03
	ITK	8	7.00E-03
	MAPK11	6	6.40E-03
	MAPK14	52	3.97E-04

Gene lists from each of the three signatures were tested on the KEA web-based tool for enrichment in substrates of kinases. The table reports only kinases whose substrates were significantly enriched (*P* < 0.01).

Subsequently, we focused on protein–protein interactions, using the Gather [Chang and Nevins, 2006] and Genes2networks [Berger et al., 2007; see Supp. Methods] web-based tools. Of note, the only protein displaying significant interactor enrichment in both *PTPN11* and *SHOC2* signatures with both tools was CBL, recently found to be mutated in a condition partially overlapping NS [Martinelli et al., 2010]. These data suggest that protein–protein interaction in silico analysis of gene expression signatures referred to the different RASopathies can represent an informative tool to identify new candidate disease genes for these disorders.

**Figure 3:**
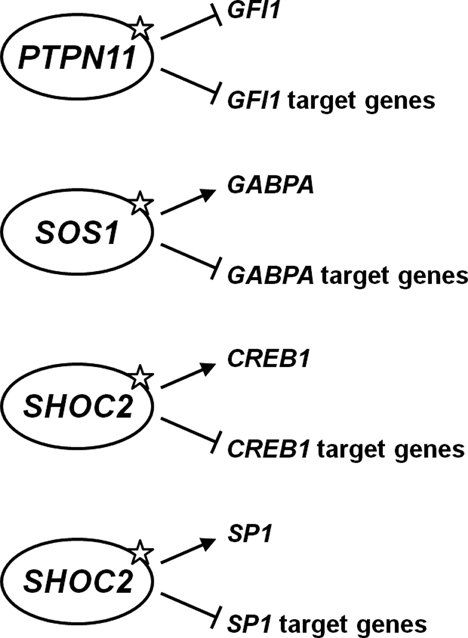
Putative circuits of transcriptional regulation in PBMCs from subjects with NS and NS/LAH. The four drawings summarize the results of transcription factor and binding site analysis conducted on lists of genes from the PBMC transcriptional signatures characterizing the *PTPN11*, *SOS1*, and *SHOC2* mutation groups. In each drawing, the oval reports, the mutated gene driving the signature, and the two links indicate concomitant and significant regulation in the same signature of a transcription factor (top link) and of its putative target genes (bottom link).

The fact that the *SHOC2* mutation was found to downregulate a large number of transcription factors (TFs) prompted us to an in-depth analysis of circuits of transcriptional regulation within the NS and NS/LAH signatures. To this aim, we searched for cases of concomitant presence within the same signature of TFs and their predicted targets. The results of this analysis, performed by the Opossum tool [Ho Sui et al., 2005], highlighted four cases of concomitant and significant coregulation (Supp. Table S5 and Supp. Methods). In *PTPN11*-mutated samples, *GFI1* was negatively regulated with respect to controls (*P* < 0.001) and its targets were preferentially downmodulated. In *SOS1*-mutated samples, *GABPA* was significantly upregulated (*P* < 0.01) and its targets were preferentially downregulated. Finally, *SHOC2*-mutated samples displayed higher expression of *CREB1* (*P* < 0.001) and *SP1* (*P* < 0.001), while the respective targets were preferentially downregulated. Overall, these results indicate the presence of at least one transcriptional regulation circuit in each signature (Fig. 3).

## Discussion

Transcriptome analysis is a key tool to explore biological complexity of human diseases. We applied this approach to RASopathies with the aim of finding molecular correlates of the mutational status in PBMCs, focusing on the two genes most frequently mutated in NS, *PTPN11*, and *SOS1*, and on *SHOC2*, which has been recently discovered to cause NS/LAH, a disorder with clinical overlap with the former [Cordeddu et al., 2009].

When grouped together and compared to age-matched unaffected individuals, NS and NS/LAH-derived samples yielded a transcriptional signature of 123 genes that correctly classified most samples. Such a signature, however, was not representative for the samples heterozygous for a mutated *SOS1* allele and a fraction of subjects with mutations in *PTPN11*. When the overall cohort of NS patients was subdivided on the basis of the genetic lesion in the three gene-specific subgroups, larger and more homogeneous signatures emerged despite the lower sizes of subgroups. These results show that, although germline mutations in *PTPN11*, *SOS1*, and *SHOC2* deregulate the RAS-MAPK pathway, each mutated gene drives specific perturbations in intracellular signaling leading to different transcriptomic changes. Leave-one-out analysis confirmed that such gene-specific signatures correctly classified most NS and NS/LAH patients, which opens the way to potential clinical diagnostic application of this approach. Partial overlap was observed between the *PTPN11* mutation-associated transcriptome and in a mutually exclusive manner, those associated with *SOS1* and *SHOC2* gene mutations, allowing to define two *PTPN11* subgroups, whose biological significance remains to be elucidated. *SOS1* and *SHOC2* mutations appeared to drive anticorrelated transcriptional changes. Interestingly, there was significantly more transcriptome perturbation in *SHOC2*-mutated specimens (1,394 genes) as compared to *PTPN11* and *SOS1*-mutated samples (223 and 73 genes, respectively). Within the *PTPN11* subgroup, no significant associations were found between transcriptional profiles and the clinical scoring system developed by van der Burgt [van der Burgt et al., 1994], possibly due to the small number of cases analyzed.

The differences highlighted by transcriptional profiling were found to be consistent with the different role of *SHP2*, *SOS1*, and *SHOC2* in modulating intracellular signaling. SHP2 is a nonreceptor protein tyrosine phosphatase [Neel et al., 2003] required for efficient activation of growth factor-induced RAS-MAPK signaling via multiple potential mechanisms [Dance et al., 2008]. Moreover, besides the positive modulatory role on RAS signaling, SHP2 controls additional signal transduction pathways, as those linked to STAT and SRC proteins that are well known to contribute significantly to transcriptional control [Grossmann et al., 2009; Xu and Qu, 2008; Zhang et al., 2004]. *SOS1* has instead a narrower role, being a bifunctional guanine nucleotide exchange factor (GEF) for RAS and RAC [Nimnual and Bar-Sagi, 2002]. This difference in function together with the possibility of a cell context specificity of the perturbing effect of mutations on intracellular signaling could explain the only partial overlap between the signatures characterizing the *SOS1*- and *PTPN11*-mutation groups. Of particular relevance is the fact that the invariant c.4A>G (p.Ser2Gly) *SHOC2* mutation was observed to provoke a profound alteration in the PBMC transcriptome. *SHOC2* encodes a widely expressed protein supposed to be required for efficient RAF1 activation following growth factor stimulation by promoting membrane translocation of the catalytic subunit of protein phosphatase 1 (PP1C) that is required for stable RAF1 binding to RAS [Rodriguez-Viciana et al., 2006]. The invariant missense change was demonstrated to introduce an *N*-myristoylation site that causes stable translocation to the plasma membrane of the mutated protein and enhanced ERK1/2 phosphorylation in a cell context-dependent fashion [Cordeddu et al., 2009]. Being a scaffold protein permanently anchored at the plasma membrane, myristylated SHOC2 may exert still uncharacterized actions leading to massive transcriptional deregulation. Intriguingly, 110 of the 225 genes composing the *PTPN11* signature are also present and concordant in the *SHOC2* signature, but most of the remaining several hundreds of the *SHOC2* signature genes display a *SHOC2*-specific behavior. This finding strongly suggests that SHOC2 might control not only the RAS-MAPK axis, but also other signaling pathways and/or cellular processes. Consistent with the present findings, it was demonstrated that SHOC2 translocates in the nucleus following growth factor stimulation [Cordeddu et al., 2009], which supports the idea of a possible direct involvement of this protein in the control of processes linked to gene expression.

Another striking finding of this work is the opposite sign of regulation of *SOS1* target genes in the *SHOC2* mutation group, and vice versa. In this case, despite the fact that both gene products in principle positively regulate the RAS-MAPK axis, gene-specific features of signal transduction apparently drive opposite transcriptional responses. A possible explanation for this paradox is that aberrant signaling by a mutated gene can be counteracted by negative feedback loops that under particular circumstances may account for most of the transcriptional changes observed at the steady-state level. According to this view, the *PTPN11*, *SOS1*, and *SHOC2* mutation-associated transcriptomes may be considered not only to directly report the grade of activity of the RAS-MAPK axis, but also highlight a more complex transcriptional circuitry that in some cases may result in opposite changes [Amit et al., 2007].

Downstream of the affected signaling pathways, NS and NS/LAH gene mutations ultimately drive functional alterations that result in clinically observable phenotypic traits. Indeed, by looking at the functions of the proteins encoded by genes included in the various above-mentioned signatures, we could reconstruct at least some of the regulatory circuits potentially involved in the molecular pathogenesis of these disorders. Basic functional keyword enrichment analysis revealed that many of the genes regulated by *PTPN11*, *SOS1*, and *SHOC2* mutations are themselves involved in signal transduction and control of transcription. Subsequent deeper analyses focused on these features highlighted interesting properties of the transcriptional targets of signaling pathways modulated by SHP2, *SOS1*, and *SHOC2*. In particular, a higher than expected representation of substrates of members of the SRC family of tyrosine kinases (FYN, LYN, LCK, SRC) was observed. This finding highlights a complex interplay between mutations in *PTPN11*, *SOS1*, and *SHOC2*, and this family of kinases known to be involved in signaling through the MAPK cascade [Zhang et al., 2004] and to regulate fundamental cellular processes such as growth, shape change, and migration in multiple cell lineages [Parsons and Parsons, 2004]. Based on these findings, it can be speculated that such an interplay could be at the basis of mesenchymal alterations giving rise to skeletal, cardiac, and hemopoietic abnormalities observed in NS and other RASopathies. Through a different approach, based on mining protein–protein interaction databases, we found that a high fraction of *PTPN11* and *SHOC2* target genes encode proteins interacting with the E3 ubiquitin ligase, CBL. Intriguingly, germline *CBL* mutations have been recently found in a condition with clinical features partially overlapping NS and with predisposition to hematologic malignancies during childhood, as well as in diverse myeloproliferative disorders and myeloid leukemias as somatically acquired lesions [Martinelli et al., 2010; Niemeyer et al., 2010; Pérez et al., 2010]. Altogether, these results reveal a highly integrated genetic program, whereby biochemical activation of the RAS-MAPK axis drives transcriptional regulation of a relevant subset of proteins involved in functionally related signaling networks. In this view, deeper exploration of transcriptome/interactome connections may highlight new candidate genes for RASopathies, not yet molecularly elucidated.

Finally, we focused on TF/target gene circuits modulated by the *PTPN11*, *SOS1*, and *SHOC2* mutations. The most interesting one involves *GFI1* (Growth factor independence 1) that was negatively modulated in samples with mutated *PTPN11* and whose predicted targets were concordantly downregulated in the same samples. Notably, children with NS present increased risk of myeloproliferative disorder (MPD) [Kratz et al., 2005], and GFI1 loss of function has been documented to cause MPDs [Khandanpour et al., 2011]. This evidence suggests that *GFI1* downmodulation could be causally linked to MPD susceptibility in NS. In principle, genes differentially expressed in PBMCs could also be regulated in other tissues, and, therefore, related to nonhematologic anomalies. As an example, altered gene expression in the blood has been found to correlate with Huntington's disease, a specific neurodegenerative autosomal dominant disorder [Runne et al., 2007]. Indeed, *GFI1* is also involved in the development of the inner ear hair cells [Möröy T., 2005]*,* and its mRNA was robustly downregulated in two independent murine models of hearing loss [Hertzano et al., 2004, Lewis et al., 2009]. In this respect, *PTPN11* mutation-driven *GFI1* downregulation could play a key role in hearing abnormalities observed in NS [Scheiber et al., 2009; Qiu et al.,^1998^]. In the *SHOC2* signature, two TFs, *CREB1* and *SP1* were consistently upregulated, while their targets resulted to be preferentially downmodulated. *CREB1* encodes a 43-kDa basic/leucine zipper (bZIP) TF known to be a target of the MAPK/ERK pathway [Morgan et al., 2001]. Interestingly, hippocampi deriving from a knockout mouse model of neurofibromatosis presented increased activity of the RAF-ERK axis and of CREB [Guilding et al., 2007], indicating a possible involvement in the pathogenesis of cognitive impairment observed in RASopathies. *SP1* belongs to the SP/KLF TF family and is a MAPK target [Benasciutti et al., 2004; Curry et al., 2008]. Interestingly, enhanced *SP1* activity has been linked to cardiac hypertrophy, a recurrent cardiac anomaly in NS [Azakie et al., 2006; Hu et al., 2010; Lin et al., 2009]. Finally, the putative *GABPA* circuit detected in the *SOS1* signature is consistent with the fact that *GABPA* is a known target of the MAPK pathway [Flory et al., 1996; Fromm and Burden, 2001]. Altogether, functional data mining focused on signal transduction and TF activity highlighted genes and modules of transcriptional regulation present in the *PTPN11*, *SOS1*, and *SHOC2* signatures that provide useful hints on the molecular pathogenesis of NS.

It is likely that current advances in massive sequencing will pave the way to molecular characterization of all germline mutations causing RASopathies. In this perspective, the clinical potential of transcriptional NS signatures will not reside as much on first diagnosis applications, but rather on its utility as a transcriptional readout of the actual functional status of the affected tissue. In this view, the results shown here open the way to exploit PBMC gene signatures as surrogate markers of specific MAPK pathway activation driven by NS gene mutations and, therefore, as a powerful tool to monitor the biological response to molecular targeted drugs.

## References

[b1] Allanson JE (2007). Noonan syndrome. Am J Med Genet C Semin Med Genet.

[b2] Amit I, Citri A, Shay T, Lu Y, Katz M, Zhang F, Tarcic G, Siwak D, Lahad J, Jacob Hirsch J, Amariglio N, Vaisman N, Segal E, Rechavi G, Alon U, Mills GB, Domany E, Yarden Y (2007). A module of negative feedback regulators defines growth factor signaling. Nat Genet.

[b3] Azakie A, Fineman JR, He Y (2006). Myocardial transcription factors are modulated during pathologic cardiac hypertrophy in vivo. J Thorac Cardiovasc Surg.

[b4] Benasciutti E, Pagès G, Kenzior O, Folk W, Blasi F, Crippa MP (2004). MAPK and JNK transduction pathways can phosphorylate Sp1 to activate the uPA minimal promoter element and endogenous gene transcription. Blood.

[b5] Berger SI, Posner JM, Ma'ayan A (2007). Genes2Networks: connecting lists of gene symbols using mammalian protein interactions databases. BMC Bioinformatics.

[b6] Burczynski ME, Peterson RL, Twine NC, Zuberek KA, Brodeur BJ, Casciotti L, Maganti V, Reddy PS, Strahs A, Immermann F, Spinelli W, Schwertschlag U, Slager AM, Cotreau MM, Dorner AJ (2006). Molecular classification of Crohn's disease and ulcerative colitis patients using transcriptional profiles in peripheral blood mononuclear cells. J Mol Diagn.

[b7] Chang JT, Nevins JR (2006). GATHER: a systems approach to interpreting genomic signatures. Bioinformatics.

[b8] Cordeddu V, Di Schiavi E, Pennacchio LA, Ma'ayan A, Sarkozy A, Fodale V, Cecchetti S, Cardinale A, Martin J, Schackwitz W, Lipzen A, Zampino G, Mazzanti L, Digilio MC, Martinelli S, Flex E, Lepri F, Bartholdi D, Kutsche K, Ferrero GB, Anichini C, Selicorni A, Rossi C, Tenconi R, Zenker M, Merlo D, Dallapiccola B, Iyengar R, Bazzicalupo P, Gelb BD, Tartaglia M (2009). Mutation of SHOC2 promotes aberrant protein N-myristoylation and causes Noonan-like syndrome with loose anagen hair. Nat Genet.

[b9] Curry JM, Eubank TD, Roberts RD, Wang Y, Pore N, Maity A, Marsh CB (2008). M-CSF signals through the MAPK/ERK pathway via Sp1 to induce VEGF production and induces angiogenesis in vivo. PLoS One.

[b10] Dance M, Montagner A, Salles JP, Yart A, Raynal P (2008). The molecular functions of Shp2 in the Ras/Mitogen-activated protein kinase (ERK1/2) pathway. Cell Signal.

[b11] Flory E, Hoffmeyer A, Smola U, Rapp UR, Bruder JT (1996). Raf-1 kinase targets GA-binding protein in transcriptional regulation of the human immunodeficiency virus type 1 promoter. J Virol.

[b12] Fromm L, Burden SJ (2001). Neuregulin-1-stimulated phosphorylation of GABP in skeletal muscle cells. Biochemistry.

[b13] Fu L, Medico E (2007). FLAME, a novel fuzzy clustering method for the analysis of DNA microarray data. BMC Bioinformatics.

[b14] Grossmann KS, Wende H, Paul FE, Cheret C, Garratt AN, Zurborg S, Feinberg K, Besser D, Schulz H, Peles E, Selbach M, Birchmeier W, Birchmeier C (2009). The tyrosine phosphatase Shp2 (PTPN11) directs Neuregulin-1/ErbB signaling throughout Schwann cell development. Proc Natl Acad Sci USA.

[b15] Guilding C, McNair K, Stone TW, Morris BJ (2007). Restored plasticity in a mouse model of neurofibromatosis type 1 via inhibition of hyperactive ERK and CREB. Eur J Neurosci.

[b16] Hertzano R, Montcouquiol M, Rashi-Elkeles S, Elkon R, Yücel R, Frankel WN, Rechavi G, Möröy T, Friedman TB, Kelley MW, Avraham KB (2004). Transcription profiling of inner ears from Pou4f3(ddl/ddl) identifies Gfi1 as a target of the Pou4f3 deafness gene. Hum Mol Genet.

[b17] Ho Sui SJ, Mortimer JR, Arenillas DJ, Brumm J, Walsh CJ, Kennedy BP, Wasserman WW (2005). oPOSSUM: identification of over-represented transcription factor binding sites in co-expressed genes. Nucleic Acids Res.

[b18] Hu X, Li T, Zhang C, Liu Y, Xu M, Wang W, Jia Z, Ma K, Zhang Y, Zhou C (2010). GATA4 regulates ANF expression synergistically with Sp1 in a cardiac hypertrophy model. J Cell Mol Med.

[b19] Huang da W, Sherman BT, Lempicki RA (2009). Systematic and integrative analysis of large gene lists using DAVID bioinformatics resources. Nat Protoc.

[b20] Khandanpour C, Kosan C, Gaudreau MC, Dührsen U, Hébert J, Zeng H, Möröy T (2011). Growth factor independence 1 (Gfi1) protects hematopoietic stem cells against apoptosis but also prevents the development of a myeloproliferative-like disease. Stem Cells.

[b21] Kratz CP, Niemeyer CM, Castleberry RP, Cetin M, Bergsträsser E, Emanuel PD, Hasle H, Kardos G, Klein C, Kojima S, Stary J, Trebo M, Zecca M, Gelb BD, Tartaglia M, Loh ML (2005). The mutational spectrum of PTPN11 in juvenile myelomonocytic leukemia and Noonan syndrome/myeloproliferative disease. Blood.

[b22] Lachmann A, Ma'ayan A (2009). KEA: kinase enrichment analysis. Bioinformatics.

[b23] Lewis MA, Quint E, Glazier AM, Fuchs H, De Angelis MH, Langford C, van Dongen S, Abreu-Goodger C, Piipari M, Redshaw N, Dalmay T, Moreno-Pelayo MA, Enright AJ, Steel KP (2009). An ENU-induced mutation of miR-96 associated with progressive hearing loss in mice. Nat Genet.

[b24] Lin H, Xiao J, Luo X, Chen G, Wang Z (2009). Transcriptional control of pacemaker channel genes HCN2 and HCN4 by Sp1 and implications in re-expression of these genes in hypertrophied myocytes. Cell Physiol Biochem.

[b25] Martinelli S, De Luca A, Stellacci E, Rossi C, Checquolo S, Lepri F, Caputo V, Silvano M, Buscherini F, Consoli F, Ferrara G, Digilio MC, Cavaliere ML, van Hagen JM, Zampino G, van der Burgt I, Ferrero GB, Mazzanti L, Screpanti I, Yntema HG, Nillesen WM, Savarirayan R, Zenker M, Dallapiccola B, Gelb BD, Tartaglia M (2010). Heterozygous germline mutations in the CBL tumor-suppressor gene cause a Noonan syndrome-like phenotype. Am J Hum Genet.

[b26] Matsunaga-Udagawa R, Fujita Y, Yoshiki S, Terai K, Kamioka Y, Kiyokawa E, Yugi K, Aoki K, Matsuda M (2010). The scaffold protein Shoc2/SUR-8 accelerates the interaction of Ras and Raf. J Biol Chem.

[b27] Mazzanti L, Cacciari E, Cicognani A, Bergamaschi R, Scarano E, Forabosco A (2003). Noonan-like syndrome with loose anagen hair: a new syndrome. Am J Med Genet A.

[b28] Morgan MA, Dolp O, Reuter CW (2001). Cell-cycle-dependent activation of mitogen-activated protein kinase kinase (MEK-1/2) in myeloid leukemia cell lines and induction of growth inhibition and apoptosis by inhibitors of RAS signaling. Blood.

[b29] Möröy T (2005). The zinc finger transcription factor Growth factor independence 1 (Gfi1). Int J Biochem Cell Biol.

[b30] Neel BG, Gu H, Pao L (2003). The ‘Shp’ing news: SH2 domain-containing tyrosine phosphatases in cell signaling. Trends Biochem Sci.

[b31] Niemeyer CM, Kang MW, Shin DH, Furlan I, Erlacher M, Bunin NJ, Bunda S, Finklestein JZ, Sakamoto KM, Gorr TA, Mehta P, Schmid I, Kropshofer,Corbacioglu S, Lang PJ, Klein C, Schlegel PG, Heinzmann A, Schneider M, Starý J, van den Heuvel-Eibrink MM, Hasle H, Locatelli F, Sakai D, Archambeault S, Chen L, Russell RC, Sybingco SS, Ohh M, Braun BS, Flotho C, Loh ML (2010). Germline CBL mutations cause developmental abnormalities and predispose to juvenile myelomonocytic leukemia. Nat Genet.

[b32] Nimnual A, Bar-Sagi D (2002). The two hats of SOS. Sci STKE.

[b33] Parsons SJ, Parsons JT (2004). Src family kinases, key regulators of signal transduction. Oncogene.

[b34] Pérez B, Mechinaud F, Galambrun C, Ben Romdhane N, Isidor B, Philip N, Derain-Court J, Cassinat B, Lachenaud J, Kaltenbach S, Salmon A, Désirée C, Pereira S, Menot ML, Royer N, Fenneteau O, Baruchel A, Chomienne C, Verloes A, Cavé H (2010). Germline mutations of the CBL gene define a new genetic syndrome with predisposition to juvenile myelomonocytic leukaemia. J Med Genet.

[b35] Qiu WW, Yin SS, Stucker FJ (1998). Audiologic manifestations of Noonan syndrome. Otolaryngol Head Neck Surg.

[b36] Rodriguez-Viciana P, Oses-Prieto J, Burlingame A, Fried M, McCormick F (2006). A phosphatase holoenzyme comprised of Shoc2/Sur8 and the catalytic subunit of PP1 functions as an M-Ras effector to modulate Raf activity. Mol Cell.

[b37] Runne H, Kuhn A, Wild EJ, Pratyaksha W, Kristiansen M, Isaacs JD, Régulier E, Delorenzi M, Tabrizi SJ, Luthi-Carter R (2007). Analysis of potential transcriptomic biomarkers for Huntington's disease in peripheral blood. Proc Natl Acad Sci USA.

[b38] Scheiber C, Hirschfelder A, Gräbel S, Peters H, Olze H (2009). Bilateral cochlear implantation in children with Noonan syndrome. Int J Pediatr Otorhinolaryngol.

[b39] Schubbert S, Shannon K, Bollag G (2007). Hyperactive Ras in developmental disorders and cancer. Nat Rev Cancer.

[b40] Tartaglia M, Gelb BD (2007). Disorders of dysregulated signal traffic through the RAS-MAPK pathway: phenotypic spectrum and molecular mechanisms. Ann NY Acad Sci.

[b41] Tartaglia M, Gelb BD, Zenker M (2011). Noonan syndrome and clinically related disorders. Best Pract Res Clin Endocrinol Metab.

[b42] Tartaglia M, Kalidas K, Shaw A, Song X, Musat DL, van der Burgt I, Brunner HG, Bertola DR, Crosby A, Ion A, Kucherlapati RS, Jeffery S, Patton MA, Gelb BD (2002). PTPN11 mutations in Noonan syndrome: molecular spectrum, genotype–phenotype correlation, and phenotypic heterogeneity. Am J Hum Genet.

[b43] Tartaglia M, Pennacchio LA, Zhao C, Yadav KK, Fodale V, Sarkozy A, Pandit B, Oishi K, Martinelli S, Schackwitz W, Ustaszewska A, Martin J, Bristow J, Carta C, Lepri F, Neri C, Vasta I, Gibson K, Curry CJ, Siguero JP, Digilio MC, Zampino G, Dallapiccola B, Bar-Sagi D, Gelb BD (2007). Gain-of-function SOS1 mutations cause a distinctive form of Noonan syndrome. Nat Genet.

[b44] Tartaglia M, Zampino G, Gelb BD (2010). Noonan syndrome: clinical aspects and molecular pathogenesis. Mol Syndromol.

[b45] Tidyman WE, Rauen KA (2009). The RASopathies: developmental syndromes of Ras/MAPK pathway dysregulation. Curr Opin Genet Dev.

[b46] Van der Burgt I (2007). Noonan syndrome. Orphanet J Rare Dis.

[b47] Van der Burgt I, Berends E, Lommen E, van Beersum S, Hamel B, Mariman E (1994). Clinical and molecular studies in a large Dutch family with Noonan syndrome. Am J Med Genet.

[b48] Xu D, Qu CK (2008). Protein tyrosine phosphatases in the JAK/STAT pathway. Front Biosci.

[b49] Zhang SQ, Yang W, Kontaridis MI, Bivona TG, Wen G, Araki T, Luo J, Thompson JA, Schraven BL, Philips MR, Neel BG (2004). Shp2 regulates SRC family kinase activity and Ras/Erk activation by controlling Csk recruitment. Mol Cell.

