# Supplementary material for: Transcriptional Hallmarks of Noonan Syndrome and Noonan-Like Syndrome with Loose Anagen Hair
Source: Hum Mutat. 2012 Jan 17;33(4):703–9. doi: 10.1002/humu.22026 (PMC3332054; doi:10.1002/humu.22026)
Supplement: Supplementary file 1 [file humu0033-0703-SD1.pdf]

Transcriptional Hallmarks of Noonan Syndrome and Noonan-Like Syndrome with Loose Anagen Hair in Human Peripheral Blood Cells

G.B. Ferrero, G. Picco, G. Baldassarre, E. Flex, C. Isella, D. Cantarella, D. Corà, N. Chiesa, N. Crescenzio, F. Timeus, G. Merla, L. Mazzanti, G. Zampino, C. Rossi, M. Silengo, M. Tartaglia, E. Medico

Supporting Information

Supp. Methods 3
I. Microarray data processing and filtering 3
a. Scaling, Log2 transformation and detection filtering. 3
b. Removal of genes correlated with age, sex, or differential leukocyte count. 3
c. Log_2 ratio transformation 3
II. Microarray data analysis 4
a. Selection of genes differentially expressed between controls and mutated groups. 4
b. Montecarlo simulation for FDR estimation. 4
c. Weighted average score for full leave-one-out classification analysis. 4
III. Functional data mining 4
a. Functional annotation analysis 4
b. Kinase Enrichment analysis 4
c. Protein-protein interactions analysis 5
d. Binding site analysis and transcriptional circuits 5
Supp. Figures 6
Supp. Figure S1 .... **Error! Bookmark not defined.**
Supp. Figure S2 .... **Error! Bookmark not defined.**
Supp. Figure S3 .... **Error! Bookmark not defined.**
Supp. Figure S4 .... **Error! Bookmark not defined.**

Supp. Tables....................9
Supp. Table S1 .... **Error! Bookmark not defined.**
Supp. Table S2A .... **Error! Bookmark not defined.**
Supp. Table S2B.................... **Error! Bookmark not defined.**
Supp. Table S2C.................... **Error! Bookmark not defined.**
Supp. Table S2D .... **Error! Bookmark not defined.**
Supp. Table S2E....................73
Supp. Table S3 .... **Error! Bookmark not defined.**
Supp. Table S4A .... **Error! Bookmark not defined.**
Supp. Table S4B.................... **Error! Bookmark not defined.**
Supp. Table S5 .... **Error! Bookmark not defined.**

Supp. Methods

I. Microarray data processing and filtering

a. Scaling, Log2 transformation and detection filtering.

Cubic spline-normalized probe signals, together with detection p-values, were obtained using the Illumina BeadStudio 3.1 software with background subtraction. To avoid negative values, the fixed amount of 50 was added back to each data point prior to $\log_2$ transformation. Subsequent data filtering and analysis was carried out with Excel (Microsoft). To the 20584 probes analyzed, we applied a statistical filter to select genes with reliable signal detection, selecting genes with a detection value (as provided by BeadStudio) greater than 0.999 in at least 5 samples.

b. Removal of genes correlated with age, sex, or differential leukocyte count.

To filter out genes correlated to basic clinical features, we collected information on gender, age (years), white blood cells and lymphocyte counts for the NS samples presented in the Table below.

ID	01	34	36	40	02	13	05	17	29	11	16	28	08	20	41	56
Gender (1=F, -1=M)	1	-1	1	1	-1	-1	1	1	-1	-1	1	1	1	-1	-1	-1
AGE (yrs.)	7.06	1.23	5.99	12.83	20.92	3.18	16.21	5.66	19.01	36.16	3.01	6.46	4.21	1.85	4.56	1.77
WBC	8.41				9.12	13.7	10.7	8.11	8.67		14.8	6.16	6.78	10.3		
LY%	12.3				29.3	46.3	17.9	32.1	32.9		62.8	43.5	53.3	79.4		

For each detected gene, we calculated the Pearson correlation with each of the four clinical variables. Probes displaying a Pearson correlation higher than 0.5 with any of the clinical variables were removed from subsequent analyses, which left a total of 5605 probes employed for further analysis.

c. Log_2 ratio transformation

For each probe, the $\log_2$ signal in each sample was converted into $\log_2$ ratio against global average expression of that probe in all samples. To avoid distortions due to the different sample sizes (from $n = 5$ for *SHOC2* samples to $n = 21$ for controls), the global average was calculated by first averaging the $\log_2$ signal within each group (CTRL, *PTPN11*, *SOS1* and *SHOC2*) and then by further averaging these four means. The $\log_2$ ratio was then calculated by subtracting $\log_2$ signal of the single sample from the global average.

II. Microarray data analysis

a. Selection of genes differentially expressed between controls and mutated groups.

To be defined as differential between controls and mutated groups, a probe Log_2 ratio signal had to display significance in three basic tests: (i) log_2 ratio between the means in control and mutated group greater than 0.5 (positive or negative); (ii) independent samples, two-way T-test p-value lower than 0.01; (iii) signal-to-noise ratio greater than 0.5 (positive or negative), according to the formula described by Golub and colleagues (Science 286:531-537, 1999).

b. Montecarlo simulation for FDR estimation.

To estimate the False Discovery Rate, i.e. the fraction of false positives expected for the four signatures described in the main text, 2000 orthogonal random permutations of the samples were performed, whereby for each cycle of permutation the three statistical filters employed for genes selection we re-applied, recording the number of significant probes for each of the four signatures. For each signature, median and mean false positives were then compared with true positives as illustrated in Supp. Table S3.

c. Weighted average score for full leave-one-out classification analysis.

After removing a sample from the dataset and redefining the four signatures, the left-out sample was classified by a weighted average score, obtained by subtracting average log2 ratios of probes with higher signal in control samples from average log2 ratio of probes with higher signal in mutated samples.

III. Functional data mining

a. Functional annotation analysis

To define functional annotation keyword enrichment in the mutation-specific signatures, we uploaded the lists of Illumina probes belonging to the *PTPN11*, *SOS1* and *SHOC2* signatures on the David Ease web-based annotation tool (http://david.abcc.ncifcrf.gov/). Three types of lists were uploaded for each signature: (i) all probes, both up- and down-regulated in mutated samples; (ii) only probes up-regulated in mutated samples; (iii) only probes down-regulated in mutated samples. As a background list, we employed the 5605 probes passing filtering criteria mentioned in the Results section. Subsequently the various lists underwent “Functional Annotation Clustering” analysis, returning Benjamini corrected enrichment p-values for functional clusters. The output of this analysis was filtered to include only results with corrected p-values of less than 0.01.

b. Kinase Enrichment analysis

To identify substrates of kinases in the signatures, lists of gene symbols corresponding to Illumina probes of the *PTPN11*, *SOS1* and *SHOC2* signatures were uploaded on the web-based Kinase Enrichment Analysis tool (*http://amp.pharm.mssm.edu/lib/kea.jsp*). The output of this analysis was filtered to include only results with kinase enrichment p-values of less than 0.01.

c. Protein-protein interactions analysis

To check the signatures for enrichment in protein-protein interactions, we employed two web-based tools, Gather *(http://gather.genome.duke.edu/)* and Gene2Network (*http://actin.pharm.mssm.edu/genes2networks/*). The first employs data from two large-scale protein-protein interaction studies in humans, and the second integrates multiple small-scale, high-quality protein-protein interaction datasets. Lists of gene symbols corresponding to Illumina probes of the *PTPN11*, *SOS1* and *SHOC2* signatures were uploaded on the two websites and “in silico” protein-protein interaction analysis was performed. To achieve complete independence between the two analyses, Gene2Network analysis was conducted excluding the “Vidal and Stelzl” dataset, which is by default employed by Gather. Addictional parameters of Gene2Network analysis were the following: (i) max path length threshold =2; (ii) significance cut-off =3; (iii) max interaction for reference=1; (iv) min reference per interaction=no filter.

d. Binding site analysis and transcriptional circuits

To analyze transcription factor activity, a curated, non-redundant list of transcription factors (TFs) was extracted from the JASPAR CORE database (http://jaspar.genereg.net/). This list of TFs was then filtered to maintain only those TFs with available binding site data in the Opossum tool (http://www.cisreg.ca/cgi-bin/oPOSSUM/opossum). Eleven of these TFs were found in one or more of the three signatures: *GFI1* (*PTPN11* and *SOS1* signatures), *GABPA* (*SOS1* and *SHOC2* signatures), and nine TFs only in the *SHOC2* signature (*CEBPA*, *CREB1*, *ELK1*, *ELK4*, *MAX*, *NR3C1*, *SP1*, *STAT1* and *ZNF395*). For each of these eleven TFs, we verified, if the respective *PTPN11*, *SOS1* or *SHOC2* signature was enriched in genes containing one or more specific TF binding sites. To this aim, we employed the “Custom binding site analysis” of Opossum, uploading lists of gene symbols corresponding to Illumina probes of the *PTPN11*, *SOS1* or *SHOC2* signatures, subdivided in up-regulated and down-regulated in mutated samples. As a background list, we employed the 5605 probes passing filtering criteria mentioned in the Results section. The amount of upstream / downstream sequence was set to (2000/0) and Matrix match threshold to 80%. We considered significant Opossum Z-Scores greater than 5 and p-values lower than 0.05. Putative transcriptional circuits highlighted by Opossum and including a TF and its enriched targets in the same signature were consolidated by a complementary procedure, that involved for each given TF: (i) retrieval, using Opossum, of all genes containing binding sites for that TF (putative targets), in the 5605-gene background list and in the signature(s) containing the TF; (ii) hypergeometric distribution analysis of enrichment of putative target genes in the signature vs. background, with subsequent p-value calculation. Results of both analyses are shown in Supp. Table S5.

Supp. Figures

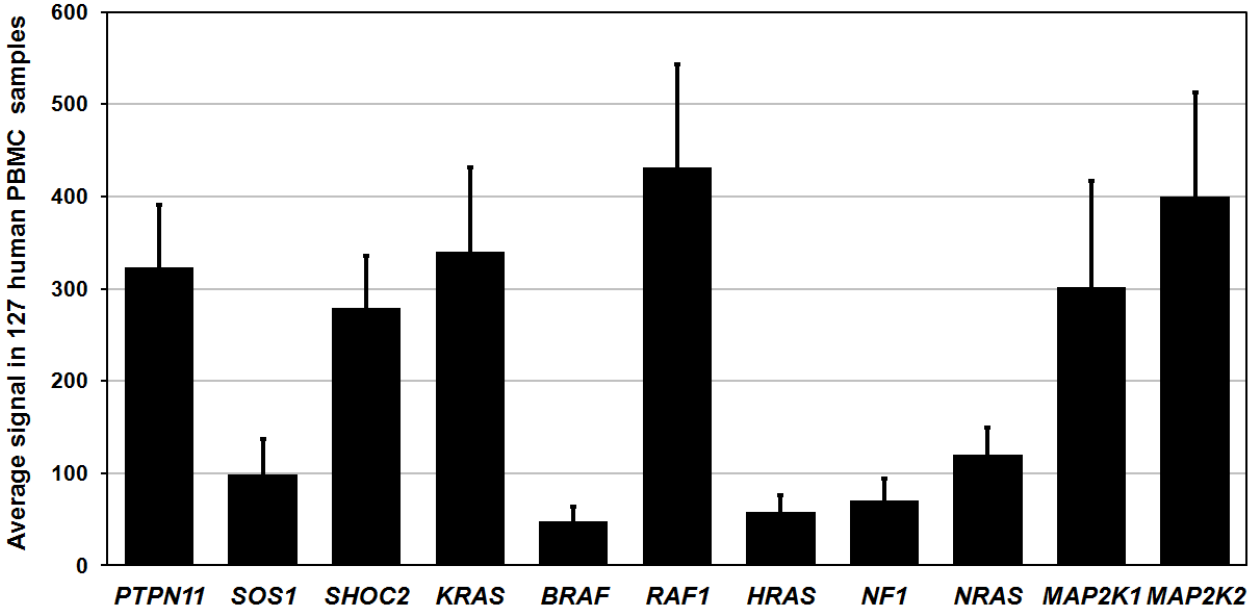

Supp. Figure S1. Expression levels in human PBMCs of RAS/MAPK pathway genes mutated in RASopathies. Histograms represent average expression signal in 127 human PBMC samples (Burczynski et all, J Mol Diagn. 2006). *RAF1*, *MAP2K1*, *MAP2K2*, *KRAS*, *SHOC2* and *PTPN11* genes show high level of expression, while *SOS1*, *NF1*, *HRAS* and *BRAF* present lower, but detectable expression values.

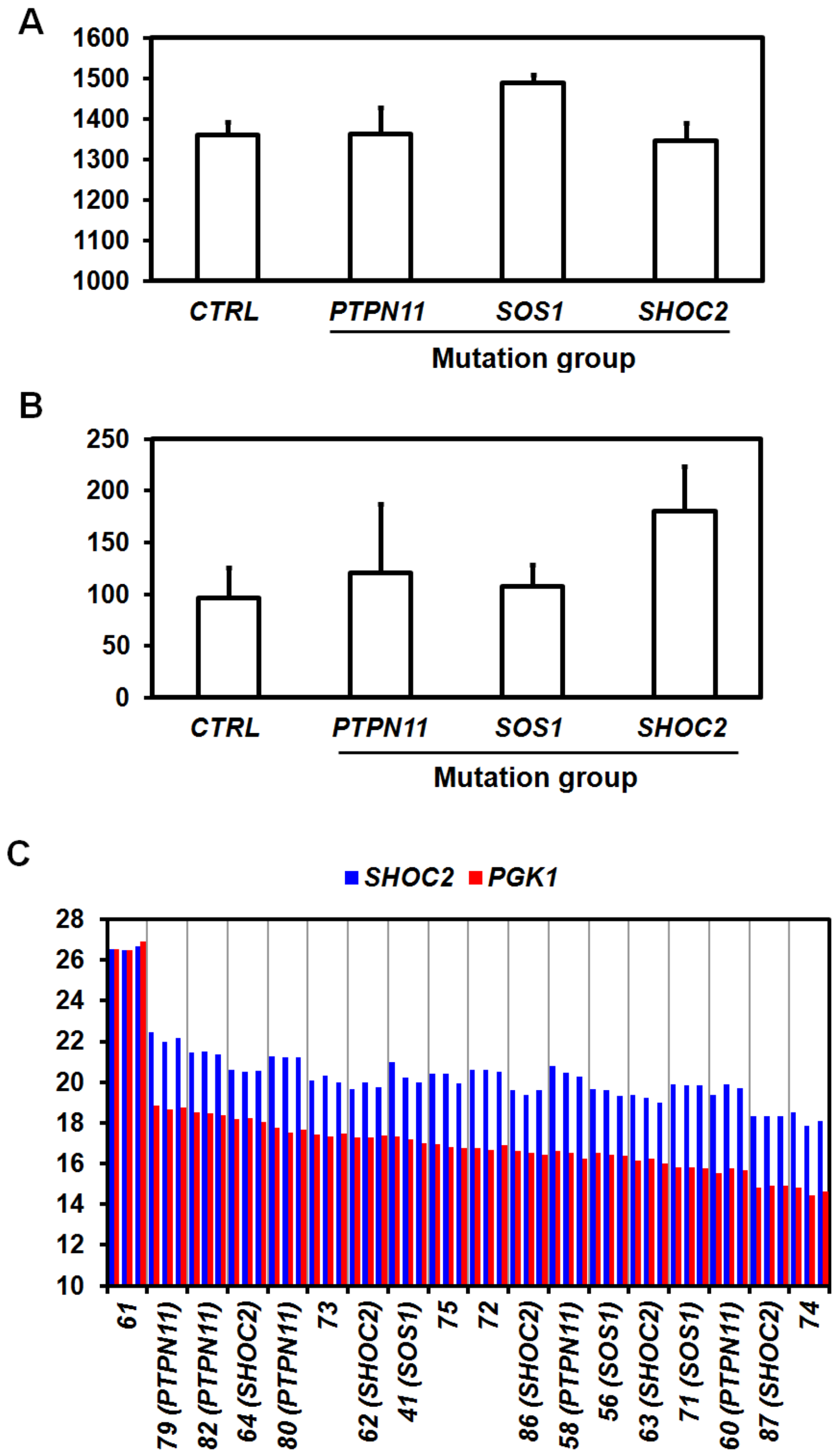

Supp. Figure S2. Expression levels in human PBMCs of *PTPN11*, *SOS1* and *SHOC2*. For *PTPN11* and *SOS1* (A, B), histograms represent average expression signal in our 49-sample human PBMC samples profiled with Illumina gene expression arrays. For *SHOC2*, quantitative realtime PCR triplicate Ct values are reported for *SHOC2* and the housekeeper *PGK1* in 18 PBMC samples chosen among controls, NS and NSL-LAH, as indicated.

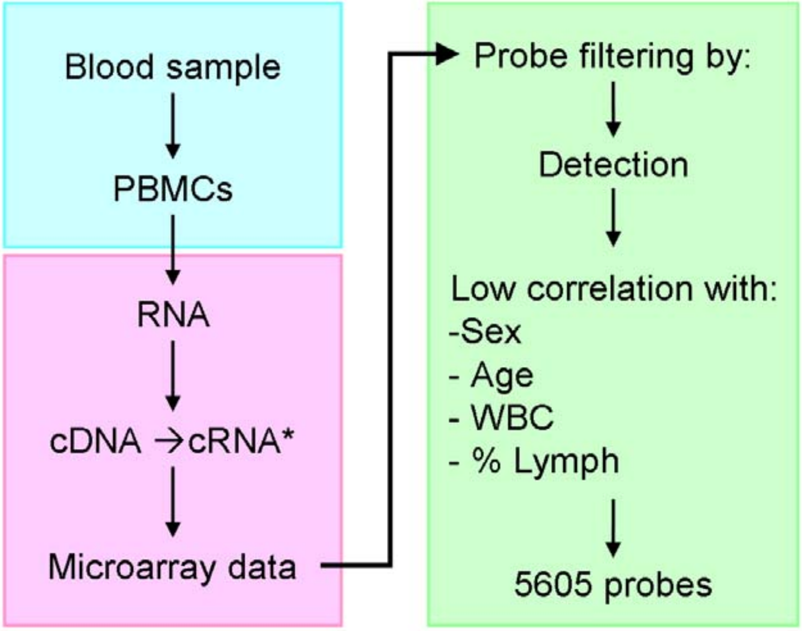

Supp. Figure S3. Experimental workflow for PBMC purification, expression profiling, and data filtering. The workflow is subdivided in three main parts: blood processing (light blue), RNA extraction and gene expression profiling (pink), and data processing for gene filtering (light green).

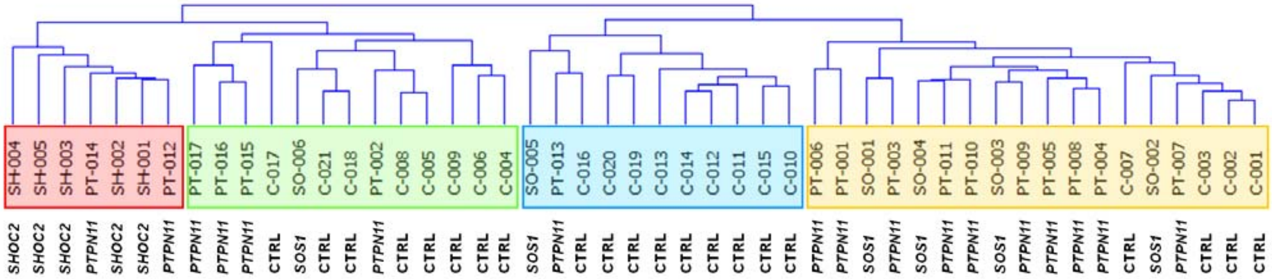

Supp. Figure S4. Unsupervised cluster analysis. Hierarchical clustering of PBMC samples from: (i) 21 control subjects (C-001-021), (ii) 17 NS patients with a *PTPN11* mutation (PT-001-017), (iii) 6 NS patients with a *SOS1* mutation (SO-001-006), and (iv) 6 NS/LAH patients with the *SHOC2* c.4A>G mutation (SH-001-005). Four major transcriptional clusters emerge, two of which are clearly enriched in *SHOC2* samples and *PTPN11* + *SOS1* samples, respectively.

Supp. Tables

Supp. Table S1. Genotypes of NS and NS/LAH patients			
**Patient ID**	**Gene**	**Nucleotide substitution**	**Amino-acid substitution**
**PT-001**	*PTPN11*	c.785T>G	p.Leu262Arg
**PT-002**	*PTPN11*	c.1508G>A	p.Gly503Glu
**PT-003**	*PTPN11*	c.923A>G	p.Asn308Ser
**PT-004**	*PTPN11*	c.782T>A	p.Leu261His
**PT-005**	*PTPN11*	c.1507G>A	p.Gly503Arg
**PT-006**	*PTPN11*	c.782T>A	p.Leu261His
**PT-007**	*PTPN11*	c.1510A>G	p.Met504Val
**PT-008**	*PTPN11*	c.782T>A	p.Leu261His
**PT-009**	*PTPN11*	c.853T>A	p.Phe285Ile
**PT-010**	*PTPN11*	c.228G>C	p.Glu76Asp
**PT-011**	*PTPN11*	c.181G>A	p.Asp61Asn
**PT-012**	*PTPN11*	c.172A>G	p.Asn58Asp
**PT-013**	*PTPN11*	c.417G>T	p.Glu139Asp
**PT-014**	*PTPN11*	c.1510A>G	p.Met504Val
**PT-015**	*PTPN11*	c.922A>G	p.Asn308Asp
**PT-016**	*PTPN11*	c.172A>C	p.Asn58His
**PT-017**	*PTPN11*	c.188A>G	p.Tyr63Cys
**SO-001**	*SOS1*	c.755T>C	p.Ile252Thr

**SO-002**	*SOS1*	c.797C>A	p.Thr266Lys
**SO-003**	*SOS1*	c.806T>C	p.Met269Thr
**SO-004**	*SOS1*	c.806T>C	p.Met269Thr
**SO-005**	*SOS1*	c.1654A>G	p.Arg552Gly
**SO-006**	*SOS1*	c.1297G>A	p.Glu433Lys
**SH-001**	*SHOC2*	c.4A>G	p.Ser2Gly
**SH-002**	*SHOC2*	c.4A>G	p.Ser2Gly
**SH-003**	*SHOC2*	c.4A>G	p.Ser2Gly
**SH-004**	*SHOC2*	c.4A>G	p.Ser2Gly
**SH-005**	*SHOC2*	c.4A>G	p.Ser2Gly

GenBank reference sequences:

PTPN11: NM_002834.3; ***SOS1***: NM_005633.3; ***SHOC2***: NM_007373.3

Nucleotide numbering reflects cDNA numbering with +1 corresponding to the A of the ATG tanslation initiation codon in the reference sequence.

Supp. Table S2A. NS-NS/LAH signature (125 probes, 124 genes)

Symbol	TargetID	Accession	L2R Disease/CTRL	ABS SNR Disease/CTRL	T-test p-value Disease vs Control
NCF1	ILMN_136961	NM_000265.1	1.321084166	0.554650871	0.00055653
ECGF1	ILMN_18760	NM_001953.2	1.139301768	0.56287464	0.00044382
CGI-38	ILMN_754	NM_015964.2	1.132573662	0.673556676	0.00002621
FGL2	ILMN_19861	NM_006682.1	1.070279843	0.57143162	0.00028665
RAPGEF1	ILMN_3184	NM_198679.1	0.98206944	0.561509994	0.00105147
GRN	ILMN_18655	NM_002087.2	0.964791405	0.568916029	0.00045024
SASH1	ILMN_22260	NM_015278.3	0.957600681	0.686162499	0.00002612
RP2	ILMN_28618	NM_006915.1	0.90480327	0.791543608	0.00000200
TUBB6	ILMN_26276	NM_032525.1	0.853006817	0.527954118	0.00057275
SLC25A24	ILMN_15753	NM_013386.2	0.837217818	0.860356608	0.00000062
MTMR11	ILMN_137079	NM_181873.1	0.808285699	0.655443175	0.00003297
PFC	ILMN_24742	NM_002621.1	0.791994208	0.504731573	0.00101265
CSF1R	ILMN_24980	NM_005211.2	0.766481286	0.566002268	0.00026247
CYBB	ILMN_13274	NM_000397.2	0.751730075	0.630439746	0.00010382
WARS	ILMN_25430	NM_213646.1	0.749824997	0.533953615	0.00061560
C9orf72	ILMN_9580	NM_145005.3	0.730448006	0.576732623	0.00021012
LOC339745	ILMN_21364	NM_001001664.1	0.725297986	0.847062003	0.00000035
MSR1	ILMN_16779	NM_002445.2	0.709933122	0.527357886	0.00089285
WDR33	ILMN_6704	NM_001006623.1	0.69789313	0.557560131	0.00076950
RB1	ILMN_4636	NM_000321.1	0.688171798	0.609960242	0.00009345
ADAM9	ILMN_922	NM_003816.2	0.673507261	0.559339882	0.00103667
P2RX1	ILMN_1134	NM_002558.2	0.667173036	0.582373311	0.00018210
TBC1D9	ILMN_25527	NM_015130.1	0.661083435	0.533359533	0.00052298
MCTP1	ILMN_2499	NM_001002796.1	0.657310261	0.552273564	0.00037623

VMO1	**ILMN_18899**	**NM_182566.1**	**0.643428254**	**0.539386685**	**0.00102405**
EMR1	**ILMN_12984**	**NM_001974.3**	**0.633183529**	**0.557693009**	**0.00036571**
PRDX3	**ILMN_13029**	**NM_014098.2**	**0.626217596**	**0.616369174**	**0.00020419**
MTSS1	**ILMN_7083**	**NM_014751.2**	**0.624826288**	**0.717204372**	**0.00000989**
PECAM1	**ILMN_7862**	**NM_000442.2**	**0.623438681**	**0.532080738**	**0.00072899**
SSFA2	**ILMN_4525**	**NM_006751.3**	**0.605800152**	**0.545007535**	**0.00050355**
CTDSP1	**ILMN_13739**	**NM_182642.1**	**0.60301904**	**0.540296413**	**0.00043850**
MLL3	**ILMN_24027**	**NM_021230.1**	**0.595752173**	**0.50418616**	**0.00094331**
ATP6V0A1	**ILMN_138088**	**NM_005177.2**	**0.56619738**	**0.618763559**	**0.00007647**
CST6	**ILMN_20022**	**NM_001323.2**	**0.565753462**	**0.534088911**	**0.00194061**
DPYSL2	**ILMN_9671**	**NM_001386.4**	**0.565577205**	**0.517590614**	**0.00081030**
LOC51136	**ILMN_27239**	**NM_016125.2**	**0.55785882**	**0.524110701**	**0.00070125**
SYK	**ILMN_8126**	**NM_003177.3**	**0.557306354**	**0.508150751**	**0.00100071**
RNPEP	**ILMN_22895**	**NM_020216.3**	**0.55610558**	**0.598623524**	**0.00016502**
ATP6V1A	**ILMN_30226**	**NM_001690.2**	**0.550949418**	**0.573954134**	**0.00026355**
MLL3	**ILMN_14020**	**NM_170606.1**	**0.549391752**	**0.548625483**	**0.00041986**
QKI	**ILMN_4124**	**NM_206854.1**	**0.545749725**	**0.53359302**	**0.00053097**
HLA-DMB	**ILMN_2252**	**NM_002118.3**	**0.541171517**	**0.741714584**	**0.00000443**
UTRN	**ILMN_15375**	**NM_007124.1**	**0.527936565**	**0.626744433**	**0.00008486**
AGPAT2	**ILMN_6967**	**NM_006412.3**	**0.526089017**	**0.537277823**	**0.00107744**
SLC36A1	**ILMN_22384**	**NM_078483.2**	**0.525363533**	**0.579074576**	**0.00018789**
WDFY2	**ILMN_14243**	**NM_052950.2**	**0.51738607**	**0.949471314**	**0.00000014**
SNX2	**ILMN_4948**	**NM_003100.2**	**0.511419645**	**0.645587156**	**0.00004356**
POGZ	**ILMN_14081**	**NM_145796.2**	**0.509196883**	**0.614652705**	**0.00034779**
CBR3	**ILMN_8256**	**NM_001236.3**	**-0.501548372**	**0.576774225**	**0.00031293**
ACOT4	**ILMN_5812**	**NM_152331.2**	**-0.506300623**	**0.501282601**	**0.00145993**
ZNF545	**ILMN_15084**	**NM_133466.1**	**-0.50676991**	**0.666747897**	**0.00004604**
ARHGAP10	**ILMN_24582**	**NM_024605.3**	**-0.507674999**	**0.658071995**	**0.00003942**
ATXN7L2	**ILMN_6796**	**NM_153340.2**	**-0.509429145**	**0.506309516**	**0.00116308**

PIP5K2A	**ILMN_6957**	**NM_005028.3**	**-0.511620973**	**0.766986504**	**0.00000303**
BRF2	**ILMN_2966**	**NM_018310.2**	**-0.513168419**	**0.573735878**	**0.00022956**
LAX1	**ILMN_29004**	**NM_017773.2**	**-0.520792925**	**0.715075083**	**0.00001090**
PTGER4	**ILMN_13328**	**NM_000958.2**	**-0.532099462**	**0.562488851**	**0.00039290**
C21orf57	**ILMN_21121**	**NM_058181.1**	**-0.532614088**	**0.557683419**	**0.00039802**
CCDC58	**ILMN_27140**	**NM_001017928.2**	**-0.54006179**	**0.626422203**	**0.00008076**
ALDOC	**ILMN_15767**	**NM_005165.2**	**-0.551121983**	**0.599294042**	**0.00013341**
SDCCAG33	**ILMN_138968**	**NM_005786.3**	**-0.55159881**	**0.635549907**	**0.00012881**
FAM84B	**ILMN_7137**	**NM_174911.3**	**-0.555973314**	**0.66943369**	**0.00004156**
SLC25A23	**ILMN_5925**	**NM_024103.2**	**-0.564123021**	**0.656348275**	**0.00011161**
NPDC1	**ILMN_5190**	**NM_015392.2**	**-0.566353596**	**0.51503047**	**0.00078580**
FYN	**ILMN_5919**	**NM_002037.3**	**-0.570318002**	**0.644773866**	**0.00004925**
RHOF	**ILMN_1762**	**NM_019034.2**	**-0.588562031**	**0.512472435**	**0.00087113**
LCMT2	**ILMN_7283**	**NM_014793.3**	**-0.59408272**	**0.676237692**	**0.00004917**
LPHN1	**ILMN_12017**	**NM_014921.3**	**-0.595977927**	**0.555516456**	**0.00060169**
ACACB	**ILMN_3318**	**NM_001093.2**	**-0.60148083**	**0.647708036**	**0.00004546**
PIM2	**ILMN_27932**	**NM_006875.2**	**-0.606699437**	**0.745720347**	**0.00000688**
RASA3	**ILMN_19501**	**NM_007368.2**	**-0.609728339**	**0.51866888**	**0.00084582**
CD2	**ILMN_6738**	**NM_001767.2**	**-0.613600011**	**0.646691342**	**0.00006305**
MGC17330	**ILMN_15026**	**NM_052880.3**	**-0.615102387**	**0.554114679**	**0.00124969**
PITPNC1	**ILMN_12660**	**NM_181671.1**	**-0.617188651**	**0.763012992**	**0.00000348**
IL18RAP	**ILMN_16084**	**NM_003853.2**	**-0.618785163**	**0.595867225**	**0.00013053**
ABHD14B	**ILMN_24812**	**NM_032750.1**	**-0.620384302**	**0.753596144**	**0.00000525**
TNFSF9	**ILMN_18030**	**NM_003811.2**	**-0.63645865**	**0.504502625**	**0.00123920**
GFI1	**ILMN_30079**	**NM_005263.2**	**-0.638218337**	**0.810063077**	**0.00000130**
ZBTB16	**ILMN_11903**	**NM_006006.4**	**-0.640383593**	**0.523059876**	**0.00068922**
STAT4	**ILMN_8937**	**NM_003151.2**	**-0.642277435**	**0.741700081**	**0.00000514**
SMPD1	**ILMN_8257**	**NM_001007593.1**	**-0.648648924**	**0.538651833**	**0.00047990**
SFXN1	**ILMN_16398**	**NM_022754.4**	**-0.654170933**	**0.523626325**	**0.00080819**

TCEA3	ILMN_27218	NM_003196.1	-0.657704996	0.592066255	0.00024669
CD8A	ILMN_2358	NM_001768.4	-0.660860793	0.594696025	0.00014620
PRR7	ILMN_17178	NM_030567.2	-0.665158852	0.536832382	0.00057470
C10orf35	ILMN_13661	NM_145306.1	-0.669357423	0.58713647	0.00016046
PLCG1	ILMN_3773	NM_002660.2	-0.669392435	0.584297133	0.00019981
PAFAH2	ILMN_3410	NM_000437.2	-0.684827426	0.892782604	0.00000055
CACNA2D2	ILMN_15989	NM_001005505.1	-0.686912394	0.613426228	0.00010529
ITK	ILMN_23317	NM_005546.3	-0.690033069	0.704824671	0.00002724
MGAT4A	ILMN_139237	NM_012214.1	-0.701476401	0.588282515	0.00018484
TGFBR3	ILMN_22620	NM_003243.2	-0.708248557	0.697370522	0.00002515
CD3E	ILMN_24240	NM_000733.2	-0.711671939	0.629082883	0.00008905
TKTL1	ILMN_23315	NM_012253.2	-0.727727687	0.605461651	0.00018975
HOXB2	ILMN_137588	NM_002145.2	-0.739430194	0.551939309	0.00033964
ICOS	ILMN_9996	NM_012092.2	-0.745024028	0.513242042	0.00083515
CTLA4	ILMN_138103	NM_005214.2	-0.750478694	0.934673244	0.00000008
PTPRM	ILMN_19957	NM_002845.2	-0.752842383	0.636816195	0.00006099
FHIT	ILMN_15082	NM_002012.1	-0.766674862	0.578811154	0.00046674
TSEN54	ILMN_8569	NM_207346.1	-0.776875745	0.599435131	0.00011930
PDZD4	ILMN_3986	NM_032512.2	-0.777079223	0.603890033	0.00011783
ELA1	ILMN_24603	NM_001971.4	-0.778277484	0.725031145	0.00000665
CD5	ILMN_29547	NM_014207.2	-0.780011452	0.501385904	0.00123187
FLT3LG	ILMN_4754	NM_001459.2	-0.786977344	0.672271482	0.00002435
PPP2R2B	ILMN_1007	NM_181676.1	-0.790685672	0.615737601	0.00012564
TARP	ILMN_25716	NM_001003799.1	-0.793277751	0.64274719	0.00006665
BCL9L	ILMN_27555	NM_182557.1	-0.808789234	0.554059637	0.00032290
SOCS1	ILMN_3038	NM_003745.1	-0.825203535	0.502522482	0.00114042
CCDC65	ILMN_22276	NM_033124.2	-0.826315503	0.575384317	0.00020769
BAMBI	ILMN_8469	NM_012342.2	-0.828451265	0.502263163	0.00097632
PLEKHB1	ILMN_2859	NM_021200.1	-0.840020952	0.839629344	0.00000093

C6orf105	**ILMN_10287**	**NM_032744.1**	**-0.840919329**	**0.664422571**	**0.00003018**
KSP37	**ILMN_16684**	**NM_031950.2**	**-0.842708345**	**0.681807509**	**0.00002272**
GZMH	**ILMN_10239**	**NM_033423.2**	**-0.845233571**	**0.527411198**	**0.00086217**
ADRB2	**ILMN_2022**	**NM_000024.3**	**-0.86626972**	**0.847589262**	**0.00000084**
MAL	**ILMN_3411**	**NM_002371.2**	**-0.877719549**	**0.925808311**	**0.00000027**
CITED4	**ILMN_15271**	**NM_133467.2**	**-0.880279922**	**0.765096915**	**0.00000907**
PTGDS	**ILMN_19248**	**NM_000954.5**	**-0.885287037**	**0.505533305**	**0.00172082**
AKR1C3	**ILMN_11871**	**NM_003739.4**	**-0.93749951**	**0.627865296**	**0.00008072**
H2AFX	**ILMN_23585**	**NM_002105.2**	**-0.942415472**	**0.813438146**	**0.00000080**
FEZ1	**ILMN_28992**	**NM_005103.3**	**-1.016084362**	**0.763414541**	**0.00000304**
ZNF683	**ILMN_2307**	**NM_173574.1**	**-1.017805442**	**0.562264358**	**0.00032947**
IL23A	**ILMN_20797**	**NM_016584.2**	**-1.054966924**	**0.588923763**	**0.00017724**
GNLY	**ILMN_13145**	**NM_012483.1**	**-1.061532661**	**0.821905939**	**0.00000159**
BATF	**ILMN_3307**	**NM_006399.2**	**-1.12180425**	**0.519569456**	**0.00075257**

**Supp. Table S2B. *PTPN11* signature (225 probes, 223 genes)**					
**Symbol**	**TargetID**	**Accession**	**L2R Disease/CTRL**	**ABS SNR Disease/CTRL**	**T-test p-value Disease vs Control**
HCA112	**ILMN_24990**	**NM_018487.2**	**1.640572025**	**0.46899836**	**0.00045873**
EMP1	**ILMN_25097**	**NM_001423.1**	**1.573890651**	**0.30682888**	**0.00314480**
TNFRSF12A	**ILMN_11654**	**NM_016639.1**	**1.471344009**	**0.327517025**	**0.00230098**
RAPGEF1	**ILMN_3184**	**NM_198679.1**	**1.350848899**	**0.561509994**	**0.00002477**
CGI-38	**ILMN_754**	**NM_015964.2**	**1.26429885**	**0.673556676**	**0.00001957**
EGR2	**ILMN_10721**	**NM_000399.2**	**1.25189554**	**0.216246616**	**0.00078760**
ECGF1	**ILMN_18760**	**NM_001953.2**	**1.227244131**	**0.56287464**	**0.00025184**
VASH1	**ILMN_4905**	**NM_014909.2**	**1.187049881**	**0.436937865**	**0.00009345**
NCF1	**ILMN_136961**	**NM_000265.1**	**1.183170469**	**0.554650871**	**0.00215972**
C1QA	**ILMN_138176**	**NM_015991.1**	**1.174230265**	**0.460228509**	**0.00038154**
LILRA5	**ILMN_13562**	**NM_181986.1**	**1.157891702**	**0.495486116**	**0.00025816**
FGL2	**ILMN_19861**	**NM_006682.1**	**1.117058744**	**0.57143162**	**0.00016718**
HBEGF	**ILMN_13020**	**NM_001945.1**	**1.103141795**	**0.251961923**	**0.00140005**
OSCAR	**ILMN_3784**	**NM_130771.2**	**1.064976797**	**0.450026773**	**0.00017363**
PTRF	**ILMN_22301**	**NM_012232.2**	**1.049816639**	**0.426525135**	**0.00301579**
CD300LB	**ILMN_22665**	**NM_174892.1**	**1.029237831**	**0.417482967**	**0.00014046**
NP	**ILMN_18063**	**NM_000270.1**	**1.004914876**	**0.450327209**	**0.00137933**
BTBD14A	**ILMN_10561**	**NM_144653.3**	**0.977900069**	**0.497255735**	**0.00000093**
SERPINE1	**ILMN_6244**	**NM_000602.1**	**0.976345257**	**0.398119074**	**0.00471708**
GRN	**ILMN_18655**	**NM_002087.2**	**0.971879492**	**0.568916029**	**0.00009629**
SASH1	**ILMN_22260**	**NM_015278.3**	**0.968150053**	**0.686162499**	**0.00002487**
TUBB6	**ILMN_26276**	**NM_032525.1**	**0.957340349**	**0.527954118**	**0.00134300**

WARS	**ILMN_25430**	**NM_213646.1**	**0.928591613**	**0.533953615**	**0.00019458**
RP2	**ILMN_28618**	**NM_006915.1**	**0.917224311**	**0.791543608**	**0.00000945**
C9orf72	**ILMN_9580**	**NM_145005.3**	**0.913617277**	**0.576732623**	**0.00002725**
ALDH3B1	**ILMN_26797**	**NM_001030010.1**	**0.901692076**	**0.482841759**	**0.00018900**
ADAM9	**ILMN_922**	**NM_003816.2**	**0.893136784**	**0.559339882**	**0.00015938**
LOC401233	**ILMN_17894**	**NM_001013680.1**	**0.892889063**	**0.394707704**	**0.00145884**
MTMR11	**ILMN_137079**	**NM_181873.1**	**0.883969064**	**0.655443175**	**0.00006313**
MSR1	**ILMN_16779**	**NM_002445.2**	**0.883563599**	**0.527357886**	**0.00094744**
CSF1R	**ILMN_24980**	**NM_005211.2**	**0.864191294**	**0.566002268**	**0.00038381**
PFC	**ILMN_24742**	**NM_002621.1**	**0.845059664**	**0.504731573**	**0.00056782**
UNQ3033	**ILMN_29051**	**NM_198481.1**	**0.837564175**	**0.313324062**	**0.00308231**
CYBB	**ILMN_13274**	**NM_000397.2**	**0.832261527**	**0.630439746**	**0.00001732**
P2RX1	**ILMN_1134**	**NM_002558.2**	**0.830035069**	**0.582373311**	**0.00009607**
WDR33	**ILMN_6704**	**NM_001006623.1**	**0.816852502**	**0.557560131**	**0.00038434**
CST6	**ILMN_20022**	**NM_001323.2**	**0.807324519**	**0.534088911**	**0.00007649**
ZNF503	**ILMN_2048**	**NM_032772.3**	**0.805381467**	**0.432169646**	**0.00091319**
LIMS1	**ILMN_11207**	**NM_004987.3**	**0.800923571**	**0.336151079**	**0.00198880**
VMO1	**ILMN_18899**	**NM_182566.1**	**0.785342972**	**0.539386685**	**0.00027633**
ANKDD1A	**ILMN_6117**	**NM_182703.2**	**0.781657454**	**0.356691847**	**0.00079619**
CAMK1	**ILMN_21373**	**NM_003656.3**	**0.759144587**	**0.492115675**	**0.00061577**
APLP2	**ILMN_19935**	**NM_001642.1**	**0.752003991**	**0.455656335**	**0.00215605**
ZDHHC1	**ILMN_29630**	**NM_013304.1**	**0.751814563**	**0.488268233**	**0.00134316**
SLC25A24	**ILMN_15753**	**NM_013386.2**	**0.74510769**	**0.860356608**	**0.00000472**
IL1RAP	**ILMN_8626**	**NM_134470.2**	**0.728393523**	**0.398730293**	**0.00112381**
IGSF6	**ILMN_7979**	**NM_005849.1**	**0.722354415**	**0.404621481**	**0.00184215**
MLL3	**ILMN_24027**	**NM_021230.1**	**0.707646583**	**0.50418616**	**0.00089322**
SSFA2	**ILMN_4525**	**NM_006751.3**	**0.704568057**	**0.545007535**	**0.00009856**
LOC339745	**ILMN_21364**	**NM_001001664.1**	**0.700391983**	**0.847062003**	**0.00001529**
QKI	**ILMN_4124**	**NM_206854.1**	**0.691646151**	**0.53359302**	**0.00016360**

PLEKHQ1	ILMN_25713	NM_025201.3	0.68445947	0.381013076	0.00203275
TGM2	ILMN_8134	NM_198951.1	0.682066115	0.417835214	0.00021976
ARRDC4	ILMN_11014	NM_183376.1	0.672533706	0.417237908	0.00456098
FGR	ILMN_21930	NM_005248.1	0.669375621	0.466533807	0.00161083
RGS12	ILMN_15717	NM_002926.2	0.667379422	0.456153285	0.00063938
RNPEP	ILMN_22895	NM_020216.3	0.666137061	0.598623524	0.00000576
SH2D3C	ILMN_8703	NM_170600.1	0.665808955	0.403944385	0.00567878
SYK	ILMN_8126	NM_003177.3	0.6626873	0.508150751	0.00016720
ATP6V0A1	ILMN_138088	NM_005177.2	0.652003802	0.618763559	0.00017335
KIAA0513	ILMN_21473	NM_014732.2	0.646910539	0.482625046	0.00007375
POGZ	ILMN_14081	NM_145796.2	0.638240534	0.614652705	0.00002428
MCTP1	ILMN_2499	NM_001002796.1	0.631772234	0.552273564	0.00088773
GBGT1	ILMN_18833	NM_021996.3	0.621287266	0.423734301	0.00332459
EMR1	ILMN_12984	NM_001974.3	0.619543816	0.557693009	0.00157518
CTSS	ILMN_13149	NM_004079.3	0.61862623	0.483715093	0.00125229
RB1	ILMN_4636	NM_000321.1	0.613674119	0.609960242	0.00277011
DAPK1	ILMN_138506	NM_004938.1	0.611697486	0.442825305	0.00275467
PECAM1	ILMN_7862	NM_000442.2	0.60917983	0.532080738	0.00082461
DPYSL2	ILMN_9671	NM_001386.4	0.598054115	0.517590614	0.00082555
BCL2L2	ILMN_9171	NM_004050.2	0.595693337	0.618322489	0.00002477
NAPSA	ILMN_12115	NM_004851.1	0.589618584	0.280978366	0.00392265
PFKFB2	ILMN_12195	NM_006212.2	0.58781744	0.527917721	0.00022229
AP1S2	ILMN_3812	NM_003916.3	0.586783759	0.488969433	0.00197668
SIPA1L1	ILMN_3802	NM_015556.1	0.586288021	0.240962801	0.00379876
HSBP1	ILMN_3025	NM_001537.2	0.583504819	1.028093735	0.00000000
GM2A	ILMN_8836	NM_000405.3	0.581321147	0.336407175	0.00381796
MLL3	ILMN_14020	NM_170606.1	0.581109919	0.548625483	0.00087683
PARP14	ILMN_28517	NM_017554.1	0.576341503	0.437357265	0.00125215
SLC36A1	ILMN_22384	NM_078483.2	0.569195789	0.579074576	0.00078930

IL15	**ILMN_16803**	**NM_172174.1**	**0.564822943**	**0.431587521**	**0.00418995**
CTDSP1	**ILMN_13739**	**NM_182642.1**	**0.561848041**	**0.540296413**	**0.00535669**
ATP6V1A	**ILMN_30226**	**NM_001690.2**	**0.55307366**	**0.573954134**	**0.00042831**
MGC33692	**ILMN_17332**	**NM_001001794.1**	**0.551155723**	**0.452553135**	**0.00013551**
PRDX3	**ILMN_13029**	**NM_014098.2**	**0.549182004**	**0.616369174**	**0.00026009**
PHCA	**ILMN_3836**	**NM_018367.3**	**0.545298912**	**0.513985519**	**0.00048863**
GNS	**ILMN_6937**	**NM_002076.2**	**0.54005597**	**0.702188774**	**0.00000296**
UTRN	**ILMN_15375**	**NM_007124.1**	**0.537147127**	**0.626744433**	**0.00057966**
FAM65A	**ILMN_17641**	**NM_024519.2**	**0.533570053**	**0.520527473**	**0.00007999**
MTSS1	**ILMN_7083**	**NM_014751.2**	**0.528607408**	**0.717204372**	**0.00086115**
WDR41	**ILMN_28575**	**NM_018268.2**	**0.526931841**	**0.420502405**	**0.00262646**
PSRC2	**ILMN_139272**	**NM_144982.3**	**0.522449121**	**0.532229639**	**0.00143090**
WDFY2	**ILMN_14243**	**NM_052950.2**	**0.522160857**	**0.949471314**	**0.00000051**
HLA-DMB	**ILMN_2252**	**NM_002118.3**	**0.521835548**	**0.741714584**	**0.00015061**
LYL1	**ILMN_18317**	**NM_005583.3**	**0.510433643**	**0.450602912**	**0.00217954**
BCKDK	**ILMN_13829**	**NM_005881.1**	**0.506453376**	**0.42032352**	**0.00081655**
RAPH1	**ILMN_7647**	**NM_025252.3**	**0.501989973**	**0.498454201**	**0.00010081**
STAT5A	**ILMN_10520**	**NM_003152.2**	**-0.500670735**	**0.435370699**	**0.00157763**
PHF20	**ILMN_20893**	**NM_016436.3**	**-0.503987875**	**0.498824041**	**0.00341753**
GALM	**ILMN_10698**	**NM_138801.1**	**-0.504181622**	**0.359664965**	**0.00069713**
F8A1	**ILMN_23293**	**NM_012151.3**	**-0.507798082**	**0.491539841**	**0.00083689**
NCK2	**ILMN_8369**	**NM_003581.2**	**-0.51108628**	**0.586738229**	**0.00008988**
NUCB2	**ILMN_23510**	**NM_005013.1**	**-0.511382323**	**0.40932896**	**0.00212353**
C21orf57	**ILMN_21121**	**NM_058181.1**	**-0.512831996**	**0.557683419**	**0.00314669**
TERF2	**ILMN_21134**	**NM_005652.2**	**-0.513847741**	**0.330032666**	**0.00011668**
ALDOC	**ILMN_15767**	**NM_005165.2**	**-0.516607321**	**0.599294042**	**0.00077976**
PIP5K2A	**ILMN_6957**	**NM_005028.3**	**-0.517743119**	**0.766986504**	**0.00000226**
CCNB1IP1	**ILMN_16420**	**NM_021178.2**	**-0.518250647**	**0.8164184**	**0.00000075**
MLLT11	**ILMN_21397**	**NM_006818.3**	**-0.518730151**	**0.456654026**	**0.00009982**

DNAJB9	ILMN_14354	NM_012328.1	-0.520897603	0.409938084	0.00286409
IL32	ILMN_3781	NM_001012632.1	-0.522542869	0.44597114	0.00295954
C10orf58	ILMN_17906	NM_032333.2	-0.529379401	0.490332648	0.00003745
ACTR8	ILMN_13548	NM_022899.3	-0.529602637	0.57196257	0.00006576
FTS	ILMN_9667	NM_022476.2	-0.537860477	0.34369181	0.00399328
C10orf104	ILMN_7723	NM_173473.2	-0.538052773	0.406663671	0.00136886
FLJ38451	ILMN_19229	NM_175872.3	-0.538937665	0.495254186	0.00114409
ZNF545	ILMN_15084	NM_133466.1	-0.550666864	0.666747897	0.00002844
FYN	ILMN_5919	NM_002037.3	-0.551294472	0.644773866	0.00023300
C9orf30	ILMN_15668	NM_080655.1	-0.551461916	0.564346926	0.00012317
PTPN4	ILMN_29562	NM_002830.2	-0.552003607	0.518687009	0.00005066
LOC81558	ILMN_29450	NM_030802.2	-0.555552873	0.386435279	0.00176926
ARHGAP10	ILMN_24582	NM_024605.3	-0.555899324	0.658071995	0.00024664
RNF144	ILMN_15740	NM_014746.2	-0.558532274	0.398131551	0.00229456
PRAF1	ILMN_2978	NM_022490.1	-0.558972433	0.701645712	0.00001024
NDFIP2	ILMN_23410	NM_019080.1	-0.564231612	0.273391931	0.00514154
HAPLN3	ILMN_19816	NM_178232.2	-0.565399962	0.479939961	0.00441729
CCDC58	ILMN_27140	NM_001017928.2	-0.568494148	0.626422203	0.00010018
TPST2	ILMN_13248	NM_001008566.1	-0.568686202	0.407049282	0.00051135
RAB7L1	ILMN_27192	NM_003929.1	-0.576039588	0.826731839	0.00000008
LAX1	ILMN_29004	NM_017773.2	-0.576809737	0.715075083	0.00000556
TUBE1	ILMN_7563	NM_016262.3	-0.579367228	0.372958665	0.00033726
SFXN1	ILMN_16398	NM_022754.4	-0.58091346	0.523626325	0.00185844
ACACB	ILMN_3318	NM_001093.2	-0.582496141	0.647708036	0.00095975
TBCC	ILMN_25053	NM_003192.1	-0.585662135	0.47986098	0.00022676
CBR3	ILMN_8256	NM_001236.3	-0.587778543	0.576774225	0.00006243
SATB1	ILMN_6836	NM_002971.2	-0.59024576	0.491733379	0.00029065
MGC17330	ILMN_15026	NM_052880.3	-0.592236188	0.554114679	0.00105096
VIPR1	ILMN_27565	NM_004624.2	-0.593070326	0.451776741	0.00062580

NPDC1	**ILMN_5190**	**NM_015392.2**	**-0.593493903**	**0.51503047**	**0.00186892**
AGMAT	**ILMN_24467**	**NM_024758.3**	**-0.595396271**	**0.460747866**	**0.00275354**
ZNF184	**ILMN_22535**	**NM_007149.1**	**-0.597689887**	**0.368618408**	**0.00486717**
PRR7	**ILMN_17178**	**NM_030567.2**	**-0.604413556**	**0.536832382**	**0.00286203**
SMPD1	**ILMN_8257**	**NM_001007593.1**	**-0.604949755**	**0.538651833**	**0.00161182**
BRF2	**ILMN_2966**	**NM_018310.2**	**-0.606067504**	**0.573735878**	**0.00010726**
PTGER4	**ILMN_13328**	**NM_000958.2**	**-0.606450332**	**0.562488851**	**0.00001934**
FEZ1	**ILMN_419**	**NM_022549.2**	**-0.609292102**	**0.457577683**	**0.00682154**
SDCCAG33	**ILMN_138968**	**NM_005786.3**	**-0.614368954**	**0.635549907**	**0.00001713**
DPP4	**ILMN_20248**	**NM_001935.3**	**-0.618288342**	**0.45091084**	**0.00176525**
PNRC1	**ILMN_24710**	**NM_006813.1**	**-0.628831373**	**0.489596307**	**0.00111420**
FLJ20152	**ILMN_139301**	**NM_019000.2**	**-0.634230056**	**0.456511434**	**0.00109561**
MAN1C1	**ILMN_12633**	**NM_020379.1**	**-0.635341768**	**0.48916714**	**0.00048381**
SOX8	**ILMN_30180**	**NM_014587.2**	**-0.638937302**	**0.457483381**	**0.00089229**
CACNA2D2	**ILMN_15989**	**NM_001005505.1**	**-0.647755763**	**0.613426228**	**0.00134567**
DNAJB1	**ILMN_19740**	**NM_006145.1**	**-0.647877454**	**0.474267591**	**0.00101160**
TXK	**ILMN_19719**	**NM_003328.1**	**-0.649402059**	**0.355423427**	**0.00273011**
PKIA	**ILMN_4343**	**NM_006823.2**	**-0.651907566**	**0.461906271**	**0.00001972**
ZBTB16	**ILMN_11903**	**NM_006006.4**	**-0.654391514**	**0.523059876**	**0.00196632**
HSPA8	**ILMN_14829**	**NM_153201.1**	**-0.659647107**	**0.627352179**	**0.00000291**
LEF1	**ILMN_30265**	**NM_016269.2**	**-0.664492864**	**0.374513301**	**0.00142701**
ABHD14B	**ILMN_24812**	**NM_032750.1**	**-0.667660274**	**0.753596144**	**0.00000440**
RPL14	**ILMN_138835**	**NM_003973.2**	**-0.671818838**	**0.42344653**	**0.00348419**
PITPNC1	**ILMN_12660**	**NM_181671.1**	**-0.675741411**	**0.763012992**	**0.00000200**
LPHN1	**ILMN_12017**	**NM_014921.3**	**-0.680224127**	**0.555516456**	**0.00003588**
MGAT4A	**ILMN_139237**	**NM_012214.1**	**-0.686439815**	**0.588282515**	**0.00020394**
GIMAP7	**ILMN_2211**	**NM_153236.3**	**-0.688384375**	**0.309418302**	**0.00108814**
GFI1	**ILMN_30079**	**NM_005263.2**	**-0.688429664**	**0.810063077**	**0.00000287**
STAT4	**ILMN_8937**	**NM_003151.2**	**-0.69335148**	**0.741700081**	**0.00001454**

TGFBR3	ILMN_22620	NM_003243.2	-0.695968502	0.697370522	0.00024601
NAP1L5	ILMN_5355	NM_153757.1	-0.696281078	0.482970013	0.00118408
TRAT1	ILMN_24557	NM_016388.2	-0.701865172	0.431978638	0.00022583
PIM2	ILMN_27932	NM_006875.2	-0.705939469	0.745720347	0.00000049
MGC15763	ILMN_137229	NM_138381.1	-0.707467198	0.452639641	0.00160886
LCMT2	ILMN_7283	NM_014793.3	-0.708548025	0.676237692	0.00000198
PLCG1	ILMN_3773	NM_002660.2	-0.713682121	0.584297133	0.00056091
IL18RAP	ILMN_16084	NM_003853.2	-0.715943068	0.595867225	0.00016263
CD5	ILMN_29547	NM_014207.2	-0.719666211	0.501385904	0.00212931
FAM84B	ILMN_7137	NM_174911.3	-0.72095523	0.66943369	0.00000022
NCR3	ILMN_19702	NM_147130.1	-0.724076246	0.486364877	0.00400738
TSEN54	ILMN_8569	NM_207346.1	-0.727498633	0.599435131	0.00170913
FLT3LG	ILMN_4754	NM_001459.2	-0.727594431	0.672271482	0.00038973
TKTL1	ILMN_23315	NM_012253.2	-0.727847582	0.605461651	0.00069483
CTLA4	ILMN_138103	NM_005214.2	-0.738130072	0.934673244	0.00000139
SOCS1	ILMN_3038	NM_003745.1	-0.739455522	0.502522482	0.00313209
ELA1	ILMN_24603	NM_001971.4	-0.744576877	0.725031145	0.00029205
CD8A	ILMN_2358	NM_001768.4	-0.744678001	0.594696025	0.00026942
ITK	ILMN_23317	NM_005546.3	-0.745007112	0.704824671	0.00008908
TCEA3	ILMN_27218	NM_003196.1	-0.750750252	0.592066255	0.00023655
ICOS	ILMN_9996	NM_012092.2	-0.75237042	0.513242042	0.00046944
KLRG1	ILMN_12613	NM_005810.3	-0.774039085	0.254607132	0.00294923
AXIN2	ILMN_26857	NM_004655.2	-0.805082477	0.436443044	0.00158082
BCL9L	ILMN_27555	NM_182557.1	-0.807656854	0.554059637	0.00141276
CD248	ILMN_25766	NM_020404.2	-0.812370151	0.463357687	0.00238338
FHIT	ILMN_15082	NM_002012.1	-0.820857634	0.578811154	0.00019084
C10orf35	ILMN_13661	NM_145306.1	-0.828257411	0.58713647	0.00010833
GPR171	ILMN_21194	NM_013308.2	-0.832290597	0.331305728	0.00290693
PTPRM	ILMN_19957	NM_002845.2	-0.832917155	0.636816195	0.00008494

CD2	**ILMN_6738**	**NM_001767.2**	**-0.840438326**	**0.646691342**	**0.00000117**
PDZD4	**ILMN_3986**	**NM_032512.2**	**-0.86138807**	**0.603890033**	**0.00013770**
BAMBI	**ILMN_8469**	**NM_012342.2**	**-0.871810097**	**0.502263163**	**0.00294778**
PLEKHB1	**ILMN_2859**	**NM_021200.1**	**-0.878172562**	**0.839629344**	**0.00000692**
HOXB2	**ILMN_137588**	**NM_002145.2**	**-0.884877446**	**0.551939309**	**0.00030257**
PAFAH2	**ILMN_3410**	**NM_000437.2**	**-0.8943644**	**0.892782604**	**0.00000000**
MAL	**ILMN_3411**	**NM_002371.2**	**-0.895921565**	**0.925808311**	**0.00000271**
SERPINE2	**ILMN_1946**	**NM_006216.2**	**-0.904483902**	**0.45419718**	**0.00008303**
C6orf105	**ILMN_10287**	**NM_032744.1**	**-0.909531884**	**0.664422571**	**0.00002912**
CD3E	**ILMN_24240**	**NM_000733.2**	**-0.93235239**	**0.629082883**	**0.00000614**
CD69	**ILMN_13491**	**NM_001781.1**	**-0.942527846**	**0.457624176**	**0.00064713**
PTGDS	**ILMN_19248**	**NM_000954.5**	**-0.946494867**	**0.505533305**	**0.00175037**
GIMAP5	**ILMN_27010**	**NM_018384.3**	**-0.953022963**	**0.419538221**	**0.00201391**
HSPA1B	**ILMN_25549**	**NM_005346.3**	**-0.953328978**	**0.212241703**	**0.00269290**
PPP2R2B	**ILMN_1007**	**NM_181676.1**	**-0.965173716**	**0.615737601**	**0.00006002**
EOMES	**ILMN_19998**	**NM_005442.2**	**-0.968337892**	**0.470291119**	**0.00028010**
H2AFX	**ILMN_23585**	**NM_002105.2**	**-0.97692086**	**0.813438146**	**0.00000330**
GZMH	**ILMN_10239**	**NM_033423.2**	**-0.98083047**	**0.527411198**	**0.00060255**
CITED4	**ILMN_15271**	**NM_133467.2**	**-0.984893442**	**0.765096915**	**0.00000350**
CCDC65	**ILMN_22276**	**NM_033124.2**	**-0.990896676**	**0.575384317**	**0.00024815**
KSP37	**ILMN_16684**	**NM_031950.2**	**-0.999642264**	**0.681807509**	**0.00001688**
ZNF683	**ILMN_2307**	**NM_173574.1**	**-1.032728562**	**0.562264358**	**0.00193259**
TARP	**ILMN_25716**	**NM_001003799.1**	**-1.051625612**	**0.64274719**	**0.00000020**
ADRB2	**ILMN_2022**	**NM_000024.3**	**-1.055053118**	**0.847589262**	**0.00000011**
FEZ1	**ILMN_28992**	**NM_005103.3**	**-1.127136623**	**0.763414541**	**0.00001994**
GNLY	**ILMN_13145**	**NM_012483.1**	**-1.148412672**	**0.821905939**	**0.00000141**
BATF	**ILMN_3307**	**NM_006399.2**	**-1.174102973**	**0.519569456**	**0.00332655**
AKR1C3	**ILMN_11871**	**NM_003739.4**	**-1.189643568**	**0.627865296**	**0.00000337**
IL23A	**ILMN_20797**	**NM_016584.2**	**-1.232537149**	**0.588923763**	**0.00009415**

**Supp. Table S2C. *SOS1* signature (75 probes, 73 genes)**					
**Symbol**	**TargetID**	**Accession**	**L2R Disease/CTRL**	**ABS SNR Disease/CTRL**	**T-test p-value Disease vs Control**
RALGPS2	**ILMN_701**	**NM_018037.1**	**0.887552664**	**0.448616072**	**0.00161622**
PHF20L1	**ILMN_25591**	**NM_032205.2**	**0.886871298**	**0.381517999**	**0.00038379**
KLHL24	**ILMN_26914**	**NM_017644.3**	**0.789824755**	**0.505242446**	**0.00002312**
EIF2AK3	**ILMN_26832**	**NM_004836.3**	**0.785432084**	**0.046890124**	**0.00339153**
PTBP2	**ILMN_556**	**NM_021190.1**	**0.779793993**	**0.183024136**	**0.00664570**
MTMR6	**ILMN_29967**	**NM_004685.2**	**0.777000277**	**0.232481964**	**0.00177702**
ZNF529	**ILMN_24517**	**NM_020951.1**	**0.768697038**	**0.137110056**	**0.00000472**
CHD7	**ILMN_29669**	**NM_017780.2**	**0.763311195**	**0.053506036**	**0.00053082**
WDR33	**ILMN_6704**	**NM_001006623.1**	**0.762698764**	**0.557560131**	**0.00884851**
KIAA0776	**ILMN_26630**	**NM_015323.2**	**0.732036421**	**0.464464474**	**0.00038782**
SFRS12	**ILMN_8967**	**NM_139168.1**	**0.731518633**	**0.184442106**	**0.00041407**
RUNDC2A	**ILMN_705**	**NM_032167.1**	**0.721018309**	**0.284472895**	**0.00366201**
GABPA	**ILMN_28392**	**NM_002040.2**	**0.704789114**	**0.592919139**	**0.00187652**
RICTOR	**ILMN_2994**	**NM_152756.3**	**0.691392413**	**0.120805648**	**0.00227054**
MTSS1	**ILMN_7083**	**NM_014751.2**	**0.682974828**	**0.717204372**	**0.00112968**
IFT57	**ILMN_21390**	**NM_018010.2**	**0.675190082**	**0.229633029**	**0.00070011**
ADAM9	**ILMN_922**	**NM_003816.2**	**0.666180851**	**0.559339882**	**0.00378554**
FAM76B	**ILMN_22478**	**NM_144664.3**	**0.66576136**	**0.23771187**	**0.00010042**
N4BP2	**ILMN_7118**	**NM_018177.2**	**0.655425286**	**0.033654735**	**0.00172032**
RRM2B	**ILMN_11053**	**NM_015713.3**	**0.646273865**	**0.346860048**	**0.00848695**
PFKFB2	**ILMN_12195**	**NM_006212.2**	**0.617236289**	**0.527917721**	**0.00735058**
ZNF238	**ILMN_5128**	**NM_205768.1**	**0.610480963**	**0.060455926**	**0.00135583**
KLHL2	**ILMN_24870**	**NM_007246.2**	**0.604165553**	**0.60291658**	**0.00161532**

YTHDC2	**ILMN_23803**	**NM_022828.2**	**0.59555144**	**0.496064684**	**0.00614677**
POGZ	**ILMN_14081**	**NM_145796.2**	**0.583319511**	**0.614652705**	**0.00282129**
GMFB	**ILMN_14672**	**NM_004124.2**	**0.581168767**	**0.529244679**	**0.00157471**
OTUD6B	**ILMN_22397**	**NM_016023.2**	**0.56912336**	**0.304742464**	**0.00025795**
REV3L	**ILMN_17913**	**NM_002912.1**	**0.554148355**	**0.157091571**	**0.00791183**
APRIN	**ILMN_20213**	**NM_015032.1**	**0.554085012**	**0.273969544**	**0.00094423**
FBXL3	**ILMN_4998**	**NM_012158.1**	**0.551660242**	**0.218317691**	**0.00013307**
STIL	**ILMN_13235**	**NM_003035.1**	**0.545234399**	**0.287286475**	**0.00068769**
TMEM41B	**ILMN_8205**	**NM_015012.1**	**0.522333116**	**0.303516321**	**0.00269601**
CST6	**ILMN_20022**	**NM_001323.2**	**0.511932826**	**0.534088911**	**0.00921454**
GOLT1B	**ILMN_5124**	**NM_016072.2**	**0.510560422**	**0.202116925**	**0.00346801**
FUT8	**ILMN_9754**	**NM_178154.1**	**0.505064035**	**0.08981082**	**0.00698557**
NKTR	**ILMN_23378**	**NM_005385.3**	**0.50434282**	**0.294652457**	**0.00811464**
C6orf125	**ILMN_21424**	**NM_032340.1**	**-0.511492388**	**0.101057435**	**0.00762676**
MRPL34	**ILMN_5839**	**NM_023937.2**	**-0.511956713**	**0.348464173**	**0.00321254**
HSPA8	**ILMN_14829**	**NM_153201.1**	**-0.512721673**	**0.627352179**	**0.00056648**
CD2	**ILMN_6738**	**NM_001767.2**	**-0.522321422**	**0.646691342**	**0.00917697**
DOK2	**ILMN_137779**	**NM_201349.1**	**-0.523050722**	**0.190787501**	**0.00805286**
STIP1	**ILMN_28761**	**NM_006819.1**	**-0.525327079**	**0.124331572**	**0.00095539**
OAZ2	**ILMN_28141**	**NM_002537.1**	**-0.53824496**	**0.092826187**	**0.00356120**
LOC84661	**ILMN_18534**	**NM_032574.2**	**-0.548863028**	**0.216935995**	**0.00012449**
ACD	**ILMN_5907**	**NM_022914.1**	**-0.553579904**	**0.602229848**	**0.00137820**
LAX1	**ILMN_29004**	**NM_017773.2**	**-0.55474973**	**0.715075083**	**0.00188353**
IL27RA	**ILMN_9407**	**NM_004843.2**	**-0.562860221**	**0.10261292**	**0.00878351**
ARHGAP10	**ILMN_24582**	**NM_024605.3**	**-0.563466431**	**0.658071995**	**0.00152804**
TNFSF12	**ILMN_24599**	**NM_003809.2**	**-0.56770749**	**0.022913821**	**0.00272199**
PRUNE	**ILMN_27601**	**NM_021222.1**	**-0.586301157**	**0.552904262**	**0.00028104**
CTLA4	**ILMN_138103**	**NM_005214.2**	**-0.597555801**	**0.934673244**	**0.00243881**
LOC340061	**ILMN_18797**	**NM_198282.1**	**-0.598431803**	**0.147162724**	**0.00052177**

MAF	**ILMN_15072**	**NM_005360.3**	**-0.62329548**	**0.565299503**	**0.00302416**
PIP5K2A	**ILMN_6957**	**NM_005028.3**	**-0.62350074**	**0.766986504**	**0.00205711**
PSTPIP1	**ILMN_28119**	**NM_003978.2**	**-0.631863194**	**0.361537535**	**0.00139096**
ACACB	**ILMN_3318**	**NM_001093.2**	**-0.6348178**	**0.647708036**	**0.00745327**
TRAFD1	**ILMN_21539**	**NM_006700.1**	**-0.637847522**	**0.124969592**	**0.00050092**
F8A1	**ILMN_23293**	**NM_012151.3**	**-0.654659866**	**0.491539841**	**0.00294327**
CD300A	**ILMN_20704**	**NM_007261.2**	**-0.655556789**	**0.004475384**	**0.00771681**
GFI1	**ILMN_30079**	**NM_005263.2**	**-0.657538415**	**0.810063077**	**0.00106955**
DNAJB6	**ILMN_7651**	**NM_058246.3**	**-0.657540414**	**0.136315949**	**0.00094113**
CD244	**ILMN_7885**	**NM_016382.2**	**-0.678423036**	**0.064757497**	**0.00103571**
GALM	**ILMN_10698**	**NM_138801.1**	**-0.681834064**	**0.359664965**	**0.00008995**
TPST2	**ILMN_13248**	**NM_001008566.1**	**-0.769125088**	**0.407049282**	**0.00239127**
ASCL2	**ILMN_18051**	**NM_005170.2**	**-0.772775101**	**0.106077588**	**0.00417781**
GNLY	**ILMN_13145**	**NM_012483.1**	**-0.875999817**	**0.821905939**	**0.00579909**
LOC442535	**ILMN_16993**	**NM_001013738.1**	**-0.889737645**	**0.275652692**	**0.00467506**
GLRX	**ILMN_22606**	**NM_002064.1**	**-0.890483179**	**0.052715708**	**0.00059598**
APOBEC3G	**ILMN_15884**	**NM_021822.1**	**-0.893364866**	**0.380714828**	**0.00376115**
ADRB2	**ILMN_2022**	**NM_000024.3**	**-0.915472812**	**0.847589262**	**0.00010301**
ELA1	**ILMN_24603**	**NM_001971.4**	**-0.916899993**	**0.725031145**	**0.00159014**
TARP	**ILMN_25716**	**NM_001003799.1**	**-1.030821564**	**0.64274719**	**0.00032237**
EOMES	**ILMN_19998**	**NM_005442.2**	**-1.172206716**	**0.470291119**	**0.00070222**

**Supp. Table S2D. *SHOC2* signature (1407 probes, 1394 genes)**					
**Symbol**	**TargetID**	**Accession**	**L2R Disease/CTRL**	**ABS SNR Disease/CTRL**	**T-test p-value Disease vs Control**
P2RY13	**ILMN_9350**	**NM_176894.1**	**3.230795638**	**2.030205509**	**0.00001198**
NCF1	**ILMN_136961**	**NM_000265.1**	**3.004866306**	**2.360147597**	**0.00000080**
MS4A6A	**ILMN_2009**	**NM_022349.2**	**2.944993248**	**1.909673758**	**0.00004979**
PDK4	**ILMN_23211**	**NM_002612.2**	**2.917049119**	**1.36453749**	**0.00000847**
SLC25A20	**ILMN_29792**	**NM_000387.3**	**2.777594697**	**1.901681385**	**0.00000428**
TRIM5	**ILMN_760**	**NM_033034.1**	**2.561102611**	**1.686441216**	**0.00002655**
CD79B	**ILMN_139125**	**NM_000626.1**	**2.55830839**	**1.080894212**	**0.00048045**
TNFSF10	**ILMN_22827**	**NM_003810.2**	**2.430831207**	**1.68313466**	**0.00000525**
LTF	**ILMN_20816**	**NM_002343.2**	**2.415277282**	**1.137858007**	**0.00008957**
MARCH1	**ILMN_30212**	**NM_017923.2**	**2.400620779**	**1.559775716**	**0.00003376**
PSMB8	**ILMN_12139**	**NM_148919.3**	**2.372593273**	**1.806981162**	**0.00002239**
HSPA1A	**ILMN_6623**	**NM_005345.4**	**2.346566122**	**1.635865024**	**0.00041219**
FGL2	**ILMN_19861**	**NM_006682.1**	**2.268977886**	**1.920709435**	**0.00000936**
VNN1	**ILMN_14011**	**NM_004666.1**	**2.257784258**	**1.193513682**	**0.00016922**
ARHGAP30	**ILMN_15952**	**NM_001025598.1**	**2.255861969**	**1.586025349**	**0.00002175**
GCA	**ILMN_25626**	**NM_012198.2**	**2.197907204**	**1.703770495**	**0.00007464**
OAS1	**ILMN_2958**	**NM_016816.2**	**2.160455366**	**1.034754352**	**0.00009705**
SNAP23	**ILMN_29211**	**NM_003825.2**	**2.159463633**	**2.375349613**	**0.00000160**
NFE2	**ILMN_5749**	**NM_006163.1**	**2.125813345**	**1.404932233**	**0.00024891**
FLJ33641	**ILMN_2666**	**NM_152687.1**	**2.117646439**	**1.355499921**	**0.00005480**
CD1D	**ILMN_926**	**NM_001766.2**	**2.115842557**	**1.398556308**	**0.00035065**
CLEC12A	**ILMN_24114**	**NM_201625.1**	**2.106645106**	**1.864408376**	**0.00000426**
ECGF1	**ILMN_18760**	**NM_001953.2**	**2.044651326**	**1.166814773**	**0.00008155**

GRN	**ILMN_18655**	**NM_002087.2**	**2.03746384**	**1.669620931**	**0.00000234**
TLR5	**ILMN_18399**	**NM_003268.3**	**2.030218218**	**1.458223711**	**0.00023624**
C4orf18	**ILMN_657**	**NM_016613.4**	**2.002013059**	**1.060512331**	**0.00086947**
TSCOT	**ILMN_10869**	**NM_033051.2**	**1.977658192**	**1.470729682**	**0.00000801**
FZD2	**ILMN_12499**	**NM_001466.2**	**1.970187777**	**1.39558521**	**0.00004423**
AIM2	**ILMN_10231**	**NM_004833.1**	**1.952499983**	**1.452545483**	**0.00021779**
IDH1	**ILMN_14217**	**NM_005896.2**	**1.939463119**	**2.996203368**	**0.00000001**
CX3CR1	**ILMN_8593**	**NM_001337.3**	**1.935125349**	**1.317200409**	**0.00084901**
GIMAP4	**ILMN_26906**	**NM_018326.2**	**1.891877366**	**1.236698351**	**0.00013957**
SASH1	**ILMN_22260**	**NM_015278.3**	**1.869888573**	**1.605883404**	**0.00000252**
OPN3	**ILMN_26329**	**NM_001030012.1**	**1.869768692**	**1.815957077**	**0.00000269**
MS4A6A	**ILMN_1889**	**NM_152851.1**	**1.85318862**	**1.571238152**	**0.00006337**
EVA1	**ILMN_23335**	**NM_005797.2**	**1.845981029**	**1.278843117**	**0.00143771**
NYD-SP21	**ILMN_8529**	**NM_032597.2**	**1.83140234**	**1.405564479**	**0.00001489**
CACNA2D3	**ILMN_1908**	**NM_018398.2**	**1.829719711**	**1.168758985**	**0.00099030**
MS4A3	**ILMN_12025**	**NM_006138.4**	**1.81966477**	**1.178663506**	**0.00024345**
MOSC1	**ILMN_12517**	**NM_022746.2**	**1.80056635**	**1.08435644**	**0.00199068**
ARFIP1	**ILMN_15855**	**NM_014447.2**	**1.797399489**	**1.367232965**	**0.00009893**
RNASE6	**ILMN_14848**	**NM_005615.2**	**1.790511733**	**1.572275665**	**0.00000278**
C14orf106	**ILMN_5745**	**NM_018353.3**	**1.777748886**	**1.422897874**	**0.00001448**
CEBPA	**ILMN_27029**	**NM_004364.2**	**1.714680868**	**1.690296094**	**0.00004032**
GIYD2	**ILMN_25461**	**NM_178044.1**	**1.710230986**	**1.724065013**	**0.00000013**
SEPX1	**ILMN_7309**	**NM_016332.2**	**1.693931767**	**1.463290318**	**0.00024722**
MOSPD2	**ILMN_28307**	**NM_152581.1**	**1.686127391**	**1.195339716**	**0.00096150**
CPNE8	**ILMN_20089**	**NM_153634.2**	**1.676253065**	**1.272978087**	**0.00015784**
CGI-38	**ILMN_754**	**NM_015964.2**	**1.670439728**	**1.419306976**	**0.00022466**
FAM26B	**ILMN_19907**	**NM_015916.3**	**1.667037179**	**1.502766599**	**0.00009401**
CD1A	**ILMN_21799**	**NM_001763.1**	**1.654779216**	**1.541206072**	**0.00002028**
ASGR1	**ILMN_17580**	**NM_001671.2**	**1.642663186**	**1.127094292**	**0.00205181**
SLIC1	**ILMN_511**	**NM_182854.1**	**1.636866235**	**2.299482343**	**0.00000086**
RTN1	**ILMN_5569**	**NM_206857.1**	**1.630076214**	**0.889419362**	**0.00527996**

APAF1	ILMN_886	NM_181869.1	1.618807555	1.254949416	0.00092721
M6PR	ILMN_1682	NM_002355.2	1.618699365	1.266933529	0.00292618
CD33	ILMN_24441	NM_001772.2	1.617540554	1.279741937	0.00041582
SLC25A24	ILMN_15753	NM_013386.2	1.615632189	2.428121735	0.00000001
CDK5RAP3	ILMN_11403	NM_176095.1	1.615563142	1.296025136	0.00057345
C10orf26	ILMN_28562	NM_017787.3	1.595433956	1.558311538	0.00006121
FN5	ILMN_9654	NM_020179.1	1.593991929	1.803744426	0.00000975
CYSLTR1	ILMN_6966	NM_006639.2	1.584931332	1.485848853	0.00001350
GPR177	ILMN_2573	NM_001002292.1	1.562854266	1.082985034	0.00014765
NPL	ILMN_25291	NM_030769.1	1.562676534	1.43823841	0.00002591
VPS35	ILMN_21093	NM_018206.3	1.558138081	1.255233608	0.00041318
CARD9	ILMN_22365	NM_052813.2	1.55061094	0.937118385	0.00505249
CASP1	ILMN_10621	NM_033294.2	1.544866623	1.590869349	0.00000234
SAMD9L	ILMN_27133	NM_152703.2	1.536758823	1.039672893	0.00453992
PRDX3	ILMN_13029	NM_014098.2	1.530538083	2.621818437	0.00000001
LILRA5	ILMN_13562	NM_181986.1	1.528860757	0.798056866	0.00158893
OSCAR	ILMN_3784	NM_130771.2	1.518568683	1.005731609	0.00099353
PFC	ILMN_24742	NM_002621.1	1.501891159	1.020923486	0.00072327
PARP9	ILMN_12926	NM_031458.1	1.501029114	1.241277617	0.00003846
SNX27	ILMN_17828	NM_030918.5	1.498350472	1.290904726	0.00039031
PRAM1	ILMN_14804	NM_032152.3	1.495831227	0.983108985	0.00085923
STK3	ILMN_26935	NM_006281.1	1.487965795	1.169490975	0.00043491
AP1S2	ILMN_3812	NM_003916.3	1.481318788	1.497126236	0.00001992
LACTB2	ILMN_18082	NM_016027.1	1.478527427	0.919008169	0.00279394
TUBB	ILMN_23399	NM_178014.2	1.468843269	1.281248771	0.00012956
CAMK1	ILMN_21373	NM_003656.3	1.465942141	1.328031684	0.00004837
TLR10	ILMN_6615	NM_030956.2	1.465748435	0.652237676	0.00819716
RAB37	ILMN_8592	NM_001006638.1	1.459212098	1.176481542	0.00100970
CYBRD1	ILMN_4649	NM_024843.2	1.458125986	1.268588969	0.00052254
ANP32C	ILMN_11992	NM_012403.1	1.453611232	0.954665537	0.00066597
NAGK	ILMN_4544	NM_017567.2	1.451197882	1.58243748	0.00000213

TMEM55A	ILMN_7212	NM_018710.1	1.444584606	0.878598912	0.00771716
CEPT1	ILMN_14637	NM_006090.3	1.444163158	2.02435451	0.00000063
PECAM1	ILMN_7862	NM_000442.2	1.438004735	1.83143722	0.00000478
CD74	ILMN_21963	NM_001025159.1	1.434686147	0.972479846	0.00394973
LOC201895	ILMN_6140	NM_174921.1	1.431649983	1.617044642	0.00005280
WDR68	ILMN_17716	NM_005828.2	1.429422312	1.385745393	0.00028199
METTL3	ILMN_13907	NM_019852.2	1.42548153	0.91222508	0.00603552
LSM12	ILMN_1510	NM_152344.1	1.421361357	1.494939354	0.00046771
RAB10	ILMN_21971	NM_016131.2	1.417506897	1.724175804	0.00004435
RAB27A	ILMN_26122	NM_004580.3	1.415857452	2.439981834	0.00000007
PAK1	ILMN_3895	NM_002576.3	1.415428263	1.303528125	0.00000166
CYBB	ILMN_13274	NM_000397.2	1.408817731	1.339757578	0.00001259
GLCE	ILMN_19408	NM_015554.1	1.404828092	1.161063267	0.00063160
DAPK1	ILMN_138506	NM_004938.1	1.383669259	1.109733929	0.00016452
S100Z	ILMN_16143	NM_130772.1	1.382269138	0.991665875	0.00036217
STX7	ILMN_23974	NM_003569.1	1.38222876	1.653208921	0.00002049
RNF135	ILMN_26639	NM_032322.3	1.38208812	1.298243602	0.00018183
GOLPH3L	ILMN_29707	NM_018178.3	1.370986633	1.36453176	0.00063959
TST	ILMN_24453	NM_003312.4	1.369690016	1.136440543	0.00095333
SNX10	ILMN_21992	NM_013322.2	1.367198902	1.206295261	0.00011743
ZNF537	ILMN_19320	NM_020856.1	1.364283648	0.983156622	0.00049673
DPYD	ILMN_19002	NM_000110.2	1.360575553	1.221184847	0.00020880
THOC3	ILMN_17969	NM_032361.1	1.337370852	1.296516098	0.00010001
JAK2	ILMN_12566	NM_004972.2	1.336837147	1.157759338	0.00107764
CBR4	ILMN_15505	NM_032783.3	1.331554916	0.924736371	0.00572523
C6orf150	ILMN_18812	NM_138441.1	1.328798487	1.273871485	0.00029292
CNN2	ILMN_26898	NM_004368.2	1.328259599	0.862321574	0.00400179
FGR	ILMN_21930	NM_005248.1	1.327363076	1.39145794	0.00019357
CHN2	ILMN_16218	NM_004067.1	1.324958877	1.614430996	0.00000029
AMICA1	ILMN_14597	NM_153206.1	1.319782187	1.08065251	0.00325652
ZFP106	ILMN_6305	NM_022473.1	1.319592806	1.607493451	0.00000090

IGSF6	ILMN_7979	NM_005849.1	1.319368591	1.188636367	0.00037148
AP1S1	ILMN_21653	NM_001283.2	1.318735553	1.576995383	0.00001459
MCTP1	ILMN_2499	NM_001002796.1	1.315723721	1.202471994	0.00013997
ALDH3B1	ILMN_26797	NM_001030010.1	1.312159263	0.893300708	0.00194706
NFE2L3	ILMN_21009	NM_004289.5	1.308752905	1.300277296	0.00001069
MDM4	ILMN_137381	NM_002393.1	1.297256916	1.26641401	0.00155630
RP2	ILMN_28618	NM_006915.1	1.294332883	1.424661662	0.00003362
SLC35A1	ILMN_23284	NM_006416.2	1.290268239	1.163704098	0.00047859
KIAA1598	ILMN_4741	NM_018330.3	1.288926615	0.849412435	0.00370318
IL10RB	ILMN_26097	NM_000628.3	1.287949998	1.719776558	0.00000093
HNRPK	ILMN_16515	NM_031263.1	1.282519412	1.010295796	0.00375171
NUDT16	ILMN_16023	NM_152395.1	1.281298901	1.154766657	0.00007469
SLC35B3	ILMN_20545	NM_015948.2	1.278877271	1.051639038	0.00421060
LAT2	ILMN_137018	NM_022040.2	1.275195973	1.42216122	0.00000064
ZNF467	ILMN_11856	NM_207336.1	1.270946259	0.848454305	0.00395545
SIRPB1	ILMN_12074	NM_006065.1	1.270200218	1.037567054	0.00175406
TUBA3	ILMN_1089	NM_006009.2	1.267592173	1.335001342	0.00049465
IMPA2	ILMN_19881	NM_014214.1	1.265450116	1.022773847	0.00125011
FLJ11151	ILMN_19186	NM_018340.1	1.261665971	0.765786817	0.00229595
ZDHHC1	ILMN_29630	NM_013304.1	1.261345131	1.08082457	0.00052442
MLKL	ILMN_25241	NM_152649.1	1.253941489	1.285259718	0.00022319
SLC35A5	ILMN_26894	NM_017945.2	1.252447978	1.715944373	0.00001005
CRLF3	ILMN_22668	NM_015986.2	1.248375429	1.206829585	0.00060933
LOC387921	ILMN_1273	NM_001017370.1	1.244008484	1.120913984	0.00296565
PPP1CA	ILMN_26836	NM_002708.3	1.243778007	1.055486121	0.00011791
SH2D3C	ILMN_8703	NM_170600.1	1.243559889	1.020589358	0.00398947
AGPAT2	ILMN_6967	NM_006412.3	1.241156729	1.437884834	0.00000131
PYCARD	ILMN_1146	NM_145183.1	1.238338965	1.182704229	0.00014653
OXR1	ILMN_16599	NM_181354.3	1.233515739	0.943498189	0.00535975
LOC90693	ILMN_24365	NM_138771.2	1.228511696	1.070906703	0.00065741
EMR1	ILMN_12984	NM_001974.3	1.228478331	1.205910733	0.00011219

ATG7	ILMN_17242	NM_006395.1	1.226054413	1.111371355	0.00154238
HSD17B4	ILMN_23623	NM_000414.1	1.224568967	1.201685613	0.00127922
APLP2	ILMN_19935	NM_001642.1	1.221058814	0.783961377	0.00495608
LYST	ILMN_22965	NM_000081.2	1.220189239	0.990799094	0.00387086
FKBP1A	ILMN_29213	NM_054014.1	1.216668016	1.266978955	0.00058410
TCHP	ILMN_2569	NM_032300.2	1.214228384	0.967657406	0.00841717
PTPN6	ILMN_9886	NM_080549.2	1.213176953	1.147626146	0.00039216
ABHD3	ILMN_4359	NM_138340.3	1.21305899	1.376526741	0.00032576
HS1BP3	ILMN_1874	NM_022460.3	1.210718089	1.58016919	0.00000566
YIPF4	ILMN_16255	NM_032312.2	1.209690086	1.346340866	0.00002704
MTMR11	ILMN_137079	NM_181873.1	1.202420707	1.348571747	0.00095883
TPARL	ILMN_13352	NM_018475.2	1.200728921	1.062854741	0.00112553
TRIM5	ILMN_29177	NM_033092.1	1.19730344	2.098332255	0.00000187
CSF1R	ILMN_24980	NM_005211.2	1.193143878	1.081173199	0.00499259
SLC44A1	ILMN_22168	NM_022109.2	1.191671935	1.6856574	0.00002035
PCMT1	ILMN_26580	NM_005389.1	1.19084514	1.252373441	0.00023097
C5orf14	ILMN_16829	NM_024715.2	1.190668971	1.20283815	0.00007608
GIT2	ILMN_14647	NM_139201.1	1.190219346	2.052595042	0.00000291
KIAA0513	ILMN_21473	NM_014732.2	1.174748258	1.266650575	0.00002434
FAF1	ILMN_25532	NM_007051.2	1.174568781	1.118849274	0.00281882
TSPAN3	ILMN_10474	NM_005724.4	1.168225827	0.729580845	0.00438404
TFCP2	ILMN_22607	NM_005653.3	1.167647033	1.658661194	0.00005819
TMLHE	ILMN_29460	NM_018196.1	1.16485678	2.051181476	0.00000234
SDHA	ILMN_22058	NM_004168.1	1.161938606	1.022608707	0.00073498
C14orf147	ILMN_22701	NM_138288.2	1.160650505	1.112768537	0.00023392
ATP6V1A	ILMN_30226	NM_001690.2	1.15746526	1.754474554	0.00001328
LTA4H	ILMN_10760	NM_000895.1	1.153598722	1.390108277	0.00003130
GIMAP2	ILMN_20526	NM_015660.2	1.149577817	0.965671268	0.00193203
ME2	ILMN_2202	NM_002396.3	1.147878625	1.509287926	0.00002005
GLB1	ILMN_23626	NM_000404.1	1.146797283	1.690013295	0.00000041
KIAA0859	ILMN_17670	NM_015935.4	1.144598554	0.837769761	0.00626361

HHEX	**ILMN_137681**	**NM_002729.2**	**1.141884003**	**0.693529229**	**0.00647935**
IL15	**ILMN_16803**	**NM_172174.1**	**1.139875458**	**0.999399656**	**0.00054676**
NUPL2	**ILMN_2154**	**NM_007342.1**	**1.139731191**	**1.168744344**	**0.00024463**
ACADM	**ILMN_16589**	**NM_000016.2**	**1.13890795**	**0.924374337**	**0.00767478**
DHRS4	**ILMN_10152**	**NM_021004.2**	**1.137404903**	**1.634207884**	**0.00000856**
GLRX	**ILMN_22606**	**NM_002064.1**	**1.133988741**	**1.803719876**	**0.00005402**
HIATL1	**ILMN_445**	**NM_032558.1**	**1.128469227**	**1.556990239**	**0.00000072**
STAT1	**ILMN_29640**	**NM_007315.2**	**1.128402116**	**1.08223707**	**0.00124515**
RB1	**ILMN_4636**	**NM_000321.1**	**1.124836729**	**1.187181876**	**0.00086606**
BTK	**ILMN_28021**	**NM_000061.1**	**1.122372599**	**1.306551293**	**0.00018635**
GBGT1	**ILMN_18833**	**NM_021996.3**	**1.120301613**	**1.09684158**	**0.00144302**
MDFIC	**ILMN_21649**	**NM_199072.2**	**1.120203591**	**1.15768588**	**0.00176315**
EVI2B	**ILMN_26505**	**NM_006495.2**	**1.114183272**	**1.318566703**	**0.00003204**
ATP6V1D	**ILMN_26737**	**NM_015994.2**	**1.113501319**	**2.043668244**	**0.00000211**
TRAPPC6B	**ILMN_28066**	**NM_177452.2**	**1.113453002**	**1.012610594**	**0.00117111**
DYNC1I2	**ILMN_26959**	**NM_001378.1**	**1.113242907**	**1.315746836**	**0.00011227**
CAT	**ILMN_13962**	**NM_001752.2**	**1.110401051**	**1.241457245**	**0.00015781**
SAP18	**ILMN_27996**	**NM_005870.3**	**1.110157199**	**1.287921816**	**0.00045564**
RASSF4	**ILMN_1013**	**NM_178145.1**	**1.106395735**	**0.702632647**	**0.00971813**
TIPRL	**ILMN_13476**	**NM_152902.3**	**1.105926761**	**0.735464056**	**0.00753054**
GNAQ	**ILMN_18320**	**NM_002072.2**	**1.102538482**	**1.09049864**	**0.00052488**
GART	**ILMN_19282**	**NM_175085.1**	**1.100182823**	**1.860993741**	**0.00000013**
RNUT1	**ILMN_3859**	**NM_005701.2**	**1.098669993**	**1.252489255**	**0.00000760**
RSHL2	**ILMN_20992**	**NM_031924.3**	**1.098121903**	**1.201616713**	**0.00061853**
STAT1	**ILMN_11054**	**NM_139266.1**	**1.092967271**	**1.242374651**	**0.00019968**
LOC147804	**ILMN_772**	**NM_001010856.1**	**1.09060459**	**0.93550762**	**0.00192963**
GBP1	**ILMN_28413**	**NM_002053.1**	**1.088077859**	**0.818098264**	**0.00455937**
P2RY5	**ILMN_1003**	**NM_005767.3**	**1.084060495**	**0.944354401**	**0.00168605**
TSNAX	**ILMN_9331**	**NM_005999.2**	**1.083748338**	**0.920206882**	**0.00344845**
MSRB2	**ILMN_1402**	**NM_012228.2**	**1.082512282**	**1.138598039**	**0.00026016**
MAX	**ILMN_1660**	**NM_197957.2**	**1.080956724**	**1.359650229**	**0.00089655**

DHRS4L2	**ILMN_18916**	**NM_198083.1**	**1.0803122**	**1.833092933**	**0.00000279**
CD300A	**ILMN_20704**	**NM_007261.2**	**1.077905816**	**1.233438261**	**0.00011805**
HPS3	**ILMN_27156**	**NM_032383.3**	**1.077756305**	**1.166700056**	**0.00026715**
Kua-UEV	**ILMN_20084**	**NM_199203.1**	**1.077210942**	**0.890045394**	**0.00418034**
VRK2	**ILMN_12895**	**NM_006296.3**	**1.071093578**	**1.273308121**	**0.00006664**
PIGF	**ILMN_5303**	**NM_173074.1**	**1.069955594**	**1.32315317**	**0.00196465**
KIAA0907	**ILMN_6810**	**NM_014949.2**	**1.068392034**	**0.773879851**	**0.00546443**
ITGA4	**ILMN_20825**	**NM_000885.4**	**1.064564534**	**1.296599118**	**0.00066087**
C14orf100	**ILMN_2292**	**NM_016475.2**	**1.062074722**	**1.549919473**	**0.00005950**
C20orf55	**ILMN_7928**	**NM_031424.3**	**1.061601137**	**1.584276472**	**0.00005688**
FLJ30655	**ILMN_19208**	**NM_144643.1**	**1.060282183**	**0.876173172**	**0.00111608**
PEX11B	**ILMN_20603**	**NM_003846.1**	**1.06008193**	**1.153357146**	**0.00010047**
RTN3	**ILMN_20331**	**NM_006054.2**	**1.057527171**	**1.028960785**	**0.00040536**
TRPM4	**ILMN_23519**	**NM_017636.2**	**1.05591347**	**0.961545232**	**0.00029359**
RHOT1	**ILMN_7051**	**NM_001033568.1**	**1.053669109**	**1.180698251**	**0.00005482**
STX10	**ILMN_14838**	**NM_003765.1**	**1.052249524**	**1.128504094**	**0.00001413**
COMMD10	**ILMN_12276**	**NM_016144.2**	**1.050382398**	**1.495031129**	**0.00001106**
CD1C	**ILMN_25152**	**NM_001765.1**	**1.048258496**	**0.887735273**	**0.00455867**
CTSS	**ILMN_13149**	**NM_004079.3**	**1.045734735**	**0.82615845**	**0.00252149**
WARS	**ILMN_25430**	**NM_213646.1**	**1.04496961**	**1.189056087**	**0.00237587**
KCNK6	**ILMN_26070**	**NM_004823.1**	**1.044519702**	**1.771355806**	**0.00000007**
RGS19	**ILMN_137691**	**NM_005873.1**	**1.043344803**	**1.22908987**	**0.00190992**
TRADD	**ILMN_27933**	**NM_003789.2**	**1.04251768**	**1.262244624**	**0.00001350**
CTDSP1	**ILMN_13739**	**NM_182642.1**	**1.041515241**	**0.971166874**	**0.00278989**
CHCHD4	**ILMN_27470**	**NM_144636.1**	**1.040667368**	**1.271820439**	**0.00115294**
DPYSL2	**ILMN_9671**	**NM_001386.4**	**1.040441672**	**1.184566634**	**0.00043079**
OSBPL11	**ILMN_21883**	**NM_022776.3**	**1.040366673**	**0.902785221**	**0.00280335**
PPID	**ILMN_7536**	**NM_005038.2**	**1.040245336**	**0.920803978**	**0.00114088**
KLRG1	**ILMN_12613**	**NM_005810.3**	**1.031994693**	**0.951340998**	**0.00551552**
TNRC5	**ILMN_25587**	**NM_006586.2**	**1.031678935**	**1.226161091**	**0.00013477**
C1orf25	**ILMN_6315**	**NM_030934.3**	**1.029790058**	**0.922590503**	**0.00311092**

BTN3A3	ILMN_20620	NM_006994.3	1.02955062	0.986674751	0.00505231
HMGCL	ILMN_18301	NM_000191.2	1.028869289	0.988156967	0.00145224
RNASEL	ILMN_25186	NM_021133.2	1.028462688	0.908939728	0.00343962
THRAP3	ILMN_5104	NM_005119.2	1.027723448	1.15862836	0.00424330
ERICH1	ILMN_15474	NM_207332.1	1.02729451	1.505330849	0.00008397
SETD3	ILMN_27724	NM_199123.1	1.026914874	1.847519372	0.00000848
LOC339745	ILMN_21364	NM_001001664.1	1.025194	1.043785199	0.00044059
SYK	ILMN_8126	NM_003177.3	1.02306596	1.069140141	0.00058954
LYPLAL1	ILMN_25005	NM_138794.1	1.021749864	1.274393317	0.00017061
CXorf38	ILMN_24514	NM_144970.1	1.018860976	0.993458358	0.00153043
MFSD1	ILMN_29705	NM_022736.1	1.016547521	0.960614309	0.00809506
COG5	ILMN_10374	NM_006348.2	1.014274311	0.882601775	0.00214891
CTSC	ILMN_14007	NM_001814.2	1.01344871	1.853389446	0.00000125
ARSD	ILMN_23258	NM_001669.2	1.013248964	1.084414068	0.00009669
C10orf78	ILMN_1251	NM_145247.4	1.009571689	0.988084592	0.00097754
IHPK2	ILMN_4437	NM_001005911.1	1.00704733	0.951288743	0.00714339
MGST2	ILMN_8759	NM_002413.3	1.006871413	0.89556421	0.00323860
PQLC3	ILMN_138926	NM_152391.2	1.006106122	1.472969297	0.00001380
APIP	ILMN_15379	NM_015957.1	1.004926744	1.433668335	0.00000145
FES	ILMN_27340	NM_002005.2	1.002838995	1.0193433	0.00138719
LOC51136	ILMN_27239	NM_016125.2	1.002029878	1.193124036	0.00048848
C1orf162	ILMN_19455	NM_174896.2	0.99955481	1.402962342	0.00028599
LOC441268	ILMN_18016	NM_001013725.1	0.996288768	1.130499126	0.00002006
GCNT1	ILMN_28747	NM_001490.3	0.995457433	0.869013003	0.00381332
ARL6IP6	ILMN_11477	NM_152522.2	0.994288632	1.477088131	0.00001328
OAZ2	ILMN_28141	NM_002537.1	0.994156749	1.166412733	0.00001391
FLJ40142	ILMN_5279	NM_207435.1	0.993492071	1.165314596	0.00045441
SP1	ILMN_23737	NM_138473.2	0.993032055	1.260719548	0.00015175
ATG3	ILMN_18856	NM_022488.3	0.988228041	1.916410319	0.00000002
LYL1	ILMN_18317	NM_005583.3	0.987845869	1.06009773	0.00044087
ASPHD2	ILMN_16388	NM_020437.3	0.984948065	1.845832042	0.00000932

DTYMK	ILMN_13450	NM_012145.2	0.984864928	1.210032184	0.00035093
PIGV	ILMN_3048	NM_017837.2	0.978814247	0.991495087	0.00284743
PROSC	ILMN_23472	NM_007198.2	0.977585431	1.202805016	0.00047291
CIDEB	ILMN_11569	NM_014430.1	0.977506182	1.25429654	0.00018715
SIRPB2	ILMN_26592	NM_018556.2	0.977420368	0.697565081	0.00999559
C20orf155	ILMN_14031	NM_019095.3	0.976876389	1.396760216	0.00013889
C3orf1	ILMN_7612	NM_016589.3	0.973048163	1.328604302	0.00090971
NAGA	ILMN_21273	NM_000262.1	0.972941111	0.743299601	0.00545547
NUP214	ILMN_29992	NM_005085.2	0.972928088	1.043626148	0.00010851
MTPN	ILMN_28124	NM_145808.1	0.967281848	1.009440702	0.00033972
SUCLA2	ILMN_5448	NM_003850.1	0.966750454	1.204571152	0.00003408
RNF14	ILMN_4655	NM_004290.3	0.965790647	1.08994806	0.00023317
SPCS3	ILMN_14718	NM_021928.1	0.965416591	1.255510062	0.00001553
IL17R	ILMN_19124	NM_014339.3	0.964912628	0.686029738	0.00622337
C7orf24	ILMN_2391	NM_024051.2	0.962521409	1.580715796	0.00000269
ATP8B4	ILMN_20882	NM_024837.2	0.960699332	1.080063592	0.00398716
CENTB2	ILMN_8806	NM_012287.3	0.960173513	0.856922854	0.00407885
ETNK1	ILMN_137282	NM_018638.3	0.960101883	1.078610828	0.00061539
C8orf70	ILMN_13979	NM_016010.1	0.957844064	0.932397929	0.00250075
SLC33A1	ILMN_18559	NM_004733.2	0.95284096	0.836978907	0.00891011
MINPP1	ILMN_29353	NM_004897.2	0.952445582	0.841313401	0.00437668
FLJ32028	ILMN_2086	NM_152680.1	0.951166051	1.157097549	0.00059476
TMEM69	ILMN_22324	NM_016486.2	0.94461557	1.735210786	0.00000014
TCEA1	ILMN_15877	NM_006756.2	0.943962517	1.032769437	0.00032666
ZFYVE21	ILMN_1317	NM_024071.2	0.939951758	1.266235738	0.00060070
C1GALT1C1	ILMN_14223	NM_001011551.1	0.939682644	1.268820331	0.00042353
MRPL18	ILMN_14120	NM_014161.2	0.939047175	1.211864841	0.00003859
COMMD8	ILMN_16165	NM_017845.2	0.938884957	1.211204735	0.00004943
SUOX	ILMN_25326	NM_001032386.1	0.938099751	1.116839515	0.00021507
SEC23IP	ILMN_7522	NM_007190.2	0.935305956	1.050976614	0.00162119
SLC27A3	ILMN_820	NM_024330.1	0.929556446	1.03995997	0.00420275

TRFP	ILMN_10817	NM_004275.3	0.927887255	1.031529777	0.00025827
RAB24	ILMN_21668	NM_130781.1	0.925882602	1.376496871	0.00034727
ARRB1	ILMN_7318	NM_004041.3	0.925483191	1.287394226	0.00045584
OMA1	ILMN_29719	NM_145243.3	0.923738733	1.043094562	0.00683567
MGC39633	ILMN_25725	NM_152549.1	0.922176038	0.766026212	0.00863450
NIF3L1	ILMN_20423	NM_021824.2	0.921438655	0.937519896	0.00512003
SPTLC1	ILMN_10107	NM_006415.2	0.920711882	1.135911491	0.00084213
RAB28	ILMN_13007	NM_001017979.1	0.920578297	0.870380016	0.00570634
ICAM2	ILMN_12334	NM_000873.2	0.918459715	1.379872474	0.00003721
BET1	ILMN_137098	NM_005868.3	0.915908909	1.015505469	0.00049841
DPAGT1	ILMN_10306	NM_001382.2	0.914750765	1.37671312	0.00026551
MAPK1	ILMN_1351	NM_138957.2	0.912944589	1.973641533	0.00000218
CREB1	ILMN_4653	NM_004379.2	0.912153152	1.289898949	0.00000751
FLJ11806	ILMN_22941	NM_024824.3	0.907043224	0.872607897	0.00251841
GBP4	ILMN_2749	NM_052941.2	0.906318346	0.884424018	0.00165022
UBE1C	ILMN_22726	NM_198197.1	0.906055038	1.312237849	0.00001150
HTATIP2	ILMN_16008	NM_006410.3	0.90509346	0.948238591	0.00323868
DNCL1	ILMN_137049	NM_003746.1	0.904688718	1.039220175	0.00434549
ATG16L2	ILMN_16649	NM_033388.1	0.904500406	0.687753427	0.00455909
RAB28	ILMN_23100	NM_004249.1	0.904454284	1.247203021	0.00004258
SDHD	ILMN_6353	NM_003002.1	0.903802405	0.915922902	0.00148158
CMAS	ILMN_21201	NM_018686.3	0.901806436	1.556521592	0.00002760
SLC39A3	ILMN_27676	NM_144564.4	0.899190336	1.175928733	0.00003123
GALK1	ILMN_18040	NM_000154.1	0.896328132	1.079109296	0.00381481
TMEM60	ILMN_19683	NM_032936.2	0.896262347	1.461391638	0.00006428
AHNAK	ILMN_28759	NM_001620.1	0.893847691	0.663070451	0.00502640
SNX5	ILMN_3880	NM_014426.2	0.89245185	0.888484828	0.00978382
TMED10	ILMN_4416	NM_006827.4	0.888532499	1.15987317	0.00373153
MDM1	ILMN_12153	NM_017440.2	0.885584244	1.052871085	0.00008682
LOC129531	ILMN_27516	NM_138798.1	0.884116434	1.128519561	0.00309031
MTSS1	ILMN_7083	NM_014751.2	0.882192231	0.971455139	0.00044650

FLJ20534	ILMN_9467	NM_017867.1	0.881491211	1.247739397	0.00032511
NACA	ILMN_17162	NM_005594.2	0.879209492	1.329963923	0.00013907
GDI2	ILMN_15402	NM_001494.2	0.876874596	1.421630896	0.00008006
SNX2	ILMN_4948	NM_003100.2	0.876247823	0.935068996	0.00051003
ARRDC1	ILMN_11748	NM_152285.2	0.875635011	1.073875983	0.00011921
ARV1	ILMN_5909	NM_022786.1	0.874609136	0.896227084	0.00931000
THAP1	ILMN_15754	NM_018105.2	0.871272902	0.976199365	0.00181390
REEP5	ILMN_21319	NM_005669.3	0.871021946	0.904693424	0.00161578
PHCA	ILMN_3836	NM_018367.3	0.866524089	1.002767545	0.00079992
VAMP4	ILMN_139375	NM_003762.2	0.865903861	1.174684728	0.00141188
RAB7	ILMN_30038	NM_004637.5	0.861414853	1.222343087	0.00108071
COQ5	ILMN_6126	NM_032314.2	0.859134948	0.991809815	0.00432292
TKT	ILMN_2726	NM_001064.1	0.859005488	0.916255715	0.00097002
MDS025	ILMN_6208	NM_021825.3	0.858810512	0.793814684	0.00814564
WDR40A	ILMN_29238	NM_015397.1	0.857153805	0.981080156	0.00634603
NOMO1	ILMN_22691	NM_014287.3	0.856327308	1.032285196	0.00044624
PLAC8	ILMN_17809	NM_016619.1	0.853759799	0.772570372	0.00784848
UNC50	ILMN_19045	NM_014044.4	0.853303767	1.843345515	0.00000260
TM9SF1	ILMN_1371	NM_001014842.1	0.852681312	1.044733631	0.00020166
THEM2	ILMN_27212	NM_018473.2	0.852676742	1.164355119	0.00142445
TRIP4	ILMN_10213	NM_016213.3	0.849722307	1.77715718	0.00000087
MGC15875	ILMN_28180	NM_153373.1	0.848075084	0.773122313	0.00731177
CLK3	ILMN_1044	NM_003992.1	0.845775564	0.958931247	0.00082384
CORO1A	ILMN_6768	NM_007074.2	0.844780702	1.132107408	0.00013626
CRIPT	ILMN_12903	NM_014171.3	0.844040876	1.202882939	0.00161066
SDFR1	ILMN_138444	NM_012428.1	0.842592487	0.771466242	0.00266393
ATP6V0E	ILMN_8923	NM_003945.3	0.841844604	1.529777909	0.00000753
TMCO3	ILMN_19218	NM_017905.3	0.838962707	1.176980562	0.00451413
SNAP29	ILMN_20709	NM_004782.2	0.836938792	1.255622288	0.00003215
NCK1	ILMN_13975	NM_006153.3	0.833950007	0.911191822	0.00105238
SNX1	ILMN_924	NM_003099.3	0.832963832	0.778505884	0.00455591

PRKAR1A	**ILMN_18925**	**NM_002734.3**	**0.832024455**	**1.26445346**	**0.00085621**
HLA-DPA1	**ILMN_13606**	**NM_033554.2**	**0.830818992**	**1.198741802**	**0.00006353**
FKBP1A	**ILMN_1290**	**NM_000801.2**	**0.82816191**	**1.376397268**	**0.00068549**
CHKB	**ILMN_1067**	**NM_005198.3**	**0.827517927**	**1.035599235**	**0.00183007**
MGC16385	**ILMN_5329**	**NM_145039.2**	**0.8274803**	**0.794442997**	**0.00790513**
ARPC4	**ILMN_24112**	**NM_001024959.1**	**0.82741729**	**1.300843186**	**0.00000283**
C21orf33	**ILMN_28752**	**NM_004649.4**	**0.821418828**	**0.990826614**	**0.00317030**
TES	**ILMN_17251**	**NM_015641.2**	**0.819022224**	**0.734094965**	**0.00560739**
STT3A	**ILMN_17585**	**NM_152713.2**	**0.816670936**	**1.287957745**	**0.00003264**
TMEM126B	**ILMN_18826**	**NM_018480.2**	**0.813364425**	**1.16222445**	**0.00148392**
NDUFAF1	**ILMN_13182**	**NM_016013.2**	**0.810438154**	**1.268624194**	**0.00045587**
WRB	**ILMN_12263**	**NM_004627.2**	**0.809280188**	**1.063669514**	**0.00039734**
C17orf62	**ILMN_12733**	**NM_001033046.1**	**0.808445826**	**0.873210482**	**0.00057408**
LOC339344	**ILMN_6535**	**NM_001012643.2**	**0.80801784**	**0.88574801**	**0.00770728**
UTRN	**ILMN_15375**	**NM_007124.1**	**0.806804848**	**1.062976949**	**0.00038025**
YIF1B	**ILMN_10933**	**NM_001031731.1**	**0.805625909**	**0.827041041**	**0.00081460**
WDR23	**ILMN_11121**	**NM_025230.3**	**0.80464906**	**0.713414493**	**0.00387877**
CG018	**ILMN_24274**	**NM_052818.1**	**0.804367155**	**1.004875505**	**0.00158518**
CCDC5	**ILMN_18943**	**NM_138443.2**	**0.803601045**	**0.811374754**	**0.00126376**
MAP3K3	**ILMN_426**	**NM_203351.1**	**0.801573187**	**0.933775478**	**0.00008829**
PAQR4	**ILMN_20105**	**NM_152341.2**	**0.80144963**	**0.85738589**	**0.00700293**
RBM4	**ILMN_11057**	**NM_002896.1**	**0.800689376**	**1.000393552**	**0.00852204**
FLJ10260	**ILMN_454**	**NM_018042.2**	**0.799519136**	**1.101275589**	**0.00004981**
C14orf159	**ILMN_22928**	**NM_024952.4**	**0.79931973**	**1.070855583**	**0.00137924**
CRYZL1	**ILMN_5113**	**NM_005111.5**	**0.797728559**	**1.206417193**	**0.00010391**
COQ9	**ILMN_16451**	**NM_020312.1**	**0.795958206**	**1.041376851**	**0.00209683**
PCK2	**ILMN_2603**	**NM_004563.2**	**0.793276194**	**0.970467923**	**0.00068396**
EXOSC3	**ILMN_9043**	**NM_016042.2**	**0.790073504**	**1.066489341**	**0.00024318**
RER1	**ILMN_16490**	**NM_007033.2**	**0.788900128**	**1.091279047**	**0.00011252**
TM9SF1	**ILMN_1249**	**NM_006405.5**	**0.788259179**	**1.468086787**	**0.00004208**
ACN9	**ILMN_6296**	**NM_020186.1**	**0.787176157**	**0.883990806**	**0.00311959**

CTSO	ILMN_22132	NM_001334.2	0.786911991	0.842038184	0.00148380
DHRS8	ILMN_12219	NM_016245.2	0.784360455	0.788762086	0.00171737
MRPL30	ILMN_137901	NM_145212.1	0.783716628	0.777905407	0.00207844
BANF1	ILMN_13154	NM_003860.2	0.781911821	0.91109998	0.00759135
C10orf6	ILMN_24540	NM_018121.2	0.78063571	0.697345556	0.00689460
HLA-F	ILMN_3012	NM_018950.1	0.778737753	0.931623998	0.00211549
C7orf28B	ILMN_8655	NM_198097.1	0.777924185	0.933647594	0.00693097
KIAA0152	ILMN_24410	NM_014730.2	0.777856443	0.76897219	0.00436356
DSCR1	ILMN_3292	NM_203417.1	0.776155691	0.909602389	0.00619489
UBE2G1	ILMN_4331	NM_003342.4	0.776077148	1.539756439	0.00012246
AKR1A1	ILMN_18164	NM_006066.2	0.775609482	1.438402297	0.00003774
RNPEP	ILMN_22895	NM_020216.3	0.773848693	0.798178296	0.00210507
PDCD10	ILMN_26567	NM_145859.1	0.773529175	0.734718047	0.00444282
TIA1	ILMN_29910	NM_022173.1	0.771619932	1.012734353	0.00088562
C17orf40	ILMN_18247	NM_018428.2	0.770526096	1.085875486	0.00034219
GABPA	ILMN_28392	NM_002040.2	0.769932318	0.918905978	0.00099307
TADA3L	ILMN_7198	NM_006354.2	0.76569954	1.159110327	0.00058551
GNPAT	ILMN_15390	NM_014236.1	0.765579192	1.098910095	0.00028603
IIP45	ILMN_12607	NM_001025374.1	0.763584039	1.107873404	0.00010382
PSMA5	ILMN_15259	NM_002790.2	0.762135923	1.581564317	0.00011433
SUMF1	ILMN_15497	NM_182760.2	0.759267277	0.823262053	0.00496238
PDCD8	ILMN_20381	NM_004208.2	0.757914227	1.319956996	0.00015773
SCO1	ILMN_14321	NM_004589.1	0.757545888	1.364663218	0.00000441
DUSP18	ILMN_9044	NM_152511.3	0.756268314	0.953020427	0.00032814
RAB24	ILMN_25731	NM_001031677.1	0.754549224	0.988549939	0.00110535
DUSP3	ILMN_14523	NM_004090.2	0.753530167	0.950437848	0.00581700
EPS15	ILMN_8810	NM_001981.2	0.752852195	0.971791525	0.00324139
CCDC53	ILMN_25394	NM_016053.1	0.752132981	1.108722086	0.00761538
RHBDD1	ILMN_13013	NM_032276.2	0.749264488	1.440263385	0.00014650
UTP14A	ILMN_3222	NM_006649.2	0.746978261	0.722920018	0.00572518
APOL3	ILMN_8572	NM_030644.1	0.745532666	0.774795383	0.00253167

AHCYL1	ILMN_21551	NM_006621.3	0.743721598	1.182785059	0.00012557
D15Wsu75e	ILMN_13640	NM_015704.1	0.743698121	1.437551529	0.00004606
FAM45A	ILMN_28113	NM_207009.2	0.739449537	0.80784723	0.00076796
WDR58	ILMN_25260	NM_024339.2	0.739211399	0.855804331	0.00209070
LMBRD1	ILMN_6684	NM_018368.2	0.737434728	1.633121026	0.00000048
GMPR2	ILMN_15872	NM_001002002.1	0.736748124	1.047904062	0.00098527
ACTL6A	ILMN_3186	NM_177989.1	0.736251612	0.955844795	0.00063734
TP53I3	ILMN_2007	NM_147184.1	0.735509717	0.697604939	0.00877149
BRD8	ILMN_10431	NM_183359.1	0.735297102	0.808354111	0.00087882
LIPT1	ILMN_5728	NM_145197.1	0.734857538	0.949720387	0.00015131
TMBIM4	ILMN_22938	NM_016056.1	0.734512096	1.354611683	0.00113048
RSU1	ILMN_21628	NM_012425.3	0.734466102	0.776631195	0.00791837
HLA-DMB	ILMN_2252	NM_002118.3	0.73405362	1.234805982	0.00059628
C14orf142	ILMN_28666	NM_032490.3	0.731225916	1.03362714	0.00244109
BCKDK	ILMN_13829	NM_005881.1	0.729788087	0.823487241	0.00620961
ARHGAP25	ILMN_1674	NM_001007231.1	0.728785554	1.031541589	0.00812840
NDUFB5	ILMN_20286	NM_002492.2	0.727040149	0.978032175	0.00114697
GEMIN6	ILMN_23187	NM_024775.9	0.726343401	0.982878344	0.00246154
ATP6V0A1	ILMN_138088	NM_005177.2	0.725439284	1.1407963	0.00647840
GLT25D1	ILMN_15022	NM_024656.2	0.725314591	1.067096598	0.00025548
BCL2L2	ILMN_9171	NM_004050.2	0.724745898	1.030677194	0.00170958
YIPF6	ILMN_8545	NM_173834.2	0.724660912	1.28290882	0.00004430
WDR71	ILMN_13656	NM_025155.1	0.724614947	1.053661809	0.00164536
GNS	ILMN_6937	NM_002076.2	0.723930284	1.641619517	0.00011972
P2RX7	ILMN_26570	NM_002562.4	0.718844427	0.869779162	0.00359117
C2orf18	ILMN_138677	NM_017877.2	0.716007244	0.736293165	0.00574343
TTL	ILMN_14027	NM_153712.3	0.715465204	1.444770368	0.00007258
FRAT1	ILMN_29787	NM_181355.1	0.714693512	0.885429679	0.00187052
HTLF	ILMN_10159	NM_002158.2	0.711836675	0.847706272	0.00958303
MT	ILMN_5185	NM_173467.3	0.71124282	1.539123007	0.00000114
SELL	ILMN_6951	NM_000655.2	0.709452209	0.745836628	0.00525236

OSTF1	**ILMN_30131**	**NM_012383.3**	**0.707754417**	**2.255587056**	**0.00000318**
NARS	**ILMN_6062**	**NM_004539.2**	**0.706148703**	**0.738215334**	**0.00558703**
PIGM	**ILMN_19117**	**NM_145167.1**	**0.705962924**	**0.835125048**	**0.00054362**
PANK2	**ILMN_137507**	**NM_153640.1**	**0.705179063**	**1.272416067**	**0.00005978**
NOD9	**ILMN_16473**	**NM_024618.2**	**0.705010782**	**0.955293557**	**0.00679340**
SLC36A1	**ILMN_22384**	**NM_078483.2**	**0.701269954**	**1.12297335**	**0.00552217**
LYSMD2	**ILMN_19682**	**NM_153374.1**	**0.699845822**	**1.003581606**	**0.00039068**
NUP37	**ILMN_4147**	**NM_024057.2**	**0.69729722**	**1.115484685**	**0.00107876**
WDFY2	**ILMN_14243**	**NM_052950.2**	**0.696920753**	**1.270865789**	**0.00000365**
MAT2B	**ILMN_18923**	**NM_013283.3**	**0.694070182**	**0.921791826**	**0.00150174**
GSR	**ILMN_14467**	**NM_000637.2**	**0.693682753**	**1.446611933**	**0.00002356**
ING1	**ILMN_2492**	**NM_198219.1**	**0.691970285**	**1.062073688**	**0.00034920**
UBE2L6	**ILMN_7531**	**NM_004223.3**	**0.690995994**	**1.221401827**	**0.00442322**
DUSP22	**ILMN_15436**	**NM_020185.3**	**0.689864834**	**1.207246339**	**0.00007646**
DERA	**ILMN_16833**	**NM_015954.1**	**0.688508854**	**0.82267023**	**0.00331905**
FLJ39370	**ILMN_18245**	**NM_152400.1**	**0.68769568**	**0.751426804**	**0.00347573**
CNDP2	**ILMN_4139**	**NM_018235.1**	**0.687168603**	**1.093880054**	**0.00422201**
DNTTIP1	**ILMN_17783**	**NM_052951.2**	**0.683667693**	**0.854101583**	**0.00079144**
PSMB9	**ILMN_12611**	**NM_002800.4**	**0.67953923**	**0.73246271**	**0.00985021**
FAM96A	**ILMN_13416**	**NM_032231.4**	**0.675960026**	**1.07088344**	**0.00104755**
COPS7A	**ILMN_13902**	**NM_016319.1**	**0.675795633**	**1.141314182**	**0.00345432**
COMT	**ILMN_2463**	**NM_000754.2**	**0.675541976**	**0.859080355**	**0.00648443**
SPAST	**ILMN_15461**	**NM_199436.1**	**0.67529387**	**0.997006821**	**0.00142484**
RNF34	**ILMN_16571**	**NM_025126.2**	**0.673203274**	**0.704105246**	**0.00841035**
YIPF1	**ILMN_19321**	**NM_018982.3**	**0.670580284**	**2.072819422**	**0.00000023**
NEDD8	**ILMN_21274**	**NM_006156.1**	**0.669855809**	**0.674729549**	**0.00308767**
MINA	**ILMN_18661**	**NM_153182.1**	**0.668899752**	**1.787534969**	**0.00001651**
MGC40405	**ILMN_1247**	**NM_152789.1**	**0.66835355**	**0.65432264**	**0.00533856**
STX12	**ILMN_18776**	**NM_177424.1**	**0.667206523**	**0.672081087**	**0.00821280**
VPS41	**ILMN_2386**	**NM_014396.2**	**0.665669394**	**1.039923735**	**0.00131977**
C14orf133	**ILMN_19051**	**NM_022067.2**	**0.66448481**	**1.064551011**	**0.00005755**

ALDH3A2	**ILMN_15293**	**NM_001031806.1**	**0.663494351**	**1.225806952**	**0.00114718**
PHKB	**ILMN_18544**	**NM_001031835.1**	**0.663202945**	**0.828357174**	**0.00199503**
PINK1	**ILMN_27027**	**NM_032409.1**	**0.663117176**	**1.392356599**	**0.00017973**
FLJ33814	**ILMN_21814**	**NM_173510.1**	**0.662289635**	**0.786509978**	**0.00310654**
PCCA	**ILMN_6045**	**NM_000282.2**	**0.662170417**	**0.875641136**	**0.00671001**
PYCR2	**ILMN_18209**	**NM_013328.2**	**0.662158353**	**0.778688542**	**0.00586486**
CPSF2	**ILMN_12041**	**NM_017437.1**	**0.661956716**	**0.678957119**	**0.00474164**
UBE2E2	**ILMN_29196**	**NM_152653.1**	**0.6614415**	**1.189101101**	**0.00016503**
TAF12	**ILMN_3797**	**NM_005644.2**	**0.661208219**	**1.538640535**	**0.00001332**
SLC25A1	**ILMN_28181**	**NM_005984.1**	**0.659489816**	**0.636752915**	**0.00404682**
SEC11L1	**ILMN_12976**	**NM_014300.2**	**0.65876876**	**1.279650634**	**0.00001105**
DNAJB6	**ILMN_7651**	**NM_058246.3**	**0.655667179**	**1.054957112**	**0.00048058**
KLHL8	**ILMN_19798**	**NM_020803.3**	**0.654892324**	**0.9023516**	**0.00179519**
ACO1	**ILMN_9544**	**NM_002197.1**	**0.653596004**	**1.252199889**	**0.00025310**
GTF2H3	**ILMN_9713**	**NM_001516.3**	**0.652562825**	**0.762186527**	**0.00523155**
LSM10	**ILMN_14450**	**NM_032881.1**	**0.652247983**	**0.934453562**	**0.00090340**
NAG8	**ILMN_21136**	**NM_014411.2**	**0.651659684**	**0.654467349**	**0.00549902**
ATP5F1	**ILMN_138139**	**NM_001688.3**	**0.650324011**	**1.6471075**	**0.00000221**
NSF	**ILMN_23282**	**NM_006178.1**	**0.649787429**	**0.966754147**	**0.00150748**
C14orf129	**ILMN_7725**	**NM_016472.3**	**0.649488365**	**0.845576993**	**0.00222523**
C7orf11	**ILMN_20229**	**NM_138701.1**	**0.648424024**	**1.030461324**	**0.00188419**
RAB4A	**ILMN_14453**	**NM_004578.2**	**0.645974184**	**1.119336929**	**0.00016381**
PAPSS1	**ILMN_6621**	**NM_005443.4**	**0.644433151**	**1.089439803**	**0.00684807**
RAB8A	**ILMN_8881**	**NM_005370.4**	**0.643908926**	**2.486726255**	**0.00000007**
SFT2D1	**ILMN_5727**	**NM_145169.1**	**0.643517185**	**1.381139093**	**0.00017387**
C5orf15	**ILMN_23157**	**NM_020199.1**	**0.641504014**	**1.369919922**	**0.00000815**
STIP1	**ILMN_28761**	**NM_006819.1**	**0.639747132**	**0.914075119**	**0.00037943**
TRIM22	**ILMN_532**	**NM_006074.2**	**0.639603182**	**1.16001109**	**0.00076658**
CD84	**ILMN_16790**	**NM_003874.1**	**0.637727242**	**0.674191429**	**0.00524555**
CCT7	**ILMN_22959**	**NM_006429.2**	**0.630546718**	**1.001568609**	**0.00117525**
TXNDC	**ILMN_13849**	**NM_030755.3**	**0.629887162**	**0.839765388**	**0.00214526**

GALM	**ILMN_10698**	**NM_138801.1**	**0.629606832**	**1.079055934**	**0.00020824**
AP3B1	**ILMN_4527**	**NM_003664.3**	**0.628411492**	**0.774722262**	**0.00530390**
YBX1	**ILMN_2951**	**NM_004559.2**	**0.626746918**	**0.799211438**	**0.00576257**
CPOX	**ILMN_28523**	**NM_000097.4**	**0.62547916**	**0.888055102**	**0.00258340**
SNTB1	**ILMN_23979**	**NM_021021.2**	**0.625010436**	**0.894065332**	**0.00935770**
VPS72	**ILMN_17901**	**NM_005997.1**	**0.62278748**	**1.26711063**	**0.00030014**
ATP6V0D1	**ILMN_139373**	**NM_004691.3**	**0.62147397**	**0.730172626**	**0.00229783**
SUMF2	**ILMN_9757**	**NM_015411.1**	**0.617287604**	**0.874405945**	**0.00843465**
MR1	**ILMN_10108**	**NM_001531.1**	**0.615780365**	**0.968113548**	**0.00993067**
TRAFD1	**ILMN_21539**	**NM_006700.1**	**0.613875843**	**0.571258077**	**0.00960467**
HK1	**ILMN_23877**	**NM_033500.1**	**0.612449234**	**1.011322954**	**0.00092195**
P4HA1	**ILMN_8003**	**NM_000917.2**	**0.611236652**	**0.72707231**	**0.00625872**
FLJ14466	**ILMN_29870**	**NM_032790.2**	**0.61089811**	**0.830612766**	**0.00172031**
PSMD4	**ILMN_137384**	**NM_002810.1**	**0.610740993**	**0.757697935**	**0.00101724**
C10orf61	**ILMN_24822**	**NM_001013840.1**	**0.610458973**	**1.081393619**	**0.00013008**
FKBP4	**ILMN_9429**	**NM_002014.2**	**0.609790605**	**0.822235317**	**0.00292538**
CDK2AP1	**ILMN_19522**	**NM_004642.2**	**0.608899789**	**0.746051772**	**0.00607617**
PBX3	**ILMN_23493**	**NM_006195.4**	**0.608542479**	**1.042523333**	**0.00033834**
CCNC	**ILMN_11667**	**NM_005190.3**	**0.60615937**	**0.773035605**	**0.00270549**
PRCP	**ILMN_2019**	**NM_199418.1**	**0.605288708**	**1.075108698**	**0.00129425**
GBE1	**ILMN_2925**	**NM_000158.1**	**0.605174564**	**1.414871406**	**0.00066951**
LRCH3	**ILMN_14018**	**NM_032773.2**	**0.603643585**	**0.813171638**	**0.00619821**
POLR2E	**ILMN_21511**	**NM_002695.2**	**0.603012311**	**0.990775486**	**0.00027094**
PGM2	**ILMN_6226**	**NM_018290.2**	**0.601475444**	**0.722208068**	**0.00527047**
NCOA4	**ILMN_24328**	**NM_005437.1**	**0.600193396**	**1.108888989**	**0.00054757**
CD37	**ILMN_20101**	**NM_001774.1**	**0.600048449**	**0.728222604**	**0.00438458**
GGPS1	**ILMN_137664**	**NM_004837.2**	**0.599117516**	**0.84254707**	**0.00184079**
CCT6A	**ILMN_3686**	**NM_001009186.1**	**0.598843562**	**0.840343858**	**0.00148873**
HP1BP3	**ILMN_29502**	**NM_016287.2**	**0.598707576**	**0.947341533**	**0.00169712**
TRA16	**ILMN_20587**	**NM_176880.4**	**0.598636525**	**0.823603103**	**0.00530158**
ETFA	**ILMN_138729**	**NM_000126.1**	**0.597849505**	**1.12408418**	**0.00015177**

VAMP5	**ILMN_20179**	**NM_006634.2**	**0.597238144**	**0.71886071**	**0.00463721**
TSEN34	**ILMN_5948**	**NM_024075.1**	**0.597201061**	**1.116530396**	**0.00036174**
C9orf23	**ILMN_3926**	**NM_148178.1**	**0.595942735**	**0.846713274**	**0.00534341**
ACAT1	**ILMN_29522**	**NM_000019.2**	**0.594932008**	**0.719532236**	**0.00305532**
MRPL48	**ILMN_24933**	**NM_016055.4**	**0.594853628**	**1.268606525**	**0.00031550**
GOSR2	**ILMN_4229**	**NM_054022.2**	**0.593957326**	**1.346384331**	**0.00004694**
PSME2	**ILMN_19572**	**NM_002818.2**	**0.591841903**	**1.139840032**	**0.00020595**
TMEM77	**ILMN_30117**	**NM_178454.2**	**0.591390405**	**0.901345416**	**0.00467419**
PSMC6	**ILMN_3800**	**NM_002806.2**	**0.584372895**	**0.861482699**	**0.00052608**
CBX1	**ILMN_6262**	**NM_006807.3**	**0.575442923**	**1.182977034**	**0.00251133**
FLJ13614	**ILMN_12182**	**NM_139076.1**	**0.575017217**	**0.696881716**	**0.00797877**
C9orf19	**ILMN_12810**	**NM_022343.2**	**0.574894314**	**1.050523704**	**0.00532794**
CDA08	**ILMN_10328**	**NM_030790.2**	**0.574488481**	**0.835644003**	**0.00224865**
LOC84661	**ILMN_18534**	**NM_032574.2**	**0.569701867**	**0.981231913**	**0.00016403**
BCCIP	**ILMN_26223**	**NM_016567.2**	**0.569086006**	**0.844403344**	**0.00325171**
NFYC	**ILMN_5936**	**NM_014223.2**	**0.568640405**	**0.868762425**	**0.00404212**
MRE11A	**ILMN_6718**	**NM_005590.3**	**0.568494718**	**1.529673741**	**0.00001696**
OSBPL1A	**ILMN_662**	**NM_018030.3**	**0.568215143**	**0.926351958**	**0.00047778**
LOC340061	**ILMN_18797**	**NM_198282.1**	**0.567618043**	**0.689243491**	**0.00474147**
BCKDHA	**ILMN_13270**	**NM_000709.2**	**0.566591781**	**1.179166285**	**0.00005504**
VBP1	**ILMN_9083**	**NM_003372.4**	**0.565843822**	**1.066690264**	**0.00041695**
TERF2	**ILMN_21134**	**NM_005652.2**	**0.564508109**	**0.831878711**	**0.00245700**
C2orf7	**ILMN_24250**	**NM_032319.1**	**0.563716251**	**1.367309251**	**0.00042654**
CEP63	**ILMN_1690**	**NM_025180.2**	**0.558482462**	**0.742866444**	**0.00729568**
OACT5	**ILMN_22717**	**NM_005768.5**	**0.557772544**	**1.167585684**	**0.00017228**
ARF3	**ILMN_6605**	**NM_001659.1**	**0.556975803**	**0.902342007**	**0.00136220**
SLC38A6	**ILMN_7226**	**NM_153811.1**	**0.556810899**	**0.810893343**	**0.00283712**
PFN1	**ILMN_2354**	**NM_005022.2**	**0.556212216**	**0.840582789**	**0.00181308**
MANEA	**ILMN_6991**	**NM_024641.2**	**0.555690029**	**0.765970605**	**0.00902237**
ALG8	**ILMN_24209**	**NM_001007027.1**	**0.551946917**	**0.739794606**	**0.00810565**
TMEM14C	**ILMN_12206**	**NM_016462.2**	**0.551640294**	**0.959306211**	**0.00374177**

PXMP4	ILMN_4808	NM_007238.3	0.549666885	0.885829412	0.00252578
ACTR10	ILMN_18205	NM_018477.1	0.548316623	1.387712534	0.00001911
C11orf17	ILMN_9655	NM_020642.2	0.547234077	0.980450203	0.00333597
ACTR2	ILMN_2152	NM_001005386.1	0.546355357	0.812154576	0.00608161
RSNL2	ILMN_17216	NM_024692.3	0.545946152	0.829756456	0.00805801
COPZ1	ILMN_4037	NM_016057.1	0.545520981	0.799296562	0.00395313
CAPNS1	ILMN_6617	NM_001003962.1	0.54537447	0.669326948	0.00573710
STUB1	ILMN_22822	NM_005861.2	0.542125275	0.698526637	0.00491662
PSMB5	ILMN_29312	NM_002797.2	0.541998408	1.055086186	0.00029673
DYM	ILMN_6843	NM_017653.2	0.541041599	0.9654535	0.00428592
AGPS	ILMN_138634	NM_003659.1	0.540726523	1.18173707	0.00074260
CLCN3	ILMN_15279	NM_001829.2	0.540459483	0.692590724	0.00838864
ARL5	ILMN_137185	NM_012097.2	0.539235483	0.946302784	0.00202584
C4orf13	ILMN_11413	NM_001029998.1	0.538921014	1.019956729	0.00184603
CD244	ILMN_7885	NM_016382.2	0.537414131	0.706077057	0.00822767
MRPS28	ILMN_16611	NM_014018.2	0.533530893	0.932362259	0.00474327
SCCPDH	ILMN_30353	NM_016002.2	0.53299596	0.874953099	0.00267603
CSNK1A1	ILMN_24977	NM_001892.4	0.532411953	0.950426926	0.00968292
VPS29	ILMN_14188	NM_016226.2	0.529279832	1.141988392	0.00123260
C10orf7	ILMN_11650	NM_006023.1	0.529032709	1.472693353	0.00005202
INPP5B	ILMN_6646	NM_005540.1	0.528913697	0.676301308	0.00964580
ZMPSTE24	ILMN_13713	NM_005857.2	0.526380447	0.915873674	0.00174190
GALNT4	ILMN_15303	NM_003774.3	0.52596861	0.692989685	0.00942236
GOLGA5	ILMN_20157	NM_005113.2	0.52402571	0.855780518	0.00358645
GLE1L	ILMN_19199	NM_001003722.1	0.522235331	0.816909203	0.00632145
OGFOD1	ILMN_16561	NM_001031707.1	0.518441492	0.794727583	0.00303007
RBM18	ILMN_8277	NM_033117.2	0.51592074	1.116793418	0.00560431
NS4ATP2	ILMN_18384	NM_024632.3	0.515324206	0.691190711	0.00994818
PDCD6	ILMN_15265	NM_013232.2	0.514233262	1.102864386	0.00112019
GABARAPL2	ILMN_9805	NM_007285.6	0.513667927	1.671080501	0.00019022
BTF3L4	ILMN_3105	NM_152265.1	0.513640416	1.15768482	0.00001829

WIBG	ILMN_19902	NM_032345.1	0.513365324	0.802261004	0.00264002
NFE2L1	ILMN_13955	NM_003204.1	0.513152066	0.91593502	0.00041556
C7orf28A	ILMN_2087	NM_015622.5	0.509415546	0.604216727	0.00817957
C15orf17	ILMN_13536	NM_020447.2	0.507043484	1.129601563	0.00279722
TPK1	ILMN_6467	NM_022445.2	0.507032195	0.967616723	0.00125616
RHOA	ILMN_6247	NM_001664.2	0.506140959	0.748492876	0.00098190
WAS	ILMN_1668	NM_000377.1	0.504612066	1.161072475	0.00088430
LOC285636	ILMN_8228	NM_175921.4	0.503547058	0.796804283	0.00655570
TMCO1	ILMN_8084	NM_019026.2	0.502782404	0.920191132	0.00070381
TM2D1	ILMN_9063	NM_032027.2	0.502048373	0.925561389	0.00162488
RCN1	ILMN_8159	NM_002901.1	0.501933318	0.87855392	0.00039560
SFRS2	ILMN_26358	NM_003016.2	-0.501101485	0.751488215	0.00771185
NCLN	ILMN_29505	NM_020170.3	-0.50139972	1.156881313	0.00344346
MTMR2	ILMN_24002	NM_201281.1	-0.501808986	0.965612981	0.00278489
OSBPL7	ILMN_4611	NM_145798.2	-0.506236884	0.692412796	0.00713701
RIPK5	ILMN_1333	NM_199462.1	-0.506241651	1.10939465	0.00094333
ELK1	ILMN_14942	NM_005229.2	-0.506595063	1.06771036	0.00253708
ILKAP	ILMN_12973	NM_030768.2	-0.507099782	1.203991038	0.00132750
FBL	ILMN_14351	NM_001436.2	-0.508152926	0.93084529	0.00616627
RAFTLIN	ILMN_29470	NM_015150.1	-0.509860651	0.691386978	0.00867436
FEM1A	ILMN_2838	NM_018708.1	-0.51131736	0.952052307	0.00125013
PSCD2	ILMN_16003	NM_017457.3	-0.511819719	0.700114811	0.00583446
CCDC16	ILMN_23839	NM_052857.2	-0.512078285	1.0515164	0.00018637
RPL37	ILMN_138392	NM_000997.3	-0.512921504	1.018252708	0.00011698
WDR19	ILMN_11749	NM_025132.3	-0.513291141	0.791396744	0.00223438
ZCCHC3	ILMN_28604	NM_033089.6	-0.51345165	1.029007424	0.00137729
COG1	ILMN_137022	NM_018714.1	-0.513593241	0.857290291	0.00279936
MXD4	ILMN_26701	NM_006454.2	-0.513605477	0.955769313	0.00302540
WBP1	ILMN_20957	NM_012477.2	-0.514893469	0.923870702	0.00079940
LIMD2	ILMN_25849	NM_030576.2	-0.515598945	0.917797589	0.00442454
ZFYVE20	ILMN_15619	NM_022340.2	-0.517706349	1.009276209	0.00012700

MRPL2	ILMN_4811	NM_015950.3	-0.518147351	1.087524189	0.00269727
EDG4	ILMN_4660	NM_004720.4	-0.518490806	0.84209368	0.00080746
RINT-1	ILMN_23747	NM_021930.3	-0.51926901	0.819352789	0.00654352
COX6C	ILMN_6419	NM_004374.2	-0.519912949	0.799991141	0.00073464
2'-PDE	ILMN_16583	NM_177966.4	-0.521811264	1.020236026	0.00115644
SIAHBP1	ILMN_14897	NM_014281.3	-0.521985439	1.165205236	0.00404616
FLJ13149	ILMN_10466	NM_021826.4	-0.522670012	0.899495902	0.00365177
RPA3	ILMN_29134	NM_002947.3	-0.523175111	0.784664531	0.00127759
FLJ20186	ILMN_2786	NM_017702.2	-0.525049178	0.640859744	0.00639750
C14orf156	ILMN_6302	NM_031210.3	-0.525876549	0.695348323	0.00156890
YARS	ILMN_13503	NM_003680.2	-0.526900543	0.781042911	0.00307002
C10orf28	ILMN_989	NM_014472.3	-0.528865117	0.666665125	0.00893320
GNL3	ILMN_18645	NM_014366.4	-0.529243563	1.027555574	0.00638245
CIRH1A	ILMN_2574	NM_032830.1	-0.52990755	1.010140713	0.00537304
C14orf43	ILMN_22813	NM_194278.2	-0.530803886	0.832143247	0.00164632
PRPS1	ILMN_6887	NM_002764.2	-0.531046429	1.110198744	0.00014613
PUSL1	ILMN_8805	NM_153339.1	-0.53137201	0.983409622	0.00171481
PARP6	ILMN_5118	NM_020213.1	-0.532217352	1.786552893	0.00004720
NIFUN	ILMN_1909	NM_014301.2	-0.532831492	0.855300668	0.00545087
EDEM1	ILMN_9408	NM_014674.1	-0.534159313	0.899573452	0.00834955
RPL23A	ILMN_137509	NM_000984.4	-0.534879307	1.085682379	0.00146829
CCNB1IP1	ILMN_16420	NM_021178.2	-0.534921378	1.211904411	0.00007463
BMS1L	ILMN_4307	NM_014753.2	-0.535113173	0.735214469	0.00860805
MRPS24	ILMN_17184	NM_032014.1	-0.535266097	0.647342605	0.00313202
ZCCHC11	ILMN_4936	NM_001009881.1	-0.53789923	0.680978374	0.00392385
GLCCI1	ILMN_22544	NM_138426.1	-0.538041773	0.782651606	0.00866811
ZNF211	ILMN_8413	NM_006385.2	-0.538417821	0.939940258	0.00064505
TIMM22	ILMN_29380	NM_013337.2	-0.541037298	0.849931579	0.00247792
SLC41A3	ILMN_19829	NM_001008485.1	-0.541342697	0.866825731	0.00238013
SSSCA1	ILMN_7443	NM_006396.1	-0.542120495	0.791636209	0.00711843
NUP205	ILMN_11753	NM_015135.1	-0.542554722	0.903764833	0.00077945

IFT122	ILMN_21045	NM_018262.2	-0.54286388	1.090504474	0.00207521
LOC113444	ILMN_15613	NM_138428.2	-0.544285305	0.982416486	0.00067315
DNAJB6	ILMN_26714	NM_005494.2	-0.545564895	0.793932289	0.00764188
SLC25A17	ILMN_21822	NM_006358.2	-0.545698652	0.777571931	0.00476313
ZNF350	ILMN_20125	NM_021632.3	-0.550047933	0.783806276	0.00107854
RRAS2	ILMN_30083	NM_012250.3	-0.550217999	0.683428196	0.00715362
ARHGAP4	ILMN_2811	NM_001666.2	-0.551004947	0.785065836	0.00558225
PHLPPL	ILMN_10066	NM_015020.2	-0.553043887	0.955468838	0.00637876
NOL8	ILMN_25734	NM_017948.4	-0.553410288	0.6366454	0.00843367
CHD6	ILMN_28663	NM_032221.3	-0.553971876	1.023392347	0.00198387
CERK	ILMN_2275	NM_182661.1	-0.555520585	0.893010898	0.00316698
NEIL2	ILMN_11540	NM_145043.1	-0.555531793	0.732799123	0.00484798
ASCC3	ILMN_5802	NM_022091.3	-0.555810381	1.059056206	0.00552599
HECA	ILMN_3008	NM_016217.2	-0.556347682	0.858807643	0.00114314
TNRC6B	ILMN_901	NM_001024843.1	-0.556756071	0.808217912	0.00212181
ATP5I	ILMN_14284	NM_007100.2	-0.558678699	0.581710782	0.00508938
NIP7	ILMN_28300	NM_016101.3	-0.559420708	0.931442207	0.00178296
SSR4	ILMN_3302	NM_006280.1	-0.560086611	0.812498017	0.00075741
KCTD9	ILMN_1204	NM_017634.2	-0.56103139	0.901177541	0.00096362
C8orf38	ILMN_13088	NM_152416.1	-0.561399528	0.986388729	0.00117088
UBN1	ILMN_15680	NM_016936.2	-0.561640796	1.047767493	0.00032824
LOC91689	ILMN_9476	NM_033318.3	-0.562517452	1.023424687	0.00103510
C9orf156	ILMN_12842	NM_016481.2	-0.563566234	0.721816847	0.00861659
HPS1	ILMN_2724	NM_182639.1	-0.564267898	0.809408141	0.00092401
POLR2I	ILMN_17223	NM_006233.4	-0.564447269	0.610663494	0.00922027
IMMP2L	ILMN_30049	NM_032549.1	-0.564730086	0.802043738	0.00460118
LOC168850	ILMN_24570	NM_176814.3	-0.56555576	0.788592126	0.00749700
DDA1	ILMN_29105	NM_024050.4	-0.565916143	0.900027059	0.00038974
MED9	ILMN_19228	NM_018019.2	-0.569409453	0.708003815	0.00188291
WDR75	ILMN_14393	NM_032168.1	-0.5700052	0.7651042	0.00359337
MRPL11	ILMN_1149	NM_016050.2	-0.570694775	0.865336827	0.00253789

LRP5L	ILMN_650	NM_182492.1	-0.571430915	0.921914449	0.00039107
PIK4CB	ILMN_21393	NM_002651.1	-0.571689076	0.741826102	0.00464515
KRT10	ILMN_1890	NM_000421.2	-0.572172093	0.728055479	0.00436014
ZNF263	ILMN_13327	NM_005741.3	-0.575435129	0.969416741	0.00028422
BCL2	ILMN_3868	NM_000633.2	-0.575548572	0.844473447	0.00325378
PIGT	ILMN_19969	NM_015937.2	-0.576433969	1.161501121	0.00048774
INOC1	ILMN_5093	NM_032196.3	-0.576917795	0.755514595	0.00583012
SFRS12	ILMN_8967	NM_139168.1	-0.580161358	0.658939464	0.00926378
IXL	ILMN_10710	NM_017592.1	-0.580304926	0.950420241	0.00014253
C14orf138	ILMN_29130	NM_024558.1	-0.582713642	0.960895284	0.00335093
SFRS8	ILMN_9797	NM_004592.2	-0.585074071	1.347585696	0.00022540
SYNCRIP	ILMN_28470	NM_006372.3	-0.585172097	1.087116066	0.00311096
RPS19BP1	ILMN_8107	NM_194326.2	-0.585338721	0.845933847	0.00365128
MAP4K5	ILMN_10246	NM_006575.3	-0.58540163	0.829097844	0.00757634
SUV420H1	ILMN_29861	NM_016028.4	-0.58748649	1.378314577	0.00035114
C1orf35	ILMN_28904	NM_024319.2	-0.587897505	0.957215804	0.00130979
ZBTB20	ILMN_27339	NM_015642.2	-0.588916852	0.772194348	0.00270763
GATS	ILMN_18755	NM_178831.4	-0.589908263	0.711683709	0.00449993
PMPCA	ILMN_21420	NM_015160.1	-0.590865296	1.347090479	0.00013962
DDX27	ILMN_20732	NM_017895.6	-0.590934524	0.712906638	0.00573165
KPNA4	ILMN_21107	NM_002268.3	-0.591097414	0.764231033	0.00709542
LRIG2	ILMN_7215	NM_014813.1	-0.591763549	1.000300389	0.00098919
ADNP	ILMN_19803	NM_181442.1	-0.593035819	1.062015792	0.00007631
ZDHHC5	ILMN_28354	NM_015457.2	-0.594136352	0.973921694	0.00091996
BAP1	ILMN_17024	NM_004656.2	-0.594422282	1.015586606	0.00133679
TATDN2	ILMN_8206	NM_014760.2	-0.594767207	1.201800357	0.00004238
AZIN1	ILMN_4825	NM_015878.4	-0.595459472	0.919861629	0.00489867
RPS6KB1	ILMN_1872	NM_003161.2	-0.595631327	1.004764715	0.00628176
AZI1	ILMN_3856	NM_014984.1	-0.596227296	1.182177661	0.00048784
LRRC8A	ILMN_18635	NM_019594.2	-0.596405887	0.885748941	0.00277366
RBM13	ILMN_18111	NM_032509.2	-0.596437358	0.973597748	0.00014306

ZNF26	**ILMN_3233**	**NM_019591.2**	**-0.59830862**	**0.853992606**	**0.00339823**
KDELC2	**ILMN_23677**	**NM_153705.2**	**-0.600616412**	**1.052805945**	**0.00022644**
KIAA1530	**ILMN_958**	**NM_020894.1**	**-0.602437968**	**1.261110975**	**0.00010936**
FAU	**ILMN_3073**	**NM_001997.3**	**-0.602457622**	**1.036010219**	**0.00001407**
C8orf33	**ILMN_15901**	**NM_023080.1**	**-0.603252646**	**1.414514982**	**0.00026532**
SEMA4D	**ILMN_24615**	**NM_006378.2**	**-0.603394443**	**0.861269484**	**0.00139078**
DKFZp761I2123	**ILMN_4684**	**NM_031449.3**	**-0.604000476**	**0.903562568**	**0.00721373**
KIAA1542	**ILMN_22314**	**NM_020901.1**	**-0.604016318**	**0.830834961**	**0.00079312**
NARF	**ILMN_138328**	**NM_031968.1**	**-0.60417202**	**0.883103921**	**0.00757037**
CRAMP1L	**ILMN_12672**	**NM_020825.2**	**-0.604341887**	**1.164221816**	**0.00019481**
RASA2	**ILMN_15489**	**NM_006506.2**	**-0.605237893**	**0.88103833**	**0.00727633**
IL12A	**ILMN_28240**	**NM_000882.2**	**-0.605767267**	**0.706673327**	**0.00764865**
RNPEPL1	**ILMN_18851**	**NM_018226.3**	**-0.605977149**	**0.907204305**	**0.00144612**
C3orf19	**ILMN_13337**	**NM_016474.3**	**-0.606982737**	**0.935698555**	**0.00150798**
TSSC4	**ILMN_17177**	**NM_005706.2**	**-0.607164855**	**1.028524623**	**0.00063006**
DOCK10	**ILMN_15188**	**NM_014689.1**	**-0.610185033**	**0.927657427**	**0.00070649**
QRICH1	**ILMN_22436**	**NM_017730.2**	**-0.611913915**	**1.527494986**	**0.00015097**
ZNF622	**ILMN_1143**	**NM_033414.2**	**-0.613218062**	**1.414248597**	**0.00002261**
ZNF416	**ILMN_13535**	**NM_017879.1**	**-0.613628233**	**0.700755649**	**0.00915879**
PDRG1	**ILMN_20546**	**NM_030815.2**	**-0.614441842**	**1.064193414**	**0.00435025**
C20orf3	**ILMN_2205**	**NM_020531.2**	**-0.614571123**	**0.768684462**	**0.00967519**
ZNF529	**ILMN_24517**	**NM_020951.1**	**-0.614692228**	**0.785446095**	**0.00106414**
TP53I13	**ILMN_23391**	**NM_138349.2**	**-0.616381148**	**0.729544257**	**0.00769178**
MBTPS1	**ILMN_5443**	**NM_201268.1**	**-0.617123097**	**0.888166037**	**0.00240723**
TROVE2	**ILMN_4319**	**NM_004600.3**	**-0.617753128**	**0.673186701**	**0.00774056**
TUBGCP5	**ILMN_25939**	**NM_052903.2**	**-0.617898874**	**0.889360861**	**0.00042017**
MRPL55	**ILMN_467**	**NM_181464.1**	**-0.618983592**	**0.811107318**	**0.00119417**
CUL2	**ILMN_26940**	**NM_003591.2**	**-0.61974505**	**1.127957039**	**0.00054435**
ZNF76	**ILMN_6486**	**NM_003427.3**	**-0.620226015**	**1.171212418**	**0.00092501**
C4orf9	**ILMN_10925**	**NM_003703.1**	**-0.620535032**	**1.215629606**	**0.00038208**
KIAA1958	**ILMN_17353**	**NM_133465.1**	**-0.621349152**	**0.972702081**	**0.00872690**

TAF1C	**ILMN_4122**	**NM_139353.1**	**-0.622990892**	**1.445562625**	**0.00002040**
IDS	**ILMN_2023**	**NM_000202.2**	**-0.62329983**	**0.900843185**	**0.00072718**
PRAF1	**ILMN_2978**	**NM_022490.1**	**-0.623820785**	**1.032997273**	**0.00096113**
C20orf29	**ILMN_7561**	**NM_018347.1**	**-0.624761404**	**1.125534915**	**0.00062872**
GATAD2A	**ILMN_1801**	**NM_017660.2**	**-0.625589049**	**1.147481763**	**0.00011571**
ACACB	**ILMN_3318**	**NM_001093.2**	**-0.626024408**	**1.038757802**	**0.00759206**
ARHGDIA	**ILMN_15874**	**NM_004309.3**	**-0.628056706**	**0.95971498**	**0.00078607**
RBKS	**ILMN_21205**	**NM_022128.1**	**-0.629280777**	**1.243196703**	**0.00029184**
GMEB1	**ILMN_14071**	**NM_006582.2**	**-0.630458154**	**1.010146742**	**0.00544840**
KIAA0179	**ILMN_14288**	**NM_015056.1**	**-0.631285034**	**0.926908086**	**0.00071174**
C17orf48	**ILMN_6506**	**NM_020233.3**	**-0.631507612**	**1.435374244**	**0.00003101**
HNRPL	**ILMN_5928**	**NM_001533.2**	**-0.631636976**	**0.705438767**	**0.00325831**
PTPN1	**ILMN_5417**	**NM_002827.2**	**-0.633813993**	**0.776627472**	**0.00670939**
IRF2BP1	**ILMN_6522**	**NM_015649.1**	**-0.635292951**	**0.890038121**	**0.00457907**
WBSCR22	**ILMN_26330**	**NM_017528.2**	**-0.636188587**	**0.878022756**	**0.00081436**
LOC91431	**ILMN_26207**	**NM_138698.2**	**-0.63820878**	**0.657348609**	**0.00926809**
PAQR3	**ILMN_20371**	**NM_177453.2**	**-0.640215369**	**0.683823498**	**0.00569143**
HSF2	**ILMN_3760**	**NM_004506.2**	**-0.640864075**	**0.758861543**	**0.00229860**
GUK1	**ILMN_18191**	**NM_000858.4**	**-0.640890663**	**1.421767573**	**0.00002100**
BRF2	**ILMN_2966**	**NM_018310.2**	**-0.641792347**	**0.712970641**	**0.00941033**
LOC440348	**ILMN_12371**	**NM_001018059.1**	**-0.643288377**	**0.836871612**	**0.00364941**
PRRT3	**ILMN_19313**	**NM_207351.2**	**-0.644537989**	**0.90258133**	**0.00073831**
H1FX	**ILMN_26614**	**NM_006026.2**	**-0.645743275**	**0.726455223**	**0.00562703**
HSPA5	**ILMN_16546**	**NM_005347.2**	**-0.645854429**	**1.044504608**	**0.00076007**
USP7	**ILMN_3791**	**NM_003470.1**	**-0.645861103**	**0.765032434**	**0.00861637**
RAGE	**ILMN_5109**	**NM_014226.1**	**-0.646248862**	**0.816833521**	**0.00096010**
HEAB	**ILMN_17235**	**NM_006831.1**	**-0.647356002**	**0.95592989**	**0.00728557**
CCDC59	**ILMN_12564**	**NM_014167.1**	**-0.649844073**	**0.844038281**	**0.00199060**
UBE2B	**ILMN_19013**	**NM_003337.2**	**-0.650171493**	**0.856337935**	**0.00054149**
TMEM1	**ILMN_9744**	**NM_003274.3**	**-0.650272701**	**1.006083249**	**0.00003470**
CXXC5	**ILMN_10449**	**NM_016463.5**	**-0.650664893**	**0.600426859**	**0.00854733**

SRRM1	ILMN_2484	NM_005839.2	-0.650793883	1.344938502	0.00013658
SFRS3	ILMN_29649	NM_003017.3	-0.653578545	0.999498208	0.00997300
PDCL3	ILMN_30010	NM_024065.3	-0.654245679	0.941531099	0.00056039
PRR3	ILMN_21022	NM_025263.1	-0.654275478	1.237039594	0.00302803
BTAF1	ILMN_8616	NM_003972.2	-0.655041764	2.000675247	0.00000148
LAS1L	ILMN_10798	NM_031206.2	-0.655593111	1.230989934	0.00176207
SLC6A16	ILMN_10688	NM_014037.2	-0.656717923	0.83343565	0.00328586
PIM2	ILMN_27932	NM_006875.2	-0.657356732	0.93398096	0.00120534
KIAA1600	ILMN_8082	NM_020940.2	-0.657609423	0.858231585	0.00665377
EXOSC2	ILMN_11049	NM_014285.4	-0.658469055	0.89439764	0.00137281
C6orf149	ILMN_18141	NM_020408.3	-0.658551633	0.968041299	0.00207399
NUDT16L1	ILMN_20270	NM_032349.2	-0.658754939	1.000965634	0.00011280
PQLC1	ILMN_5626	NM_025078.3	-0.658826711	1.46688705	0.00003286
TMC6	ILMN_19035	NM_007267.5	-0.659317538	0.988841774	0.00146505
SH3BP4	ILMN_138674	NM_014521.1	-0.659423978	1.080747687	0.00307405
C21orf66	ILMN_15558	NM_013329.2	-0.660169817	1.360744855	0.00011543
RPL26	ILMN_139337	NM_000987.2	-0.660388215	0.838265035	0.00102103
JMJD2B	ILMN_2379	NM_015015.1	-0.660517636	0.764706358	0.00767484
ZNF431	ILMN_7008	NM_133473.1	-0.660741637	0.982149669	0.00093699
DGCR14	ILMN_694	NM_022719.1	-0.661198685	0.947293749	0.00241510
TEF	ILMN_7440	NM_003216.2	-0.66136378	1.177478992	0.00011265
POLE	ILMN_18431	NM_006231.2	-0.662672826	0.871053439	0.00362261
CRKRS	ILMN_9786	NM_016507.1	-0.663155806	1.024487964	0.00038030
LMNB2	ILMN_24712	NM_032737.2	-0.663422716	0.819620917	0.00522630
PPAN	ILMN_25948	NM_020230.4	-0.663459334	0.811996002	0.00656122
TRAF6	ILMN_8846	NM_004620.2	-0.664832171	1.183856973	0.00145782
ZNF238	ILMN_5128	NM_205768.1	-0.66538502	1.063282	0.00121218
DIP13B	ILMN_139404	NM_018171.2	-0.665918599	0.816448496	0.00182992
C1orf122	ILMN_26311	NM_198446.1	-0.667704514	0.758544159	0.00554261
WDR79	ILMN_1814	NM_018081.1	-0.66887631	1.103656742	0.00682748
ZNF75A	ILMN_3468	NM_153028.1	-0.668953048	0.77004373	0.00387346

EBPL	ILMN_18169	NM_032565.1	-0.669087401	0.948622616	0.00047601
SUV39H1	ILMN_6133	NM_003173.1	-0.670194855	0.975895869	0.00028897
MORC4	ILMN_5233	NM_024657.2	-0.670720191	1.081812244	0.00034502
RHOT2	ILMN_21353	NM_138769.1	-0.67287188	0.718635543	0.00588322
LSM5	ILMN_17896	NM_012322.1	-0.673056964	1.1919698	0.00001152
ZNF35	ILMN_15315	NM_003420.2	-0.676575556	1.219238883	0.00088914
IHPK1	ILMN_1661	NM_153273.3	-0.677703526	0.844737234	0.00342912
NOLA1	ILMN_137056	NM_032993.1	-0.677952037	1.201625866	0.00010415
ZNF545	ILMN_15084	NM_133466.1	-0.678360671	0.857507765	0.00077872
SCMH1	ILMN_5778	NM_001031694.1	-0.678476927	1.088681731	0.00117579
XPO4	ILMN_11492	NM_022459.3	-0.678501648	1.075202138	0.00048775
CCDC69	ILMN_12667	NM_015621.2	-0.680078881	0.782969982	0.00618847
CALM1	ILMN_137303	NM_006888.2	-0.680552658	0.845807834	0.00246513
C21orf57	ILMN_21121	NM_058181.1	-0.683310928	0.78631219	0.00469043
RANBP3	ILMN_27457	NM_003624.1	-0.683827258	0.90891312	0.00063720
ZNF142	ILMN_11618	NM_005081.2	-0.684025915	1.286496115	0.00001617
CBLL1	ILMN_24021	NM_024814.1	-0.68419063	0.883478823	0.00615600
OTUD5	ILMN_8231	NM_017602.2	-0.684681416	0.824341727	0.00517850
C14orf4	ILMN_23475	NM_024496.2	-0.684736533	1.011836565	0.00048207
PP2447	ILMN_25172	NM_025204.2	-0.684755437	1.569550738	0.00005516
USP14	ILMN_137727	NM_005151.2	-0.684905707	0.865550813	0.00568072
SEC61B	ILMN_446	NM_006808.2	-0.687486258	0.670776632	0.00406423
RNF126	ILMN_5287	NM_194460.1	-0.68765419	1.014174257	0.00203408
OFD1	ILMN_10088	NM_003611.1	-0.687787909	0.825117046	0.00356926
RENT1	ILMN_138046	NM_002911.2	-0.687855528	0.699664811	0.00739681
ZNF17	ILMN_13772	NM_006959.1	-0.688040083	0.670592008	0.00734045
UBR1	ILMN_137462	NM_174916.1	-0.688734037	1.016853169	0.00193488
LTBP4	ILMN_21051	NM_003573.1	-0.690128055	0.744259013	0.00665099
DNMT3A	ILMN_26218	NM_175630.1	-0.690260033	1.570643653	0.00000747
GNL1	ILMN_3133	NM_005275.2	-0.691657668	1.02612133	0.00132046
ARID3B	ILMN_4032	NM_006465.1	-0.691793692	0.977354302	0.00033123

LEPROTL1	**ILMN_4515**	**NM_015344.1**	**-0.691937884**	**0.806940218**	**0.00299727**
GRPEL1	**ILMN_7749**	**NM_025196.2**	**-0.692082354**	**1.001336975**	**0.00070527**
CSGlcA-T	**ILMN_21838**	**NM_019015.1**	**-0.694221557**	**1.127669784**	**0.00026213**
GCC1	**ILMN_21168**	**NM_024523.5**	**-0.6951262**	**1.366586368**	**0.00003783**
RAD23B	**ILMN_19346**	**NM_002874.3**	**-0.695226728**	**1.102097779**	**0.00406715**
LOC93081	**ILMN_8559**	**NM_138779.2**	**-0.695301754**	**0.760850692**	**0.00280824**
FLJ10374	**ILMN_3627**	**NM_018074.3**	**-0.695464213**	**1.079974637**	**0.00457030**
C17orf53	**ILMN_17950**	**NM_024032.2**	**-0.696635685**	**1.255525354**	**0.00366275**
NCK2	**ILMN_8369**	**NM_003581.2**	**-0.696808439**	**0.886780127**	**0.00135857**
C20orf52	**ILMN_15797**	**NM_080748.1**	**-0.699212306**	**0.622463785**	**0.00379560**
MLF1IP	**ILMN_16700**	**NM_024629.2**	**-0.699384768**	**0.862592142**	**0.00813547**
ZNF549	**ILMN_12970**	**NM_153263.1**	**-0.699401199**	**0.741164155**	**0.00389691**
CLYBL	**ILMN_27821**	**NM_206808.1**	**-0.699524111**	**1.250909264**	**0.00053328**
ZNF136	**ILMN_7813**	**NM_003437.2**	**-0.700419265**	**0.833346179**	**0.00189626**
STXBP5	**ILMN_3767**	**NM_139244.2**	**-0.704314781**	**1.232465712**	**0.00019410**
ALG9	**ILMN_22873**	**NM_024740.1**	**-0.70462799**	**0.927910229**	**0.00503792**
ZNF426	**ILMN_13082**	**NM_024106.1**	**-0.705339858**	**0.961994526**	**0.00367733**
DENND4C	**ILMN_2455**	**NM_017925.3**	**-0.705529703**	**1.146949146**	**0.00115710**
EIF1	**ILMN_3037**	**NM_005801.3**	**-0.70553125**	**0.947529023**	**0.00032097**
KLHL21	**ILMN_14418**	**NM_014851.2**	**-0.706027126**	**0.898564478**	**0.00204385**
ANK3	**ILMN_2714**	**NM_001149.2**	**-0.706311526**	**0.911010591**	**0.00141958**
MPP6	**ILMN_28432**	**NM_016447.2**	**-0.707177805**	**0.740970318**	**0.00083022**
CCL28	**ILMN_1615**	**NM_019846.3**	**-0.708252508**	**1.273210594**	**0.00046153**
SNTA1	**ILMN_4031**	**NM_003098.2**	**-0.708334717**	**0.978312341**	**0.00055783**
TARBP1	**ILMN_14414**	**NM_005646.2**	**-0.709611231**	**0.976054013**	**0.00211269**
JARID1A	**ILMN_12150**	**NM_005056.1**	**-0.711271184**	**1.01467053**	**0.00143321**
C20orf149	**ILMN_21879**	**NM_024299.2**	**-0.711320456**	**0.814646204**	**0.00387198**
PILRB	**ILMN_1984**	**NM_175047.2**	**-0.712138848**	**0.731016965**	**0.00355462**
TERF1	**ILMN_11986**	**NM_017489.1**	**-0.712178428**	**0.990225787**	**0.00113241**
MSI2	**ILMN_525**	**NM_138962.2**	**-0.712606214**	**1.035281345**	**0.00176819**
MRPS18A	**ILMN_2508**	**NM_018135.2**	**-0.714481425**	**1.271085307**	**0.00053144**

C3orf39	**ILMN_2070**	**NM_032806.4**	**-0.714496822**	**1.136707686**	**0.00013448**
PCSK5	**ILMN_6597**	**NM_006200.2**	**-0.715939114**	**1.036876117**	**0.00035864**
DPP9	**ILMN_26244**	**NM_139159.3**	**-0.716419901**	**0.912894052**	**0.00060259**
EDD1	**ILMN_11309**	**NM_015902.4**	**-0.716696292**	**2.001580199**	**0.00003324**
C16orf7	**ILMN_693**	**NM_004913.1**	**-0.717368103**	**0.922793072**	**0.00483044**
SLC2A4RG	**ILMN_11640**	**NM_020062.3**	**-0.717939345**	**0.844967139**	**0.00210980**
SBF1	**ILMN_22729**	**NM_002972.1**	**-0.719573729**	**0.872216344**	**0.00261564**
N4BP2	**ILMN_7118**	**NM_018177.2**	**-0.720407286**	**0.643502768**	**0.00329044**
CCNK	**ILMN_4502**	**NM_003858.2**	**-0.72058077**	**1.458689467**	**0.00010225**
EHD1	**ILMN_17263**	**NM_006795.2**	**-0.720719364**	**0.766609451**	**0.00483019**
BANP	**ILMN_8638**	**NM_079837.1**	**-0.722372231**	**0.865374603**	**0.00546821**
TNIP2	**ILMN_6730**	**NM_024309.2**	**-0.72274032**	**1.019287814**	**0.00361216**
ZNF274	**ILMN_6185**	**NM_016324.2**	**-0.723234244**	**1.165667625**	**0.00001985**
STAT5B	**ILMN_16004**	**NM_012448.3**	**-0.724319615**	**0.858281253**	**0.00926193**
ASB6	**ILMN_13316**	**NM_177999.1**	**-0.72611613**	**0.921960087**	**0.00553967**
NY-SAR-48	**ILMN_2230**	**NM_001011699.1**	**-0.727033786**	**1.317919304**	**0.00086801**
U2AF1L3	**ILMN_8757**	**NM_144987.1**	**-0.72871967**	**1.092424247**	**0.00016495**
CEBPZ	**ILMN_10421**	**NM_005760.2**	**-0.72887728**	**0.967945932**	**0.00257282**
SNRPA1	**ILMN_1523**	**NM_003090.2**	**-0.729357658**	**1.157672317**	**0.00072721**
ZNF593	**ILMN_20499**	**NM_015871.2**	**-0.729711215**	**0.870749371**	**0.00144956**
DLNB14	**ILMN_6803**	**NM_198489.1**	**-0.7305056**	**1.233687869**	**0.00130973**
ACOT4	**ILMN_5812**	**NM_152331.2**	**-0.730543719**	**0.709395594**	**0.00449761**
PARC	**ILMN_14183**	**NM_015089.2**	**-0.730864807**	**0.732382684**	**0.00622436**
AYTL2	**ILMN_15076**	**NM_024830.3**	**-0.731746218**	**1.019606414**	**0.00177154**
MLL4	**ILMN_28047**	**NM_014727.1**	**-0.732461775**	**0.926478158**	**0.00075914**
ABCF1	**ILMN_6938**	**NM_001090.2**	**-0.732508988**	**1.080306669**	**0.00095497**
ATG9A	**ILMN_24497**	**NM_024085.2**	**-0.733386266**	**0.893550371**	**0.00569509**
C1orf33	**ILMN_1930**	**NM_016183.2**	**-0.735053733**	**1.273787628**	**0.00058830**
PLEKHG4	**ILMN_11658**	**NM_015432.2**	**-0.735675634**	**0.781101106**	**0.00809126**
CYLD	**ILMN_28818**	**NM_015247.1**	**-0.735700277**	**0.925754857**	**0.00099722**
ACRC	**ILMN_21976**	**NM_052957.2**	**-0.736674197**	**0.993429353**	**0.00204543**

UXT	ILMN_2795	NM_004182.2	-0.736776229	1.01746461	0.00008724
MYH3	ILMN_20953	NM_002470.1	-0.737874135	1.01897372	0.00311632
DDX39	ILMN_19976	NM_005804.2	-0.738861267	1.494534945	0.00001645
PRKCA	ILMN_24085	NM_002737.2	-0.74026775	1.830687543	0.00002155
LOC55565	ILMN_22894	NM_017530.1	-0.740330872	1.103927452	0.00127144
PARP15	ILMN_3312	NM_152615.1	-0.740519502	0.59182088	0.00922714
UBE2J1	ILMN_12301	NM_016021.2	-0.741117847	1.006081046	0.00022877
LCMT2	ILMN_7283	NM_014793.3	-0.74274383	0.744741119	0.00146696
bA16L21.2.1	ILMN_13855	NM_001015882.1	-0.742794207	0.714961107	0.00950935
ELL	ILMN_23957	NM_006532.2	-0.742801328	0.941853782	0.00238549
NARG1	ILMN_16240	NM_057175.3	-0.743022897	0.99023172	0.00691777
NELF	ILMN_28709	NM_015537.3	-0.743522541	0.667130343	0.00722468
FOXP1	ILMN_20586	NM_032682.4	-0.744323801	1.338638759	0.00012440
KBTBD2	ILMN_2507	NM_015483.1	-0.745628316	1.151680836	0.00011902
HMGCS1	ILMN_18980	NM_002130.4	-0.748106632	1.188287026	0.00046412
TAF5L	ILMN_6872	NM_014409.3	-0.749085176	0.868547839	0.00092389
RNF138	ILMN_587	NM_016271.3	-0.752277852	1.235057135	0.00009252
EID3	ILMN_22737	NM_001008394.1	-0.753211703	0.983834177	0.00177572
BTBD6	ILMN_28665	NM_033271.1	-0.753404502	1.357730493	0.00020193
ELK4	ILMN_16457	NM_021795.2	-0.755005589	0.67188118	0.00715763
TPST1	ILMN_2477	NM_003596.2	-0.755507792	1.228445923	0.00011312
RG9MTD1	ILMN_26970	NM_017819.1	-0.756251186	0.997215873	0.00046399
HYPE	ILMN_24815	NM_007076.2	-0.756371069	1.076873012	0.00190176
SNX26	ILMN_12508	NM_052948.2	-0.758065079	0.8931702	0.00074244
CAMK1G	ILMN_28359	NM_020439.2	-0.758706082	0.831151411	0.00712541
TOPORS	ILMN_20252	NM_005802.2	-0.759046826	1.053204205	0.00024076
PRSS15	ILMN_5804	NM_004793.2	-0.759266614	0.907428592	0.00120227
TUBA1	ILMN_20363	NM_006000.1	-0.760742973	0.712101421	0.00570485
RPL37A	ILMN_137134	NM_000998.3	-0.760816832	0.867875221	0.00096298
MADD	ILMN_9428	NM_130471.1	-0.763259324	0.924374443	0.00427367
MIF	ILMN_26688	NM_002415.1	-0.763324033	0.748772869	0.00266627

UBE2O	ILMN_17049	NM_022066.2	-0.763507156	1.161334399	0.00644586
FLJ44216	ILMN_16903	NM_198567.1	-0.764370213	0.90170466	0.00705292
RBJ	ILMN_30155	NM_016544.1	-0.76469689	0.978556237	0.00239779
STAT4	ILMN_8937	NM_003151.2	-0.765414901	0.907546517	0.00158587
ELK4	ILMN_7899	NM_001973.2	-0.765582106	1.085812566	0.00565791
YPEL5	ILMN_8828	NM_016061.1	-0.765651622	0.877344177	0.00894663
ZNF548	ILMN_16141	NM_152909.2	-0.766023955	0.9212858	0.00022428
VPS11	ILMN_13229	NM_021729.4	-0.766315738	1.106426752	0.00010581
SAV1	ILMN_15731	NM_021818.2	-0.767096079	0.888541282	0.00128585
ZNF447	ILMN_16020	NM_023926.3	-0.768713349	0.826659263	0.00080815
EYA3	ILMN_23590	NM_172098.1	-0.769040512	0.942443665	0.00038469
MESDC1	ILMN_18570	NM_022566.1	-0.770159479	1.08221592	0.00685104
EML4	ILMN_21821	NM_019063.2	-0.771210818	0.785648246	0.00704352
RBM12	ILMN_3104	NM_006047.4	-0.773445395	1.256818095	0.00004835
SUPT3H	ILMN_4557	NM_003599.1	-0.775989516	0.790995011	0.00654034
PELO	ILMN_19741	NM_015946.4	-0.777913817	0.828611002	0.00586600
C17orf63	ILMN_5219	NM_018182.1	-0.779026498	1.527509018	0.00000522
NFKBIA	ILMN_6745	NM_020529.1	-0.780491608	0.650591266	0.00530786
SLC20A2	ILMN_29659	NM_006749.3	-0.782979975	1.033443966	0.00285246
TDP1	ILMN_12296	NM_018319.3	-0.783501996	1.750778225	0.00003375
ZBTB25	ILMN_7193	NM_006977.2	-0.783772367	1.293682365	0.00001214
TRAPPC6A	ILMN_1174	NM_024108.1	-0.783976007	0.952296907	0.00066233
PPIE	ILMN_13734	NM_203456.1	-0.784546972	0.738368064	0.00695734
RAD54L2	ILMN_30244	NM_015106.1	-0.785402093	1.01822683	0.00082883
DKFZp434K1815	ILMN_12314	NM_152892.1	-0.786393593	1.017330098	0.00076794
SFI1	ILMN_23938	NM_014775.2	-0.786495819	1.077617495	0.00192246
GPATC4	ILMN_839	NM_182679.1	-0.787986184	1.138781761	0.00084221
SKIV2L	ILMN_646	NM_006929.4	-0.789248907	1.727502765	0.00001265
LUZP1	ILMN_2667	NM_033631.2	-0.796097071	0.967871182	0.00268820
MNT	ILMN_21283	NM_020310.2	-0.7978727	1.20884313	0.00070966
BRPF3	ILMN_8164	NM_015695.1	-0.798153286	1.272715574	0.00000760

PRDM4	**ILMN_1579**	**NM_012406.3**	**-0.79826194**	**1.253362778**	**0.00020244**
NFKBIZ	**ILMN_16362**	**NM_001005474.1**	**-0.800584648**	**0.745885878**	**0.00925134**
STAG3	**ILMN_28713**	**NM_012447.2**	**-0.801293102**	**0.803248924**	**0.00395595**
SFPQ	**ILMN_5703**	**NM_005066.1**	**-0.802153677**	**0.928784898**	**0.00036238**
TWISTNB	**ILMN_24604**	**NM_001002926.1**	**-0.802196716**	**1.019362059**	**0.00157788**
CITED4	**ILMN_15271**	**NM_133467.2**	**-0.803333157**	**0.992074124**	**0.00086738**
DOCK9	**ILMN_12914**	**NM_015296.1**	**-0.805523629**	**1.147821312**	**0.00005151**
CTRL	**ILMN_21415**	**NM_001907.1**	**-0.806071732**	**0.807620012**	**0.00331623**
UBXD4	**ILMN_23019**	**NM_181713.3**	**-0.807345562**	**0.860528095**	**0.00123436**
RALGDS	**ILMN_9432**	**NM_006266.2**	**-0.807833401**	**0.996741813**	**0.00054221**
DKFZp686L1814	**ILMN_26192**	**NM_194282.1**	**-0.808475893**	**1.037713738**	**0.00023363**
PXN	**ILMN_14932**	**NM_002859.1**	**-0.809403238**	**0.809655666**	**0.00041816**
C9orf28	**ILMN_20760**	**NM_033446.1**	**-0.809751743**	**0.803182923**	**0.00936620**
IL4R	**ILMN_17789**	**NM_000418.2**	**-0.810302045**	**0.973385568**	**0.00071234**
RPL13	**ILMN_2271**	**NM_033251.1**	**-0.810746203**	**1.137734142**	**0.00006465**
TXNDC11	**ILMN_17937**	**NM_015914.5**	**-0.810961885**	**1.01531958**	**0.00912042**
ZXDB	**ILMN_4827**	**NM_007157.3**	**-0.812239002**	**1.025037862**	**0.00016710**
ABCB1	**ILMN_7395**	**NM_000927.3**	**-0.813841941**	**0.830184605**	**0.00187186**
SLC10A3	**ILMN_4203**	**NM_019848.2**	**-0.814201516**	**0.77976641**	**0.00419819**
GFPT1	**ILMN_5797**	**NM_002056.1**	**-0.815838939**	**1.010349776**	**0.00913416**
YAF2	**ILMN_19509**	**NM_001012424.1**	**-0.817039087**	**1.544588783**	**0.00007635**
CRTC2	**ILMN_28825**	**NM_181715.1**	**-0.819154628**	**1.149987942**	**0.00102618**
GSPT1	**ILMN_5039**	**NM_002094.1**	**-0.819395045**	**1.123312625**	**0.00198255**
FLJ31204	**ILMN_14285**	**NM_174912.2**	**-0.82136343**	**1.204204377**	**0.00070829**
POMT1	**ILMN_18145**	**NM_007171.2**	**-0.821400025**	**0.961685443**	**0.00626720**
FLJ38964	**ILMN_22580**	**NM_173527.1**	**-0.821475189**	**1.096974111**	**0.00212659**
TBC1D15	**ILMN_20610**	**NM_022771.3**	**-0.821648635**	**1.158524645**	**0.00067360**
AXIN1	**ILMN_6274**	**NM_181050.1**	**-0.822583751**	**0.790015272**	**0.00511492**
FKBP11	**ILMN_14765**	**NM_016594.1**	**-0.823000352**	**0.74672709**	**0.00267008**
KIAA0961	**ILMN_26156**	**NM_014898.1**	**-0.824726521**	**1.080504235**	**0.00146065**
PLEKHG2	**ILMN_4000**	**NM_022835.1**	**-0.825611045**	**1.267875781**	**0.00079500**

C20orf11	ILMN_27220	NM_017896.2	-0.827007513	1.128744745	0.00003984
PIAS2	ILMN_11074	NM_173206.2	-0.830152143	1.456467935	0.00131011
RUNX3	ILMN_16236	NM_004350.1	-0.830408072	1.178110719	0.00021931
RAPGEF6	ILMN_6036	NM_016340.3	-0.830860715	0.719502179	0.00450501
HPS4	ILMN_5996	NM_152843.1	-0.831101249	1.850462398	0.00002616
ATP2C1	ILMN_16216	NM_014382.2	-0.8314472	1.496804384	0.00032372
MPP5	ILMN_21733	NM_022474.2	-0.831785694	1.231105814	0.00021771
EBI2	ILMN_5986	NM_004951.3	-0.832449329	0.736312502	0.00715280
CCDC58	ILMN_27140	NM_001017928.2	-0.833159452	1.090267998	0.00039098
ZNF101	ILMN_10157	NM_033204.2	-0.835053726	0.883406529	0.00125971
CCDC45	ILMN_24620	NM_138363.1	-0.83514053	1.715922417	0.00000317
DGKD	ILMN_2079	NM_152879.2	-0.837081703	1.214349576	0.00006287
JUND	ILMN_29325	NM_005354.2	-0.839529854	1.44576351	0.00000068
CALR	ILMN_18909	NM_004343.2	-0.840122952	0.980075778	0.00025069
ASNS	ILMN_14195	NM_133436.1	-0.840850315	1.139496001	0.00001648
IPO4	ILMN_26191	NM_024658.3	-0.841511864	1.222419638	0.00002488
MOAP1	ILMN_10935	NM_022151.4	-0.841645123	1.343199607	0.00008726
MGC40579	ILMN_6451	NM_152776.1	-0.842645626	0.934865684	0.00247629
ERCC5	ILMN_13388	NM_000123.2	-0.843937201	1.129758717	0.00031986
CIB1	ILMN_11794	NM_006384.2	-0.844079627	1.058491226	0.00150153
C14orf169	ILMN_4108	NM_024644.1	-0.845960892	1.269502867	0.00002600
ALDOC	ILMN_15767	NM_005165.2	-0.845999485	1.06749991	0.00086194
LOC56902	ILMN_26243	NM_020143.2	-0.846100126	0.754187475	0.00240439
RRM1	ILMN_14789	NM_001033.2	-0.846190811	0.88356385	0.00601741
C14orf32	ILMN_24999	NM_144578.2	-0.8467791	1.110496493	0.00648758
ZNF484	ILMN_2513	NM_031486.1	-0.847260349	0.983148562	0.00087595
DNAJB2	ILMN_137328	NM_006736.4	-0.847437659	1.454772657	0.00000649
SBDS	ILMN_15766	NM_016038.2	-0.847947777	0.599100733	0.00910234
LITAF	ILMN_7898	NM_004862.2	-0.848079954	1.064922674	0.00250002
SNRK	ILMN_5234	NM_017719.3	-0.849034935	1.225928527	0.00032831
DPP4	ILMN_20248	NM_001935.3	-0.849217133	0.77184925	0.00411250

TFRC	ILMN_12909	NM_003234.1	-0.84942199	1.14393614	0.00014138
CACNA2D2	ILMN_15989	NM_001005505.1	-0.849869058	0.79755057	0.00438546
MAP6D1	ILMN_7455	NM_024871.1	-0.85096354	1.037983445	0.00367303
MGC4728	ILMN_1981	NM_198542.1	-0.851368338	1.04653516	0.00004789
HSPC176	ILMN_26849	NM_016209.1	-0.852041704	1.178838591	0.00001505
DNAJB1	ILMN_19740	NM_006145.1	-0.852508634	0.85068799	0.00581678
COCH	ILMN_7401	NM_004086.1	-0.853301185	1.006291485	0.00224105
ST6GALNAC4	ILMN_137977	NM_175040.1	-0.854315521	1.779605121	0.00000418
FOXP1	ILMN_21389	NM_001012505.1	-0.854606153	1.423320073	0.00019417
ZNF324	ILMN_29920	NM_014347.1	-0.856946591	0.781397595	0.00557212
RPS7	ILMN_10275	NM_001011.3	-0.856988557	0.802701927	0.00150351
ZBED4	ILMN_8641	NM_014838.1	-0.859713393	1.523638248	0.00003818
ZFP42	ILMN_137432	NM_174900.2	-0.859725141	1.19719526	0.00295165
POLR1C	ILMN_26638	NM_203290.1	-0.860212976	1.192204871	0.00013691
GARNL1	ILMN_2823	NM_194301.2	-0.861956735	1.353019331	0.00032433
LANCL2	ILMN_920	NM_018697.3	-0.862500848	1.173455357	0.00105055
TPM2	ILMN_22618	NM_213674.1	-0.862570029	0.757707188	0.00573649
LGMN	ILMN_20242	NM_005606.5	-0.863810044	0.698299435	0.00748695
RAB11FIP3	ILMN_7754	NM_014700.2	-0.864253745	1.039540889	0.00011007
PWP2H	ILMN_28098	NM_005049.2	-0.864825833	1.15514266	0.00041590
JAM3	ILMN_25295	NM_032801.3	-0.866648014	0.642975938	0.00684587
SRRM2	ILMN_21088	NM_016333.2	-0.866980414	0.656284143	0.00615188
CHD1	ILMN_27142	NM_001270.2	-0.867782711	0.915916348	0.00428004
DRD3	ILMN_137372	NM_033663.2	-0.868703905	0.749723692	0.00579986
ASCC3L1	ILMN_18834	NM_014014.2	-0.868807333	1.377643623	0.00001703
MGMT	ILMN_18705	NM_002412.2	-0.869575292	0.843019505	0.00366941
NFKBIB	ILMN_8370	NM_001001716.1	-0.871643725	1.022728781	0.00128423
TCP11L2	ILMN_21615	NM_152772.1	-0.872360842	0.87759018	0.00602482
MARCH6	ILMN_2629	NM_005885.2	-0.874989813	0.996225291	0.00361251
NARG1L	ILMN_9228	NM_018527.2	-0.875950484	1.25430275	0.00011058
FYN	ILMN_5919	NM_002037.3	-0.877027664	1.138050737	0.00037810

ZNF250	**ILMN_25690**	**NM_021061.1**	**-0.877490052**	**0.971425594**	**0.00079302**
SESN3	**ILMN_11604**	**NM_144665.2**	**-0.879944442**	**1.134947842**	**0.00164548**
RIMS3	**ILMN_21581**	**NM_014747.2**	**-0.886401668**	**1.720794738**	**0.00002887**
SNX25	**ILMN_8556**	**NM_031953.2**	**-0.886699432**	**1.255349214**	**0.00001795**
JARID1B	**ILMN_14812**	**NM_006618.3**	**-0.886935531**	**1.409279841**	**0.00008858**
PITPNC1	**ILMN_12660**	**NM_181671.1**	**-0.888673165**	**1.417624087**	**0.00006531**
B4GALT1	**ILMN_20711**	**NM_001497.2**	**-0.888931145**	**0.715047063**	**0.00673477**
POLS	**ILMN_866**	**NM_006999.3**	**-0.890007455**	**1.414254344**	**0.00001205**
SLC22A17	**ILMN_10076**	**NM_016609.3**	**-0.891041878**	**0.812773625**	**0.00232667**
CABLES2	**ILMN_28000**	**NM_031215.1**	**-0.891745112**	**1.363327575**	**0.00049764**
PTPRM	**ILMN_19957**	**NM_002845.2**	**-0.892112842**	**1.097184571**	**0.00293499**
ABHD14B	**ILMN_24812**	**NM_032750.1**	**-0.892326662**	**1.44224977**	**0.00004053**
EBP	**ILMN_12837**	**NM_006579.1**	**-0.896139529**	**0.795734565**	**0.00203677**
PLCG1	**ILMN_3773**	**NM_002660.2**	**-0.896364198**	**0.806098446**	**0.00364560**
MGC11257	**ILMN_22349**	**NM_032350.3**	**-0.898197114**	**1.023241639**	**0.00025317**
THUMPD2	**ILMN_25512**	**NM_025264.2**	**-0.90015255**	**1.328191704**	**0.00000093**
CD28	**ILMN_5437**	**NM_006139.1**	**-0.901792636**	**0.970867535**	**0.00007722**
NUP155	**ILMN_1051**	**NM_004298.2**	**-0.904508354**	**1.137552613**	**0.00027759**
MASTL	**ILMN_7073**	**NM_032844.1**	**-0.906272759**	**1.009884304**	**0.00188459**
VIL2	**ILMN_2940**	**NM_003379.3**	**-0.90719828**	**0.809621514**	**0.00631678**
CDR2	**ILMN_10283**	**NM_001802.1**	**-0.907555245**	**0.920461232**	**0.00017280**
AHCTF1	**ILMN_28711**	**NM_015446.3**	**-0.907937518**	**1.662012186**	**0.00005143**
ITK	**ILMN_23317**	**NM_005546.3**	**-0.909896826**	**0.956548738**	**0.00023181**
NDST2	**ILMN_21220**	**NM_003635.2**	**-0.910459655**	**1.365135533**	**0.00022155**
ZNF317	**ILMN_22884**	**NM_020933.2**	**-0.910607633**	**1.561177388**	**0.00000864**
PAG1	**ILMN_20316**	**NM_018440.3**	**-0.914077327**	**1.177056311**	**0.00039575**
SLC25A26	**ILMN_21381**	**NM_173471.1**	**-0.914244004**	**1.329568646**	**0.00099660**
CIC	**ILMN_14588**	**NM_015125.2**	**-0.914603265**	**0.98731765**	**0.00130412**
PELP1	**ILMN_15422**	**NM_014389.1**	**-0.91485887**	**1.461682055**	**0.00004063**
KIAA1285	**ILMN_8883**	**NM_015694.1**	**-0.916008576**	**0.757897482**	**0.00508342**
LOC130074	**ILMN_26225**	**NM_001009993.1**	**-0.916194238**	**1.275412306**	**0.00013399**

HSPC023	ILMN_24668	NM_014047.1	-0.917531536	1.085247037	0.00017209
PPRC1	ILMN_10445	NM_015062.3	-0.918683753	1.44933188	0.00000951
TGIF2	ILMN_25134	NM_021809.4	-0.919181739	1.070845819	0.00620873
PPARD	ILMN_139271	NM_006238.2	-0.922036255	0.949581608	0.00400146
CHCHD6	ILMN_19685	NM_032343.1	-0.922760945	1.685898574	0.00001234
RPL41	ILMN_5276	NM_021104.1	-0.927604921	0.939022696	0.00014426
FAM100B	ILMN_22874	NM_182565.2	-0.927975076	1.381430879	0.00001068
C9orf111	ILMN_22221	NM_152286.2	-0.928190875	1.062704382	0.00034302
EDG1	ILMN_138993	NM_001400.2	-0.929786795	0.705014559	0.00565600
RCL1	ILMN_18208	NM_005772.2	-0.930738874	1.023725888	0.00021865
LMBR1L	ILMN_27914	NM_018113.1	-0.931048642	1.503486864	0.00025559
ZNF551	ILMN_4569	NM_138347.2	-0.932518582	1.640473641	0.00001422
TOE1	ILMN_11173	NM_025077.2	-0.934242822	1.467344759	0.00010122
PSME4	ILMN_10890	NM_014614.1	-0.934832764	1.295787201	0.00025690
ZBTB16	ILMN_11903	NM_006006.4	-0.935257759	0.713736086	0.00683700
DXYS155E	ILMN_26209	NM_005088.2	-0.937911248	1.224959495	0.00023307
LONRF1	ILMN_138605	NM_152271.2	-0.937927017	0.870537082	0.00948435
FLJ39779	ILMN_12781	NM_207442.1	-0.939277125	0.927670032	0.00924437
LSG1	ILMN_6416	NM_018385.1	-0.94456847	0.979257112	0.00061345
MYL5	ILMN_21416	NM_002477.1	-0.945374141	1.293359335	0.00008484
AHI1	ILMN_23469	NM_017651.3	-0.945865402	1.319394148	0.00013258
TIGA1	ILMN_2113	NM_053000.1	-0.948258709	1.60679229	0.00000074
ZNF707	ILMN_20149	NM_173831.2	-0.951346105	1.340592694	0.00053180
CDC37L1	ILMN_559	NM_017913.2	-0.954095558	1.157427303	0.00047442
ERN1	ILMN_20244	NM_152461.2	-0.954332751	0.889607391	0.00034558
DHRS3	ILMN_12432	NM_004753.4	-0.954439327	1.353544551	0.00003756
WRN	ILMN_3783	NM_000553.2	-0.954452783	1.021016836	0.00116067
URG4	ILMN_13027	NM_017920.2	-0.95589912	1.422396175	0.00007026
SMURF1	ILMN_10493	NM_181349.1	-0.95594376	0.879615015	0.00245662
HES6	ILMN_4854	NM_018645.3	-0.95725005	0.633823723	0.00695471
USF2	ILMN_6055	NM_207291.1	-0.959901269	1.066664218	0.00035373

HIVEP2	ILMN_21520	NM_006734.2	-0.960694265	1.07910833	0.00266549
CLASP1	ILMN_14659	NM_015282.1	-0.961804572	1.602890635	0.00000137
ZNF395	ILMN_17231	NM_018660.2	-0.962316466	1.309008827	0.00003382
PHF17	ILMN_1535	NM_024900.2	-0.963425077	1.235639669	0.00006684
NR1H2	ILMN_137460	NM_007121.2	-0.965620392	1.016136008	0.00208002
MGC26885	ILMN_6572	NM_152339.2	-0.965624618	0.755387041	0.00213942
DUSP14	ILMN_9561	NM_007026.1	-0.96879883	1.261528702	0.00012174
ZFP91	ILMN_4526	NM_170768.1	-0.972236704	0.893726582	0.00243123
MAX	ILMN_19642	NM_145114.1	-0.972555189	0.706014867	0.00664976
RLF	ILMN_14015	NM_012421.1	-0.973603494	1.250131376	0.00126026
PTGER4	ILMN_13328	NM_000958.2	-0.974694545	0.840103103	0.00052976
H2AFV	ILMN_29030	NM_138635.2	-0.975093302	0.871705932	0.00441653
JMJD1A	ILMN_25544	NM_018433.3	-0.975719199	1.672444208	0.00000036
CTLA4	ILMN_138103	NM_005214.2	-0.975971478	1.185608797	0.00002484
MGC3020	ILMN_29369	NM_024048.2	-0.97855089	0.708809095	0.00778758
PLXDC1	ILMN_8865	NM_020405.3	-0.981718421	1.136980201	0.00048398
EIF5A2	ILMN_4591	NM_020390.5	-0.983175786	1.370404889	0.00006957
POLR3E	ILMN_12465	NM_018119.2	-0.983687344	1.022719891	0.00225480
C14orf102	ILMN_22111	NM_017970.2	-0.983885026	0.923757623	0.00012445
SLC3A2	ILMN_7086	NM_001012661.1	-0.984338149	1.129641218	0.00042718
SOX8	ILMN_30180	NM_014587.2	-0.984450805	0.837242057	0.00251059
PDZD8	ILMN_28006	NM_173791.2	-0.987766685	1.056312215	0.00391212
GNLY	ILMN_13145	NM_012483.1	-0.988780036	0.682561583	0.00361821
KLHL15	ILMN_4687	NM_030624.1	-0.991874786	0.87533629	0.00164519
SREBF1	ILMN_21143	NM_004176.3	-0.993698671	1.055990247	0.00040999
STARD5	ILMN_18773	NM_181900.2	-0.99655088	1.272232835	0.00057152
NAT6	ILMN_29898	NM_012191.2	-0.996896062	1.52838856	0.00000474
RNMT	ILMN_23400	NM_003799.1	-0.999891612	0.895566178	0.00800594
RAPGEF1	ILMN_2480	NM_005312.2	-1.000315423	1.638795745	0.00000591
DNAH1	ILMN_7494	NM_015512.3	-1.001506589	1.481165705	0.00001576
KIAA1875	ILMN_5826	NM_032529.1	-1.001629269	1.062064033	0.00062761

DIP2A	ILMN_26238	NM_206890.1	-1.003609357	1.096616183	0.00024842
ILF3	ILMN_3208	NM_012218.2	-1.004941901	1.13215569	0.00011859
ODC1	ILMN_21065	NM_002539.1	-1.011380594	1.419153265	0.00019692
SCNN1D	ILMN_8248	NM_002978.2	-1.011891425	1.535756779	0.00001507
hCAP-H2	ILMN_12341	NM_152299.1	-1.012825824	1.353763367	0.00005147
DDX24	ILMN_10146	NM_020414.3	-1.013524336	1.062762256	0.00240995
REXO1	ILMN_20923	NM_020695.2	-1.016680475	1.407635807	0.00002962
NOP5/NOP58	ILMN_4530	NM_015934.3	-1.018736595	1.147467699	0.00023180
ABL1	ILMN_22641	NM_007313.2	-1.018830246	1.330932128	0.00048762
SLC11A2	ILMN_10129	NM_000617.1	-1.019608873	1.246218549	0.00040787
FLJ20309	ILMN_19726	NM_017759.2	-1.020911214	0.942698875	0.00971642
SERPINF1	ILMN_19076	NM_002615.4	-1.021431387	1.036808834	0.00072847
BCDIN3	ILMN_8017	NM_019606.4	-1.022041487	1.291557277	0.00000739
FBXO11	ILMN_25889	NM_018693.2	-1.022222638	1.37168029	0.00002258
COBLL1	ILMN_12678	NM_014900.3	-1.022816897	0.832426581	0.00145449
ARMC5	ILMN_5121	NM_024742.1	-1.028127871	1.39228155	0.00008707
MRPL54	ILMN_1467	NM_172251.1	-1.029024922	0.906380196	0.00022165
SNIP1	ILMN_9410	NM_024700.2	-1.030911731	1.060761685	0.00006829
Rgr	ILMN_28738	NM_153615.1	-1.034056441	1.003839655	0.00013352
STIM2	ILMN_15073	NM_020860.1	-1.035049116	1.505310421	0.00000717
TCEA3	ILMN_27218	NM_003196.1	-1.036935308	1.127944699	0.00016303
ZBTB32	ILMN_2617	NM_014383.1	-1.037514656	1.188535314	0.00063147
SPTY2D1	ILMN_1945	NM_194285.2	-1.038141646	1.175704959	0.00631444
ANKRD39	ILMN_23002	NM_016466.4	-1.038769229	1.128394344	0.00022143
ZCCHC14	ILMN_138708	NM_015144.1	-1.040336024	1.291139581	0.00034769
INADL	ILMN_3904	NM_176878.1	-1.040993021	1.70357126	0.00000101
ZBTB4	ILMN_5011	NM_020899.2	-1.042333959	2.031811569	0.00000000
C20orf7	ILMN_137958	NM_024120.2	-1.045765733	1.57133193	0.00000698
C1orf69	ILMN_10199	NM_001010867.1	-1.048307962	1.041440424	0.00247324
SLC9A8	ILMN_27475	NM_015266.1	-1.049442878	0.779587623	0.00630261
PHGDH	ILMN_5800	NM_006623.2	-1.051702862	0.758381948	0.00287671

ZBTB5	ILMN_9696	NM_014872.1	-1.051925083	1.084009802	0.00020125
SLC25A23	ILMN_5925	NM_024103.2	-1.052611559	1.331925841	0.00000234
SDCCAG33	ILMN_138968	NM_005786.3	-1.053667943	1.43026202	0.00000293
CENTD1	ILMN_4742	NM_015230.2	-1.055518062	1.14550194	0.00194764
NR3C1	ILMN_21266	NM_001018077.1	-1.056201278	1.244978969	0.00045002
IDI1	ILMN_20349	NM_004508.2	-1.056483224	1.15471556	0.00161248
CRY2	ILMN_2356	NM_021117.1	-1.056792523	1.695128921	0.00001890
AP3M2	ILMN_568	NM_006803.2	-1.059707152	0.793326683	0.00120162
FLJ22531	ILMN_1491	NM_024650.2	-1.062235578	1.084659578	0.00022721
AXIN2	ILMN_26857	NM_004655.2	-1.063024843	0.656651343	0.00432164
CDH23	ILMN_16568	NM_022124.2	-1.069963463	1.525466254	0.00033029
POFUT2	ILMN_24199	NM_133635.3	-1.072201453	1.263952694	0.00028523
SLC29A2	ILMN_26468	NM_001532.2	-1.074548495	1.110901088	0.00003703
SUPV3L1	ILMN_23318	NM_003171.2	-1.075228735	1.666073917	0.00002209
TP53BP2	ILMN_9205	NM_001031685.1	-1.078450208	1.196004171	0.00093875
PIGA	ILMN_4309	NM_020472.1	-1.079728767	0.950416392	0.00209788
HOOK1	ILMN_4311	NM_015888.3	-1.083236126	0.928616441	0.00128459
TRIM39	ILMN_8004	NM_172016.1	-1.083346862	1.564079883	0.00003575
ZNF10	ILMN_24152	NM_015394.4	-1.084199045	1.15199778	0.00100454
TGFBR3	ILMN_22620	NM_003243.2	-1.084226172	1.128488295	0.00005957
EIF2AK3	ILMN_26832	NM_004836.3	-1.085003976	1.637926145	0.00017908
ITM2C	ILMN_26681	NM_030926.4	-1.090792126	0.871247944	0.00032229
BRWD1	ILMN_28841	NM_001007246.1	-1.091496504	1.464860651	0.00017778
UBE2L3	ILMN_9533	NM_198157.1	-1.095698317	0.905055567	0.00036489
SATB1	ILMN_6836	NM_002971.2	-1.097599208	1.281847843	0.00009024
WWP2	ILMN_7085	NM_199423.1	-1.098543058	1.022921304	0.00013328
ARL4C	ILMN_15416	NM_005737.3	-1.100859167	1.576249216	0.00000041
IER2	ILMN_12423	NM_004907.2	-1.102360836	0.850031142	0.00439957
MC1R	ILMN_25536	NM_002386.2	-1.107207251	0.927711281	0.00209384
HIPK2	ILMN_29690	NM_022740.2	-1.109902186	0.848263301	0.00019985
KIAA0863	ILMN_6906	NM_014913.2	-1.110899409	1.338766228	0.00059178

TRERF1	ILMN_11192	NM_033501.1	-1.111168158	1.381573081	0.00006062
SUSD4	ILMN_139385	NM_017982.1	-1.118672404	1.052960579	0.00022936
PDE3B	ILMN_5085	NM_000922.2	-1.119749363	1.260525562	0.00003540
SFMBT1	ILMN_20774	NM_001005158.1	-1.122680128	1.054502396	0.00301102
SUPT5H	ILMN_15760	NM_003169.2	-1.123627354	1.099088419	0.00044748
RHOF	ILMN_1762	NM_019034.2	-1.126315274	1.375102017	0.00031725
LPHN1	ILMN_12017	NM_014921.3	-1.138476162	1.662119528	0.00000917
PRR8	ILMN_25238	NM_053043.1	-1.142802208	1.880951403	0.00000131
ADORA2A	ILMN_8227	NM_000675.3	-1.145913244	0.930252378	0.00199321
FBXL11	ILMN_12629	NM_012308.1	-1.148612325	1.152483839	0.00010613
KBTBD8	ILMN_23329	NM_032505.1	-1.150383237	0.980994072	0.00298577
C16orf60	ILMN_10665	NM_018455.3	-1.150703459	0.875738978	0.00533009
ARID5A	ILMN_12305	NM_212481.1	-1.153235059	1.005507042	0.00791543
BAG3	ILMN_9420	NM_004281.3	-1.155985342	1.051113868	0.00115372
PLEKHB1	ILMN_2859	NM_021200.1	-1.164061785	1.740371718	0.00000559
TNFRSF13B	ILMN_9141	NM_012452.2	-1.166101521	0.812444288	0.00410135
SLC25A34	ILMN_6699	NM_207348.1	-1.176664306	1.580127428	0.00002748
ATHL1	ILMN_138992	NM_025092.1	-1.180243174	0.927738706	0.00201768
PCTK2	ILMN_26008	NM_002595.2	-1.183475914	1.128966884	0.00538235
RNF103	ILMN_17861	NM_005667.2	-1.185091047	1.060017872	0.00079149
RSC1A1	ILMN_3431	NM_006511.1	-1.187594127	1.371416969	0.00063915
ATP2A3	ILMN_6507	NM_174958.1	-1.189495716	1.110979165	0.00436586
PRC1	ILMN_20465	NM_003981.2	-1.190523415	1.021719819	0.00448523
DUSP10	ILMN_14599	NM_144728.1	-1.195047913	1.017629683	0.00887235
ZFAND2A	ILMN_21900	NM_182491.1	-1.205318845	1.015840107	0.00003574
LMTK2	ILMN_18423	NM_014916.2	-1.206957664	1.253180161	0.00047386
P2RY11	ILMN_12237	NM_002566.4	-1.210259935	1.06355304	0.00041398
RNF10	ILMN_14490	NM_014868.3	-1.210970564	1.237971365	0.00020066
AQP3	ILMN_22744	NM_004925.3	-1.211854338	1.043353942	0.00058054
LRIG1	ILMN_3319	NM_015541.2	-1.215707138	0.887627098	0.00096494
P2RY8	ILMN_28691	NM_178129.3	-1.219269472	0.98848837	0.00126480

MLL5	ILMN_9061	NM_018682.2	-1.223435851	1.970466771	0.00001201
NGFRAP1	ILMN_7162	NM_014380.1	-1.232227015	1.206946971	0.00003357
TKTL1	ILMN_23315	NM_012253.2	-1.234316429	0.966661273	0.00017305
C6orf105	ILMN_10287	NM_032744.1	-1.239896797	0.885246225	0.00101737
RAB33A	ILMN_2535	NM_004794.2	-1.241055838	1.260240271	0.00047052
SPRY2	ILMN_19344	NM_005842.2	-1.241885867	1.021584875	0.00382667
CXCR3	ILMN_2029	NM_001504.1	-1.244115035	0.979138466	0.00777972
PIM3	ILMN_19535	NM_001001852.2	-1.246301965	0.788206936	0.00461466
PLEKHF1	ILMN_25787	NM_024310.2	-1.250552556	1.09224994	0.00017933
ZNF335	ILMN_9501	NM_022095.3	-1.251403176	1.416122752	0.00000374
TIPARP	ILMN_4419	NM_015508.2	-1.252940769	0.785601153	0.00628327
PAPD5	ILMN_11715	NM_022447.1	-1.25411808	1.168275365	0.00090546
GJA12	ILMN_6786	NM_020435.2	-1.257644209	1.320134633	0.00016783
CRSP7	ILMN_14940	NM_004831.3	-1.261907049	1.49395962	0.00002501
C1orf21	ILMN_26434	NM_030806.3	-1.265697427	1.169849	0.00014912
MIDN	ILMN_6472	NM_177401.4	-1.266404108	0.946703753	0.00048229
PASK	ILMN_19873	NM_015148.2	-1.276320058	0.912776289	0.00129334
CAMK4	ILMN_5327	NM_001744.3	-1.277241788	1.681851719	0.00001813
LOC90826	ILMN_17873	NM_138364.2	-1.27785886	1.15131075	0.00042332
ATXN7L2	ILMN_6796	NM_153340.2	-1.280663545	1.891153588	0.00000392
PTP4A1	ILMN_27315	NM_003463.3	-1.280755813	0.981037923	0.00088222
OVGP1	ILMN_7829	NM_002557.3	-1.280833537	2.145648028	0.00000006
SMPD1	ILMN_8257	NM_001007593.1	-1.28381406	1.165957203	0.00027206
CHD7	ILMN_29669	NM_017780.2	-1.285175635	1.611575053	0.00000132
ZHX2	ILMN_21703	NM_014943.3	-1.289399266	1.042646216	0.00479687
TSEN54	ILMN_8569	NM_207346.1	-1.291774	1.143237204	0.00080973
KTN1	ILMN_29637	NM_182926.1	-1.292563877	1.679784537	0.00002236
RWDD1	ILMN_29174	NM_016104.2	-1.295908497	1.158676772	0.00112686
NXT1	ILMN_26865	NM_013248.2	-1.303466657	1.103790462	0.00006758
IRF4	ILMN_12414	NM_002460.1	-1.304331316	2.190738858	0.00000044
UAP1	ILMN_7007	NM_003115.3	-1.304761251	1.006944977	0.00035277

ANKRD37	**ILMN_2423**	**NM_181726.1**	**-1.308150345**	**0.937315673**	**0.00117745**
DDX6	**ILMN_25649**	**NM_004397.3**	**-1.309650532**	**2.155651735**	**0.00000030**
LOC285513	**ILMN_8803**	**NM_198281.1**	**-1.313429509**	**1.546964677**	**0.00004860**
TAF5L	**ILMN_6399**	**NM_001025247.1**	**-1.32305886**	**1.278931108**	**0.00002693**
CMTM8	**ILMN_19577**	**NM_178868.3**	**-1.325376459**	**0.983647445**	**0.00014993**
SLC16A10	**ILMN_10556**	**NM_018593.3**	**-1.327387377**	**1.108265763**	**0.00009461**
FYTTD1	**ILMN_5513**	**NM_001011537.1**	**-1.329127617**	**1.018346452**	**0.00030597**
PBX4	**ILMN_11004**	**NM_025245.1**	**-1.333943281**	**0.986938102**	**0.00304411**
PTGDS	**ILMN_19248**	**NM_000954.5**	**-1.334379734**	**0.752694849**	**0.00189346**
TNFSF9	**ILMN_18030**	**NM_003811.2**	**-1.335068602**	**1.026974042**	**0.00016772**
CNN3	**ILMN_15358**	**NM_001839.2**	**-1.338292839**	**1.112524436**	**0.00048147**
ZNF295	**ILMN_12236**	**NM_020727.3**	**-1.353598502**	**1.304194049**	**0.00116404**
CCR7	**ILMN_21335**	**NM_001838.2**	**-1.359887042**	**0.918007418**	**0.00010383**
BCL9L	**ILMN_27555**	**NM_182557.1**	**-1.360587969**	**1.095800517**	**0.00209233**
IRF2BP2	**ILMN_5645**	**NM_182972.1**	**-1.370069134**	**0.969007786**	**0.00052119**
RASA3	**ILMN_19501**	**NM_007368.2**	**-1.3738673**	**1.455515605**	**0.00002815**
ARRDC2	**ILMN_7560**	**NM_015683.1**	**-1.379096994**	**1.114271453**	**0.00156368**
SCML1	**ILMN_138894**	**NM_006746.3**	**-1.381562596**	**1.167403576**	**0.00101569**
KIAA1754	**ILMN_13119**	**NM_033397.2**	**-1.38432219**	**1.12747861**	**0.00001941**
MAL	**ILMN_3411**	**NM_002371.2**	**-1.393195146**	**2.207099447**	**0.00000002**
MCM6	**ILMN_15791**	**NM_005915.4**	**-1.39751721**	**1.334945131**	**0.00049304**
H2AFX	**ILMN_23585**	**NM_002105.2**	**-1.40589861**	**1.466002611**	**0.00007136**
ZFPM1	**ILMN_15190**	**NM_153813.1**	**-1.407664057**	**0.875418487**	**0.00457871**
EIF2C2	**ILMN_25413**	**NM_012154.2**	**-1.417512796**	**1.665176969**	**0.00023257**
BHLHB2	**ILMN_24095**	**NM_003670.1**	**-1.418174041**	**0.889047464**	**0.00057911**
BRD1	**ILMN_19562**	**NM_014577.1**	**-1.428483553**	**1.226958019**	**0.00098546**
CTH	**ILMN_4045**	**NM_153742.3**	**-1.429744393**	**0.844427128**	**0.00829306**
FLT3LG	**ILMN_4754**	**NM_001459.2**	**-1.440491375**	**1.580411425**	**0.00003438**
SLC5A6	**ILMN_880**	**NM_021095.1**	**-1.45459924**	**1.033310371**	**0.00195215**
DYNLL2	**ILMN_28971**	**NM_080677.1**	**-1.455649083**	**1.2977239**	**0.00000465**
GMEB2	**ILMN_7174**	**NM_012384.2**	**-1.456946954**	**1.247345988**	**0.00010064**

GADD45B	**ILMN_138334**	**NM_015675.1**	**-1.45927016**	**0.890864982**	**0.00134377**
PRR7	**ILMN_17178**	**NM_030567.2**	**-1.459895146**	**1.238910411**	**0.00004716**
NR1D2	**ILMN_17981**	**NM_005126.2**	**-1.463555459**	**1.152704022**	**0.00084447**
CD69	**ILMN_13491**	**NM_001781.1**	**-1.467514074**	**0.767635098**	**0.00431058**
FASN	**ILMN_16128**	**NM_004104.4**	**-1.474522935**	**0.834416803**	**0.00868356**
LOC158830	**ILMN_10937**	**NM_001025265.1**	**-1.492231983**	**1.659484712**	**0.00001229**
K6IRS2	**ILMN_14872**	**NM_080747.1**	**-1.494649542**	**0.857170698**	**0.00873840**
BTG3	**ILMN_27215**	**NM_006806.3**	**-1.49479515**	**1.027933063**	**0.00054222**
BZRAP1	**ILMN_11647**	**NM_004758.1**	**-1.502581736**	**1.509465143**	**0.00001428**
OCIAD2	**ILMN_18246**	**NM_152398.2**	**-1.50757606**	**1.074604795**	**0.00011990**
MN1	**ILMN_3720**	**NM_002430.2**	**-1.509096037**	**1.199838716**	**0.00627720**
CASZ1	**ILMN_27214**	**NM_017766.2**	**-1.513362235**	**1.067663278**	**0.00068936**
MGAT4A	**ILMN_139237**	**NM_012214.1**	**-1.516364202**	**1.43878649**	**0.00001522**
DHCR7	**ILMN_2138**	**NM_001360.1**	**-1.521006311**	**1.09154978**	**0.00456684**
FLJ20152	**ILMN_139301**	**NM_019000.2**	**-1.529809004**	**1.570604078**	**0.00000028**
VPS37B	**ILMN_18457**	**NM_024667.1**	**-1.535202738**	**1.386660943**	**0.00053514**
MGC17330	**ILMN_15026**	**NM_052880.3**	**-1.549289306**	**2.615593431**	**0.00000000**
ICOS	**ILMN_9996**	**NM_012092.2**	**-1.55851009**	**0.844005633**	**0.00079917**
HIVEP1	**ILMN_6517**	**NM_002114.1**	**-1.565011369**	**1.269386062**	**0.00098464**
DUSP5	**ILMN_12928**	**NM_004419.3**	**-1.568760545**	**0.926620188**	**0.00027977**
CCRN4L	**ILMN_2730**	**NM_012118.2**	**-1.57585378**	**0.676954674**	**0.00941115**
DDIT3	**ILMN_16748**	**NM_004083.4**	**-1.580473683**	**0.904339432**	**0.00035640**
SFXN1	**ILMN_16398**	**NM_022754.4**	**-1.584214832**	**1.584567288**	**0.00000684**
IL23A	**ILMN_20797**	**NM_016584.2**	**-1.592231591**	**1.093073096**	**0.00085884**
CD248	**ILMN_25766**	**NM_020404.2**	**-1.593596823**	**1.03796568**	**0.00063579**
TMEM119	**ILMN_30233**	**NM_181724.1**	**-1.610801334**	**1.060679622**	**0.00207322**
ICOSLG	**ILMN_28135**	**NM_015259.4**	**-1.614784424**	**1.230937184**	**0.00073267**
IL21R	**ILMN_12364**	**NM_181078.1**	**-1.616207162**	**1.128564536**	**0.00074184**
DDX39	**ILMN_23076**	**NM_138998.1**	**-1.658427147**	**1.367587877**	**0.00016648**
FHIT	**ILMN_15082**	**NM_002012.1**	**-1.673448104**	**1.828433492**	**0.00000036**
MTP18	**ILMN_13393**	**NM_016498.3**	**-1.68269229**	**1.49713298**	**0.00000170**

BRD4	ILMN_22169	NM_058243.1	-1.683630214	1.449106676	0.00043360
SPRY1	ILMN_23549	NM_199327.1	-1.685777091	1.00030247	0.00128713
PPP1R16B	ILMN_26824	NM_015568.2	-1.718794599	1.300533176	0.00009419
NAP1L5	ILMN_5355	NM_153757.1	-1.728193379	1.563329697	0.00001555
TNF	ILMN_24087	NM_000594.2	-1.730636109	0.817723797	0.00871514
RGC32	ILMN_14549	NM_014059.1	-1.735045244	1.070386047	0.00010195
ZC3H12A	ILMN_18682	NM_025079.1	-1.735762717	0.908241129	0.00147260
MXRA7	ILMN_628	NM_001008528.1	-1.748431483	0.973613834	0.00291921
SC5DL	ILMN_24287	NM_006918.3	-1.750481592	1.151071938	0.00272999
UBE2D2	ILMN_27792	NM_181838.1	-1.757736084	1.428181422	0.00001760
FGF9	ILMN_1771	NM_002010.1	-1.760403535	1.428823821	0.00010647
YES1	ILMN_1505	NM_005433.3	-1.764392963	1.318925488	0.00039148
ZNF165	ILMN_8007	NM_003447.2	-1.773928105	1.218351034	0.00025979
CCNT1	ILMN_1033	NM_001240.2	-1.786747551	1.130941224	0.00097410
PLK3	ILMN_8968	NM_004073.2	-1.790107415	1.123509115	0.00033397
JOSD1	ILMN_21647	NM_014876.3	-1.797931989	1.466413364	0.00000333
SOCS1	ILMN_3038	NM_003745.1	-1.807780801	0.953587459	0.00028092
AVPI1	ILMN_9920	NM_021732.1	-1.844873323	0.793664838	0.00101944
TSPYL2	ILMN_26999	NM_022117.1	-1.845460576	1.080002715	0.00158472
GPR132	ILMN_19860	NM_013345.2	-1.858180805	1.009628034	0.00276606
FGFRL1	ILMN_28273	NM_001004358.1	-1.86348235	1.136549742	0.00251797
AXUD1	ILMN_6524	NM_033027.2	-1.865941039	1.074402426	0.00002134
DBH	ILMN_25962	NM_000787.2	-1.875893893	1.358238144	0.00199035
GADD45G	ILMN_4884	NM_006705.2	-1.883200245	1.092648505	0.00159004
ELL2	ILMN_15317	NM_012081.3	-1.895230568	1.054802224	0.00923017
JUN	ILMN_7746	NM_002228.3	-1.93716324	1.35200957	0.00128284
CD5	ILMN_29547	NM_014207.2	-1.969005232	1.472172181	0.00001058
CCL4L1	ILMN_4819	NM_001001435.2	-1.972847504	0.777562423	0.00687597
DLL1	ILMN_1802	NM_005618.2	-1.981683353	1.243856495	0.00029039
TP53INP2	ILMN_28839	NM_021202.1	-1.987037329	0.803135548	0.00674516
GADD45A	ILMN_17355	NM_001924.2	-2.011561809	1.213085063	0.00005108

USP36	ILMN_23872	NM_025090.2	-2.045788282	1.389118043	0.00005194
DUSP2	ILMN_21154	NM_004418.2	-2.049331375	0.994473822	0.00002824
GRASP	ILMN_11141	NM_181711.1	-2.181045645	0.887808594	0.00177700
JMY	ILMN_18491	NM_152405.1	-2.214065556	1.559103931	0.00008945
SMAD7	ILMN_10011	NM_005904.2	-2.21499091	1.138519775	0.00041631
DUSP8	ILMN_21272	NM_004420.1	-2.243733266	1.648409441	0.00000576
K6IRS3	ILMN_13180	NM_175068.2	-2.248575123	1.805083434	0.00001610
FOSB	ILMN_13603	NM_006732.1	-2.312871227	0.610878937	0.00583158
GALR2	ILMN_4188	NM_003857.2	-2.318004128	1.295873257	0.00007526
NR4A2	ILMN_28405	NM_006186.2	-2.375127134	0.693171084	0.00105245
CXCL2	ILMN_29078	NM_002089.1	-2.384946205	0.672530598	0.00878350
SEC14L2	ILMN_9607	NM_012429.1	-2.389587687	1.369976121	0.00048088
CD83	ILMN_16705	NM_004233.2	-2.400607006	1.029167736	0.00012951
PHACTR1	ILMN_17425	NM_030948.1	-2.411791103	1.475392994	0.00001304
LYPD3	ILMN_23267	NM_014400.1	-2.421971695	1.147458967	0.00050112
GJB2	ILMN_26343	NM_004004.3	-2.549537207	0.975329581	0.00657606
ZNF331	ILMN_28697	NM_018555.4	-2.578337112	1.033044629	0.00014144
RGS16	ILMN_16445	NM_002928.2	-2.807399525	0.923543594	0.00132862
PHLDA1	ILMN_138322	NM_007350.2	-2.954967361	0.995309086	0.00237317
SLC7A5	ILMN_25446	NM_003486.5	-3.70325022	1.269118167	0.00008773

Supp. Table S2E. probe-level intersections between the *PTPN11*, *SOS1* and *SHOC2* signatures

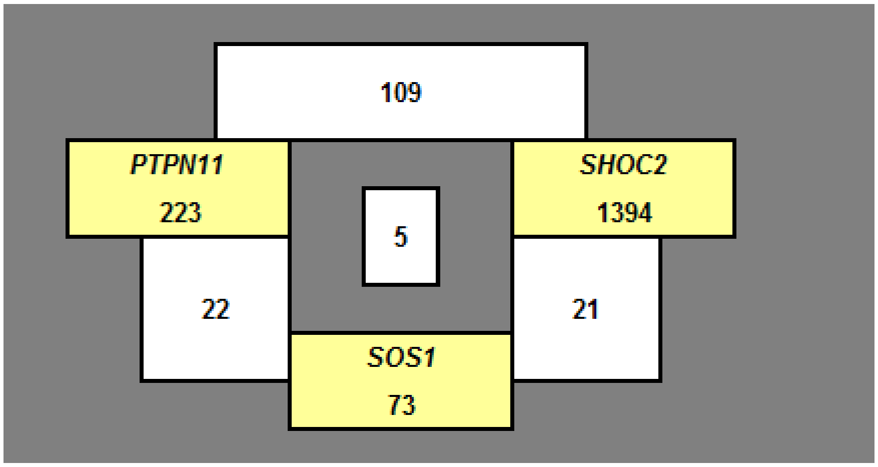

Supp. Table S3. False Discovery rate analysis				
	**NS Signature**	***PTPN11* Signature**	***SOS1* Signature**	***SHOC2* Signature**
**True positives**	**125**	**225**	**73**	**1407**
**Median false positives in 2000 permutations**	**0**	**1**	**4**	**5**
**FDR (Median)**	**0.0%**	**0.4%**	**5.5%**	**0.4%**
**Median false positives in 2000 permutations**	**0.227**	**3.203**	**12.082**	**15.514**
**FDR (Average)**	**0.2%**	**1.4%**	**16.6%**	**1.1%**

Supp. Table S4A.
Functional annotation analysis (DAVID EASE) of the *PTPN11* Signature

***PTPN11* SIGNATURE (up-regulated & downregulated genes)**

Category	Term	Pvalue	n° of Genes	Fold Enrich-ment	Benjamini-corrected p-value
SP_PIR_KEYWORDS	receptor	1.06E-05	24	2.805	3.97E-04
SP_PIR_KEYWORDS	SH2 domain	9.38E-06	11	5.971	4.01E-04
SP_PIR_KEYWORDS	tyrosine-specific protein kinase	2.88E-04	5	13.570	7.80E-03
SP_PIR_KEYWORDS	disulfide bond	1.22E-18	57	3.683	3.65E-16
UP_SEQ_FEATURE	disulfide bond	2.96E-18	55	3.740	2.00E-15
SP_PIR_KEYWORDS	signal	2.06E-14	58	2.958	3.09E-12
UP_SEQ_FEATURE	signal peptide	2.11E-14	58	2.956	7.12E-12
GOTERM_CC_FAT	GO:0005886~plasma membrane	5.24E-13	71	2.258	1.24E-10
SP_PIR_KEYWORDS	Secreted	3.60E-11	30	4.236	3.59E-09
UP_SEQ_FEATURE	topological domain:Extracellular	2.97E-11	45	2.993	6.69E-09
GOTERM_CC_FAT	GO:0005576~extracellular region	1.14E-10	36	3.335	1.35E-08
SP_PIR_KEYWORDS	glycoprotein	3.72E-10	61	2.275	2.78E-08
GOTERM_CC_FAT	GO:0044421~extracellular region part	1.37E-08	22	4.316	1.08E-06
GOTERM_CC_FAT	GO:0005615~extracellular space	2.03E-08	20	4.669	1.20E-06
SP_PIR_KEYWORDS	Immunoglobulin domain	5.04E-08	17	5.393	3.01E-06
GOTERM_CC_FAT	GO:0031226~intrinsic to plasma membrane	6.69E-08	29	3.106	3.17E-06
INTERPRO	IPR013783:Immunoglobulin-like fold	9.09E-09	19	5.258	4.46E-06
UP_SEQ_FEATURE	glycosylation site:N-linked (GlcNAc...)	3.81E-08	54	2.158	6.44E-06
SP_PIR_KEYWORDS	cell membrane	4.06E-07	35	2.567	2.03E-05
GOTERM_CC_FAT	GO:0005887~integral to plasma membrane	5.93E-07	27	2.959	2.34E-05

UP_SEQ_FEATURE	topological domain:Cytoplasmic	2.57E-07	51	2.099	3.47E-05
GOTERM_CC_FAT	GO:0044459~plasma membrane part	6.18E-06	41	2.045	2.09E-04
INTERPRO	IPR013106:Immunoglobulin V-set	3.28E-06	10	7.611	8.05E-04
INTERPRO	IPR000980:SH2 motif	7.60E-06	11	6.089	1.24E-03
INTERPRO	IPR003599:Immunoglobulin subtype	1.19E-05	12	5.219	1.46E-03
GOTERM_CC_FAT	GO:0009986~cell surface	5.32E-05	14	3.801	1.58E-03
INTERPRO	IPR007110:Immunoglobulin-like	2.75E-05	13	4.397	2.70E-03
GOTERM_CC_FAT	GO:0009897~external side of plasma membrane	1.03E-04	10	5.075	2.72E-03
SP_PIR_KEYWORDS	transmembrane protein	9.42E-05	17	3.099	3.13E-03
GOTERM_CC_FAT	GO:0016021~integral to membrane	2.35E-04	72	1.428	5.57E-03
GOTERM_BP_FAT	GO:0002684~positive regulation of immune system process	3.89E-06	15	4.456	6.37E-03
SP_PIR_KEYWORDS	transmembrane	2.86E-04	66	1.505	8.51E-03
SP_PIR_KEYWORDS	membrane	3.99E-04	86	1.381	9.88E-03

***PTPN11* SIGNATURE (up-regulated genes)**

Category	Term	Pvalue	n° Gene	Fold Enrich	Benjamini p-value
UP_SEQ_FEATURE	disulfide bond	1.72E-10	27	4.271	6.50E-08
SP_PIR_KEYWORDS	disulfide bond	5.34E-10	27	4.059	1.08E-07
GOTERM_CC_FAT	GO:0005886~plasma membrane	6.49E-09	36	2.589	1.11E-06
SP_PIR_KEYWORDS	signal	3.32E-07	26	3.084	3.37E-05
UP_SEQ_FEATURE	signal peptide	3.35E-07	26	3.083	6.32E-05
SP_PIR_KEYWORDS	glycoprotein	3.29E-06	29	2.516	2.23E-04
SP_PIR_KEYWORDS	Secreted	7.51E-06	14	4.598	3.81E-04
GOTERM_CC_FAT	GO:0005615~extracellular space	1.20E-05	11	5.806	6.83E-04
GOTERM_CC_FAT	GO:0005576~extracellular region	9.63E-06	17	3.561	8.23E-04
UP_SEQ_FEATURE	glycosylation site:N-linked (GlcNAc...)	9.17E-06	27	2.510	1.15E-03

GOTERM_CC_FAT	GO:0044421~extracellular region part	5.57E-05	11	4.879	2.38E-03
GOTERM_CC_FAT	GO:0044459~plasma membrane part	2.14E-04	21	2.368	7.30E-03
***PTPN11* SIGNATURE (down-regulated genes)**					
**Category**	**Term**	**Pvalue**	**n° of Genes**	**Fold Enrich-ment**	**Benjamini-corrected p-value**
SP_PIR_KEYWORDS	disulfide bond	4.79E-09	30	3.400	1.01E-06
UP_SEQ_FEATURE	topological domain:Extracellular	1.07E-08	29	3.383	4.05E-06
UP_SEQ_FEATURE	disulfide bond	2.83E-08	28	3.340	5.33E-06
SP_PIR_KEYWORDS	signal	7.35E-08	32	2.862	7.71E-06
UP_SEQ_FEATURE	signal peptide	7.44E-08	32	2.860	9.35E-06
SP_PIR_KEYWORDS	Secreted	8.81E-06	16	3.962	6.17E-04
UP_SEQ_FEATURE	topological domain:Cytoplasmic	8.12E-06	32	2.311	7.65E-04
GOTERM_CC_FAT	GO:0009986~cell surface	2.60E-05	11	5.354	1.02E-03
SP_PIR_KEYWORDS	cell membrane	2.03E-05	22	2.830	1.07E-03
GOTERM_CC_FAT	GO:0005576~extracellular region	1.53E-05	19	3.156	1.20E-03
GOTERM_CC_FAT	GO:0005886~plasma membrane	2.32E-05	35	1.996	1.22E-03
GOTERM_CC_FAT	GO:0009897~external side of plasma membrane	8.74E-06	9	8.190	1.37E-03
SP_PIR_KEYWORDS	SH2 domain	6.59E-05	8	7.616	2.30E-03
SP_PIR_KEYWORDS	glycoprotein	5.89E-05	32	2.093	2.47E-03
GOTERM_CC_FAT	GO:0031226~intrinsic to plasma membrane	1.35E-04	16	3.072	4.24E-03
GOTERM_BP_FAT	GO:0042110~T cell activation	7.26E-06	10	7.115	4.31E-03
GOTERM_BP_FAT	GO:0046649~lymphocyte activation	4.17E-06	12	5.831	4.95E-03
SP_PIR_KEYWORDS	transmembrane protein	2.43E-04	12	3.837	7.27E-03
GOTERM_BP_FAT	GO:0045321~leukocyte activation	1.97E-05	12	4.980	7.77E-03
GOTERM_CC_FAT	GO:0044421~extracellular region part	4.12E-04	11	3.870	9.21E-03
SP_PIR_KEYWORDS	Immunoglobulin domain	3.64E-04	9	5.007	9.52E-03

GOTERM_CC_FAT	GO:0005887~integral to plasma membrane	3.76E-04	15	2.948	9.79E-03

Supp. Table S4B.
Functional annotation analysis (DAVID EASE) of the *SHOC2* signature

***SHOC2* Signature (up-regulated & downregulated genes)**

Category	Term	Pvalue	n° of Genes	Fold Enrich-ment	Benjamini-corrected p-value
UP_SEQ_FEATURE	topological domain:Cytoplasmic	8.09E-07	203	1.333	2.48E-03
UP_SEQ_FEATURE	topological domain:Extracellular	2.15E-06	134	1.422	3.30E-03
GOTERM_CC_FAT	GO:0005886~plasma membrane	1.03E-05	237	1.248	5.64E-03
GOTERM_CC_FAT	GO:0044459~plasma membrane part	2.62E-05	159	1.314	7.14E-03
UP_SEQ_FEATURE	glycosylation site:N-linked (GlcNAc...)	8.53E-06	203	1.294	8.69E-03

***SHOC2* Signature (up-regulated genes)**

Category	Term	Pvalue	n° of Genes	Fold Enrich-ment	Benjamini-corrected p-value
SP_PIR_KEYWORDS	transmembrane	2.16E-10	183	1.510	9.23E-08
UP_SEQ_FEATURE	transmembrane region	1.05E-10	183	1.522	1.52E-07
SP_PIR_KEYWORDS	membrane	2.30E-08	232	1.348	4.91E-06
UP_SEQ_FEATURE	topological domain:Cytoplasmic	8.36E-08	109	1.623	6.06E-05
SP_PIR_KEYWORDS	glycoprotein	1.36E-06	113	1.524	1.94E-04
UP_SEQ_FEATURE	glycosylation site:N-linked (GlcNAc...)	4.15E-07	109	1.575	2.01E-04
GOTERM_CC_FAT	GO:0031224~intrinsic to membrane	6.23E-06	197	1.280	1.23E-03
GOTERM_CC_FAT	GO:0016021~integral to membrane	4.19E-06	193	1.293	1.65E-03
SP_PIR_KEYWORDS	immune response	2.13E-05	22	2.738	2.27E-03

SP_PIR_KEYWORDS	transport	2.81E-05	93	1.502	2.40E-03
UP_SEQ_FEATURE	topological domain:Extracellular	8.20E-06	70	1.684	2.97E-03
GOTERM_CC_FAT	GO:0005773~vacuole	6.96E-05	28	2.207	9.13E-03
UP_SEQ_FEATURE	binding site:Substrate	3.21E-05	29	2.286	9.27E-03
***SHOC2* Signature (down-regulated genes)**					
**Category**	**Term**	**Pvalue**	**n° of Genes**	**Fold Enrich-ment**	**Benjamini-corrected p-value**
SP_PIR_KEYWORDS	Transcription	2.71E-11	168	1.592	1.18E-08
SP_PIR_KEYWORDS	transcription regulation	5.48E-11	161	1.600	1.19E-08
SP_PIR_KEYWORDS	nucleus	4.28E-10	318	1.313	6.20E-08
SP_PIR_KEYWORDS	dna-binding	1.94E-09	129	1.635	2.11E-07
GOTERM_BP_FAT	GO:0006350~transcription	1.25E-10	171	1.533	3.40E-07
GOTERM_BP_FAT	GO:0045449~regulation of transcription	1.87E-09	188	1.448	2.54E-06
SP_PIR_KEYWORDS	zinc-finger	3.15E-07	131	1.504	2.74E-05
INTERPRO	IPR007087:Zinc finger, C2H2-type	3.54E-08	65	1.969	3.92E-05
INTERPRO	IPR015880:Zinc finger, C2H2-like	2.02E-07	65	1.887	1.12E-04
UP_SEQ_FEATURE	zinc finger region:C2H2-type 2	7.01E-07	52	1.997	1.38E-03
UP_SEQ_FEATURE	zinc finger region:C2H2-type 4	3.43E-06	47	1.980	3.38E-03
UP_SEQ_FEATURE	zinc finger region:C2H2-type 3	5.81E-06	49	1.914	3.81E-03
UP_SEQ_FEATURE	zinc finger region:C2H2-type 5	2.78E-05	42	1.921	1.36E-02
UP_SEQ_FEATURE	zinc finger region:C2H2-type 1	3.68E-05	45	1.851	1.44E-02
SP_PIR_KEYWORDS	zinc	2.02E-04	140	1.316	1.46E-02
UP_SEQ_FEATURE	zinc finger region:C2H2-type 7	4.55E-05	35	2.028	1.48E-02
UP_SEQ_FEATURE	compositionally biased region:Pro-rich	6.13E-05	61	1.644	1.71E-02
GOTERM_BP_FAT	GO:0006355~regulation of transcription, DNA-dependent	2.33E-05	113	1.426	2.08E-02
GOTERM_BP_FAT	GO:0051252~regulation of RNA metabolic process	3.73E-05	114	1.409	2.50E-02

Supp. Table S5.
Modulation of transcription factors and their putative target genes

Transcription Factor (TF)	TF Modulation	TF binding site enrichment	Opossum Z-Score	Opossum p-value	Hypergeometric analysis p-value
GFI1	**Downregulated in *PTPN11* and *SOS1* mutation group**	**Enriched in *PTPN11*-downmodulated genes**	**5.033**	**0.0003142**	**p<0.0001**
GABPA	**Upregulated in *SOS1* and *SHOC2* mutation group**	**Enriched in *SOS1*-downmodulated genes**	**8.295**	**0.02257**	**p<0.05**
CREB1	**Upregulated in *SHOC2* mutation group**	**Enriched in *SHOC2*-downmodulated genes**	**14.32**	**0.00144**	**p<0.00001**
SP1	**Upregulated in *SHOC2* mutation group**	**Enriched in SHOC2-downmodulated genes**	**7.007**	**0.007963**	**p<0.00001**
